# Recent updates on potential of VEGFR-2 small-molecule inhibitors as anticancer agents

**DOI:** 10.1039/d4ra05244g

**Published:** 2024-10-22

**Authors:** Prashant Jagannath Chaudhari, Aditya Ramchandra Nemade, Atul Arun Shirkhedkar

**Affiliations:** a Department of Pharmaceutical Chemistry, R. C. Patel Institute of Pharmaceutical Education and Research Shirpur, Dist-Dhule Maharashtra 425 405 India prashantniperk@gmail.com; b Department of Chemistry, Carnegie Mellon University 4400 Fifth Avenue Pittsburgh Pennsylvania 15213 USA; c Department of Pharmaceutics, M.S. Ramaiah University of Applied Sciences Bengaluru Karnataka 560054 India

## Abstract

The vascular endothelial growth factor receptor (VEGFR) system is the key component for controlling angiogenesis in cancer cells. Blocking vascular endothelial growth factor receptor 2 (VEGFR2) signalling is one of the most promising approaches to hindering angiogenesis and the subsequent growth of cancer cells. The USFDA-approved small-molecule drugs targeting VEGFR-2 are developing drug resistance over the course of chemotherapy, and cardiac-related side effects are consistently being reported; hence, there is an urgent need for more safe and effective anticancer molecules. The present review focuses on the structure and physiology of VEGFR-2 and its involvement in the progression of cancer cells. The recent updates from the last five years through papers and patents on structure–activity relationships, pharmacophoric attributes, molecular docking interactions, antiangiogenic assays, cancer cell line studies, and the potencies (IC_50_) of VEGFR-2 inhibitors are discussed herein. The common structural framework requirements, such as the Asp-Phe-Gly (DFG) motif of VEGFR-2 interacting with the HBD–HBA region in the ligand molecules, the central aryl ring occupying the linker region, and a variety of bio-isosteres, can enhance activity against VEGFR-2. At one end, the heteroaryl moiety is essential for interaction within the ATP-binding site of VEGFR-2, while the terminal hydrophobic tail occupies the allosteric binding site. Three to five bond spacers between the heteroaryl and HBD–HBA regions provided a better result towards VEGFR-2 inhibition, mirroring the behaviors of standard drugs. The in-depth analysis of recent updates on VEGFR-2 inhibitors presented in this paper will help prospective synthetic and medicinal chemists to discover new lead molecules for the treatment of various cancers.

## Abstract 2

Essential structural motifs for design of Lead against VEGFR-2.

## Graphical abstract

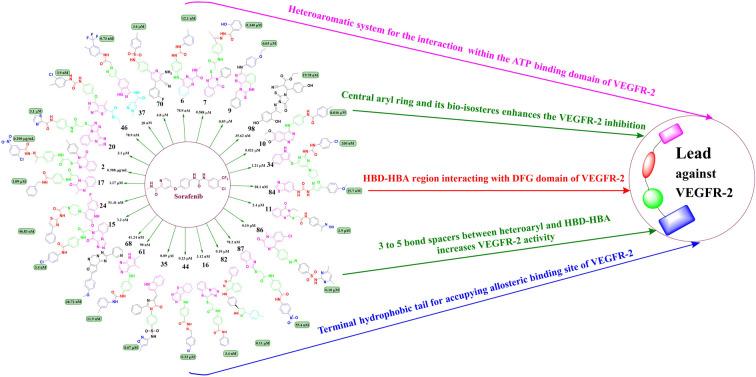

## 1. Introduction

Cancer killed over 10 million people worldwide in 2020, or one in six.^1^ By 2040, there will be an additional 29.5 million new cases of cancer, according to the world health organization (WHO).^2^ The administration of antibodies and small-molecules for cancer chemotherapy are currently the two main strategies used in targeted cancer treatment. Despite their often-high selectivity, antibodies are limited in their ability to reach deep tissues because of their large molecular weight.^3^ Small-molecule inhibitors have significantly transformed the field of drug design and development in recent decades. This is because they possess the capability to effectively bind to a wider range of targets, both inside and outside of cells.^4^

Clinical investigation and drug resistance studies show that single targeting may not always have the desired biological effect, even when the target is inactivated or inhibited. Targeting a single oncoprotein may not generate long-term remission, hence studying biological networks for cancer is essential.^5^ Two prominent approaches are utilized for developing multi-targeting therapies. First, combining drugs aimed at different targets for an additive or synergistic effect.^6^ The second strategy is to develop a single drug with multiple targets to effectively block the numerous carcinogenic pathways.^7^ The search for a single agent that can act on two or more targets at the same time is a crucial aspect of the process of developing multi-targeting therapies.

### 1.1 Angiogenesis inhibition: a promising approach in anticancer therapy

Angiogenesis is the process by which primary solid tumours generate new blood capillaries to feed nutrition and oxygen, remove metabolic waste, and accelerate metastasis.^8^ John Hunter, a renowned British surgeon from the 18th century, gained widespread recognition for coining the term 'angiogenesis' in his writings.^9^ Angiogenesis, the physiological mechanism through which new blood vessels are generated from existing ones, plays a vital role in female reproductive health, cellular proliferation, wound healing, and tissue regeneration.^10^ The regulation of angiogenesis has a significant role in the development of several disorders, such as cancer, atherosclerosis, diabetes, and rheumatoid arthritis.^11^ As newly formed blood vessels offer oxygen and vital nutrients, they enable tumour growth and subsequently aid in the onset of metastasis, which leads to mortality in several cancers.^12^

**Fig. 1:**
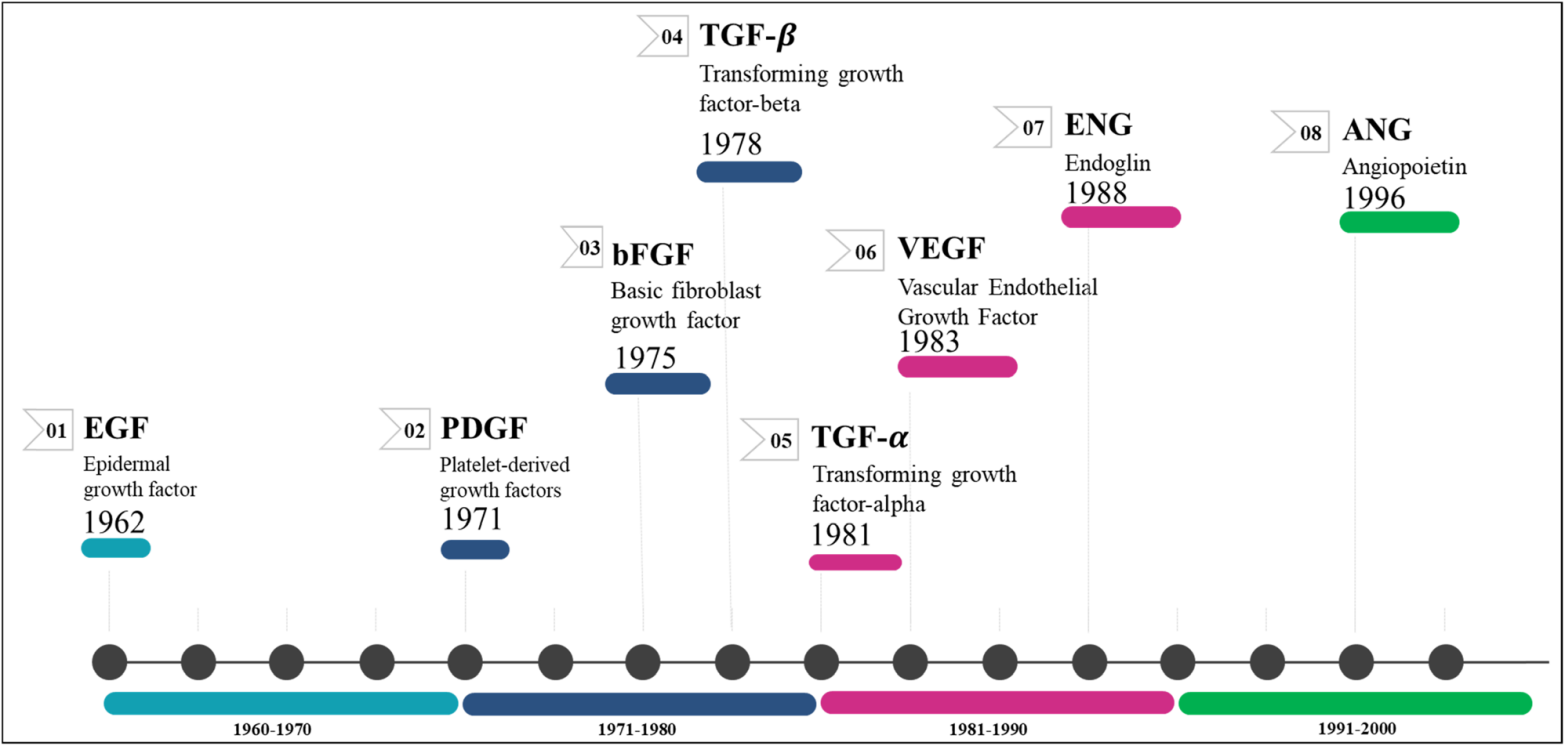
Discovery timeline of various angiogenic targets.

Growth factors (GFs) are protein- or steroid-based hormones that stimulate growth, proliferation, and tissue regeneration.^13^ They attract smooth muscle cells and fibroblasts, which aid in the formation of blood vessels during angiogenesis. GFs also promote the growth and differentiation of endothelial cells. Collectively, these processes are known as sprouting and splitting.^14^ Several angiogenic GFs and their receptors have been discovered so far, with the most notable ones being angiopoietins (ANG), PDGF/R, basic fibroblast growth factor (bFGF/R, FGF/R-2), VEGF/R, TGF/R, insulin-like growth factor/receptor, and EGF/R.^15,16^ Antiangiogenic drugs for cancer treatment primarily target the inhibition of these factor's receptors.^17^Fig. 1 illustrates the chronological sequence of identifying antiangiogenic factors that are crucial targets for fighting against various forms of carcinoma.

The inhibition of angiogenesis has become a viable approach for cancer treatment with the identification of new genes, transcription factors, signalling pathways, and mechanisms linked to tumour angiogenesis.^18,19^ Tumour blood vessels have higher vascular permeability and a better angiogenic potential than normal blood vessels. VEGF is a key component in controlling angiogenesis. As cancer progresses, VEGF has been found to be widely distributed and overexpressed.^20^

### 1.2 Involvement of VEGF and VEGFRs in angiogenesis

The goal of anti-angiogenic therapy is to limit the blood flow to tumour tissue by delivering anti-angiogenic drugs to reduce tumour growth and metastasis.^21^ The VEGF protein was first identified in 1989, and its role in angiogenesis was discovered.^22,23^ Having a weight of 40–45 kD, VEGF is a dimeric protein rich in cysteine that is highly conserved in mammals. It was discovered that VEGF improves the permeability of tumour blood vessels and induces ascites development.^24^ VEGFR-2 is a member of the family of receptor tyrosine kinases (RTKs). When VEGF binds to the protein kinase VEGFR-2, it triggers the production of blood capillaries and mediates the signalling pathway.^25^ Proangiogenic signalling molecules like VEGF and its cognate receptor, VEGFR-2, are essential for angiogenesis and are overexpressed in a lot of cancers.^26^

Blocking VEGFR-2 signalling is therefore seen as one of the most promising ways to prevent tumour-induced angiogenesis.^27,28^ Early antiangiogenic clinical trials focused on VEGF/VEGFR signalling blockade.^29^ The VEGF family is part of a broader category of signalling proteins known as cytokines. The subgroup consists of five proteins, namely VEGF-A, VEGF-B, VEGF-C, VEGF-D, and PlGF (placental growth factor), which have a significant impact on the processes of angiogenesis and lymphangiogenesis.^30^ The most particular of them, VEGFA, was discovered initially and is known to induce angiogenesis.^31^ The tyrosine kinase enzyme in the intracellular receptor domain is activated when VEGF binds to the extracellular domain. This leads to the phosphorylation of tyrosine and the activation of specific intracellular signalling pathways.^32^ VEGFR-1, VEGFR-2, and VEGFR-3 are the three extensively acknowledged receptors for the various members of the VEGF family.^33^ Both VEGFR-1 (Flt-1) and VEGFR-2 (KDR) are mainly expressed by vascular endothelial cells and hematopoietic stem cells, while VEGFR-3 is predominantly located on lymphatic endothelial cells.^34^ Each of these can incorporate specific components related to the VEGF family due to their unique affinity and selectivity. Although there are numerous VEGF/R-related factors that can cause pathological angiogenesis, the primary initiators of lymphangiogenesis are VEGF-C, VEGF-D, and the VEGFR-3 receptor.^35^

Endothelial cells are the primary source of VEGF-A secretion; however, other cell types such as astrocytes, macrophages, dendrocytes, thrombocytes, osteoblasts, lymphocytes, and tumour cells can also release it.^36^ It promotes the recruitment of inflammatory cells, such as macrophages and granulocytes, increases the permeability of blood vessels, prevents cell death, and encourages cell growth.^37^ VEGF-B's contribution to angiogenesis is rather minor. VEGF-B has a crucial role in promoting the survival of smooth muscle cells, neurons, pericytes, cardiac cells, and vascular endothelial cells in normal conditions.^38^ VEGF-C and VEGF-D emerge from the proteolytic cleavage of their precursors. When lymphatic vessels form during embryonic development, VEGF-C expression is high in the heart, thyroid, ovary, placenta, and gut as an adult.^37^ Lymphangiogenesis is stimulated by VEGFR-3 receptors, whereas angiogenesis is less affected. Recent discoveries show that it also binds to the NP-2 receptor, which boosts VEGFR-2 function.^39^ The extracellular portion of the VEGF receptors has seven motifs identical to immunoglobulin, while the intracellular portion contains a tyrosine kinase domain. VEGFR-1 expression in monocytes and macrophages has been demonstrated. It is known that VEGFR-3 is expressed by lymphatic endothelial cells.^40^ The role of VEGFR-3 in embryonic and pathological lymphangiogenesis and its affinity for VEGF-D and VEGF-C are well recognized. Signal pathways like PKC, Ras, and Akt/GDP are responsible for VEGFR-3 activation. Active VEGFR-3 promotes lymphatic endothelial cell proliferation, migration, differentiation, and survival.^41^

## 2. Structure of VEGFR-2

**Fig. 2:**
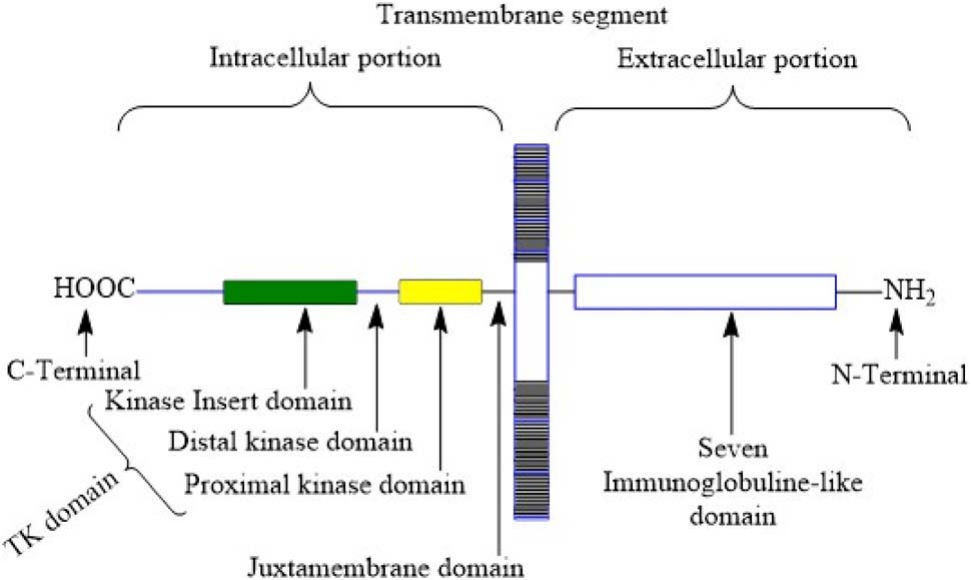
Structure of vascular endothelial growth factor receptor-2.

VEGFR-2 is a 200–230 kDa molecular weight receptor known as FLK-1 as well as KDR.^42^ VEGFR-2 is widely acknowledged as the most significant among all three VEGFRs,^43^ has been extensively studied, and its presence has been firmly confirmed in various types of cancer.^44^ When VEGF attaches to the extracellular domain, it triggers the activation of tyrosine kinase in the intracellular domain ^45^. VEGF's interaction with VEGFR-2 triggers the activation of the PLC/PKC, Ras/Raf/ERK MAPK, and PI3K/Akt pathways. These pathways play a crucial role in regulating both normal and abnormal blood vessel growth.^46,47^ There are 18 N-linked glycosylation sites, 15 phosphorylation sites, and numerous ATP-binding sites and substrate binding sites in human VEGFR-2, which play important roles in VEGFR-2 post-translational modifications, protein folding, protein activation, and cellular attachment and can further regulate the VEGFR-2 function.^48,49^ Human VEGFR-2 encodes 1356 amino acids of the full-length receptor, and the corresponding gene for it is situated at chromosome locus 4q.^50^ It responds better to VEGF-A than it does to VEGF-D or VEGF-C. Tyrosine kinase activity is higher in VEGFR-2 than in VEGFR-1. VEGFR-2 has a tyrosine kinase domain, an external ligand-binding domain, and a transmembrane domain. The goal of activating the receptor is to boost angiogenesis and vascular permeability.^51^ VEGFR-2 levels grow during the development of embryonic blood vessels, angiogenesis, and tumour angiogenesis.^52^Fig. 2 shows the structure of VEGFR-2. The main places where phosphorylation happens are Y951, Y1054, Y1059, Y1175, and Y1214.^53^ Following VEGF binding, the primary auto-phosphorylation sites in human VEGFR-2, Y1175 and Y1214, activate various downstream pathways, including PI3K, p38MAPK, FAK, Src, and Akt, which are usually hyperactivated in several tumours.^54^ The analysis of the binding site reveals that the VEGFR-2 active site consists of four main components: the hinge area, two hydrophobic areas (hydrophobic-I and hydrophobic-II), and the hydrogen bond-rich region.^55,56^

### 2.1 Pathophysiological role of VEGFR-2 in tumour

The VEGF/VEGFR-2 system is greatly involved in abnormal blood vessel formation, seen in conditions like muscular degeneration, diabetic retinopathy, inflammation, and cancer growth.^57^ De-regulation of VEGF/VEGFR-2 implicates directly in various diseases, and dysfunctional VEGFR-2 can cause developmental disorders of the vascular system and hematopoietic system during embryonic development.^58^ The VEGF/VEGFR-2 system is an important regulator of abnormal angiogenesis in cancer and healthy vasculogenesis in the early embryonic and adult stages.^59^ During the early stages of embryonic life, specifically at day 7.5 of gestation, certain cells known as mesodermic hemangioblasts exhibit the expression of VEGFR-2.^60^ The VEGFR-2 expression affects the migration and differentiation of these cells into endothelial cells. Additionally, it also contributes to the formation of vascular islands in the yolk sac, which marks the initiation of vasculogenesis.^61^ Vasculogenesis is the initial stage of embryonic blood circulatory system development, where progenitors of endothelial cells differentiate and assemble to form the basic vascular plexus.^62,63^

VEGF-activated VEGFR-2 triggers the phosphorylation of multiple proteins in the signalling pathways, such as Akt (protein kinase B), mTOR (mammalian target of rapamycin), Erk1/2 (extracellular signal-regulated kinase 1/2), FAK (focal adhesion kinase), and p70S6K (ribosomal protein S6 kinase), thereby facilitating tumour angiogenesis.^64^ These proteins are prime targets of VEGFR inhibitors.^65^

### 2.2 Bio-physiological significance of VEGFR-2

The majority of VEGFR-2 is expressed on blood and lymph vessel endothelial cells, where it stimulates integrins to promote cell motility and inhibit cell death.^66^ When Akt protein kinase is activated, it leads to vasodilation by enabling the production of endothelial nitric oxide synthase (eNOS),^67^ which forms nitric oxide. In addition, the activation of VEGFR-2 stimulates the production of von Willebrand factor (vWF) by endothelial cells.^68^ Since VEGFR-2 has been reported to have the highest degree of proangiogenic action, inhibiting it may have clinical implications.

VEGFR-2 is more active as a tyrosine kinase than VEGFR-1 but has a lower affinity for VEGF-A.^69^ Tumour cells release VEGF, which activates its receptor VEGFR-2, promoting vascular development and supplying oxygen and nourishment into hypoxic parts of tumour tissues.^70^ Multiple inquiries have demonstrated that the VEGF/VEGFR-2 signalling pathway exerts direct control over neuronal development and its function, particularly by promoting increased branching of axons.^71,72^

### 2.3 VEGFR-2 and its involvement in different pathways

**Fig. 3:**
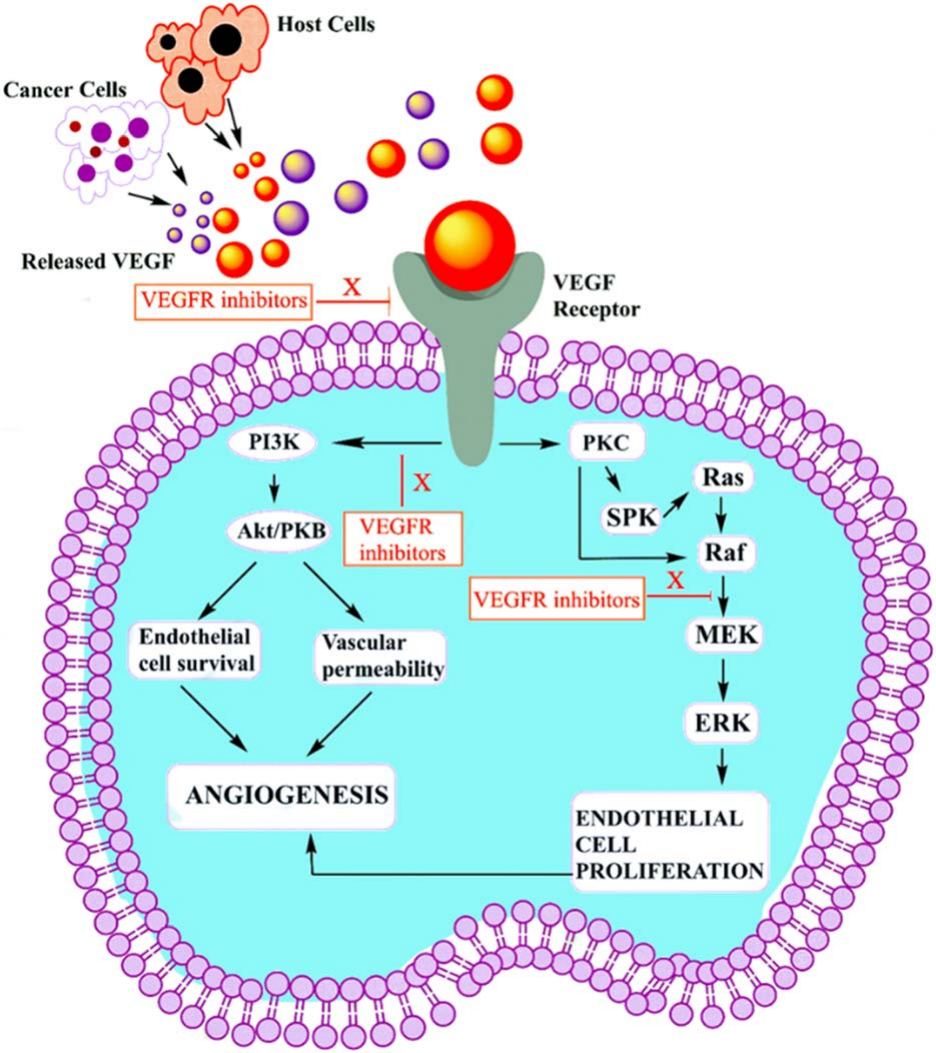
Role of VEGFR-2 inhibitor in tumour cell.

VEGF-A binding to VEGFR-2 triggers the activation of the Ras/Raf/ERK/MAPK, PI3K/Akt, and PLCγ/PKC pathways, which influence angiogenesis in pathophysiological domains.^41^ The PI3K dimer governs angiogenesis, emigration, cell division, and viability with its p110 catalytic domain and p85 response regulator. VEGF-A upregulates PI3K and phosphorylates p85. Phosphatidylinositol-3,4,5-triphosphate accumulates when VEGFR stimulates PI3K, phosphorylating Akt/PKB. Akt/PKB upregulates and suppresses BAD, caspase-9, and proapoptotic proteins.^50^Fig. 3 denotes the inhibitors and their targeting pathways associated with VEGFR.

Upon VEGFR auto-phosphorylation, a T-cell-specific adaptor binds Tyr951 and links to Src. Src kinases form actin stress fibers and may activate PI3K in response to VEGF-A. VEGFR complex formation regulates Ras expression and starts the Raf-1-MEK-ERK signal cascade, which is essential for growth factor-induced cell division.^73^ When VEGF binds to VEGFR-2, some tyrosine residues of VEGFR-2 become autophosphorylated, such as Try801 on JMD, which further mediates the PLCg-PKC pathway and subsequently eNOS-NO or MEK-ERK, and Try951 on KID, mediates the TSAd-Src-PI3K-Akt pathway. Try1054 and Try1059 on TKD2 increase VEGFR-2 kinase activity.^74^

**Fig. 4:**
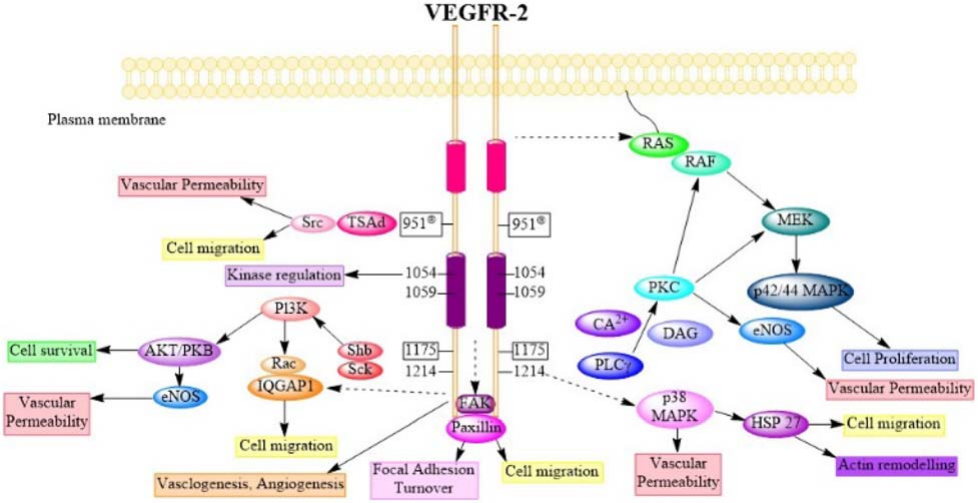
Physiology of VEGFR-2 in normal cells (adapted from Modi, S. J. *et al.* 2019).^51^

Try1175 regulates the PLCg-PKC, SHB-FAK-paxillin, and SHB-PI3K-Rac pathways, and Try1214 mediates cell migration *via* the NCK-p38-MAPK-APK2/3 pathways. These signalling networks regulate angiogenesis, endothelial cell survival, proliferation, and motility, as well as vascular permeability and penetration, through the action of VEGF/VEGFR-2.^75^Fig. 4 (adapted from Modi, S. J. *et al.* 2019) depicts various signal transduction pathways and phosphorylation sites associated with VEGFR-2.^51^

## 3. USFDA-approved drugs targeting VEGFR-2

**Fig. 5:**
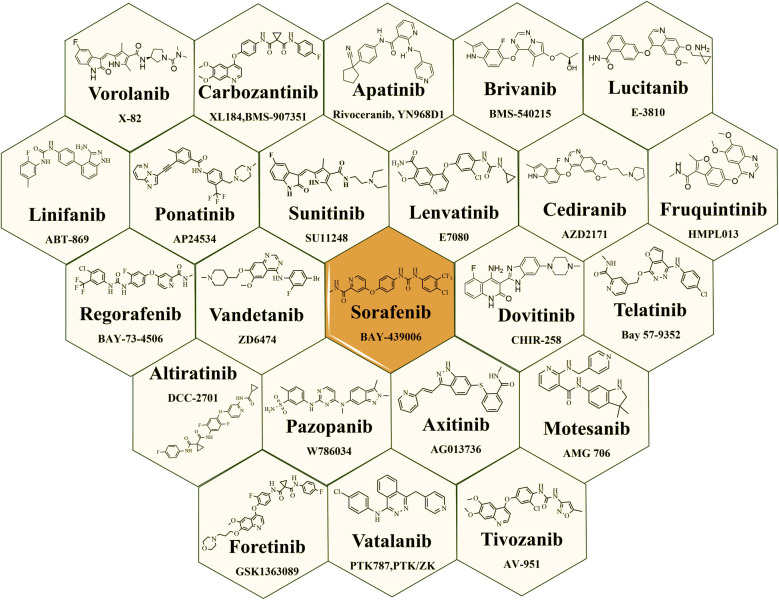
USFDA approved drugs targeting VEGFR-2 as anticancer agents.

Small-molecule as VEGFR-2 inhibitors have indeed emerged as an important class of drugs in cancer treatment over the last few decades. Fig. 5 illustrates the structures of VEGFR-2 inhibitors that received USFDA approval for various types of cancer. The approval of these small-molecule inhibitors marks significant progress in cancer treatment, offering patients additional options and improving outcomes for certain types of cancer.^47,76^ The USFDA approved drugs principally targeting VEGFRs along with specific cancer types are mentioned in the Table 1. However, like all cancer treatments, they come with some moderate to serious side effects and considerations, and their efficacy is dependent on individual patients' cancer profile. While there are many VEGFR-2 inhibitors on the market, there have been documented cases of heart failure and a significant risk of haemorrhages associated with imatinib and other multikinase inhibitors treatments.^100^

**Table 1:** USFDA approved drugs targeting VEGFRs and other RTKs^a^

Sr. no.	USFDA approved drugs	Targets	Cancer type	Ref.
1	Vorolanib	VEGFR-2, PDGFR-β, FLT3, and C-Kit	RCC and lung cancer	77
2	Cabozantinib	MET and VEGFR-2	MTC and RCC	78
3	Apatinib	VEGFR-2	Gastric cancer	79
4	Brivanib	VEGFR-2 and 3, and FGFR-1, 2 and 3	Solid tumor, HCC, and mCRC.	80
5	Lucitanib	FGFR-1 and VEGFR	Solid tumor, SCLC, and mCRC.	81
6	Linifanib	VEGFR-2 and PDGFR-β	Breast cancer, CRC, liver cancer, and NSCLC.	82
7	Ponatinib	VEGFR-2, FGFR, PDGFR, SRC, RET, KIT, and FLT1	CML and Philadelphia chromosome-positive ALL	83
8	Sunitinib	VEGFR, PDGFR, and c-KIT.	mGIST and RCC	84
9	Lenvantinib	VEGFR 1–3, FGFR 1–4, PDGFRα, KIT and RET.	DTC, RCC, and HCC.	85
10	Cediranib	VEGFR-2	Ovarian cancer, Glioblastoma, liver cancer	86
11	Fruquintinib	VEGFR-1, -2 and -3	mCRC	87
12	Regorafenib	VEGFR1–3, TIE2	CRC, GIST, and HCC	88
13	Vandetanib	VEGFR-2 and EGFR	MTC	89
14	Sorafenib	VEGFR-1, -2, -3, PDGFR-β, RET, c-Kit and Fms-like tyrosine kinase 3	Unresectable HCC and aRCC	90
15	Telatinib	VEGFR-2, VEGFR-3, and PDGFR	PHE	91 and 92
16	Altiratinib	VEGFR-2, MET, and TIE2 (TEK)	Glioblastoma	93
17	Pazopanib	VEGFR-1, -2, and -3, PDGFR-β and -α, and c-KIT	Advanced soft-tissue sarcoma and RCC.	94
18	Axitinib	VEGFR-1, -2, and -3	aCRC and Advanced thyroid cancer	95
19	Motesanib	VEGFR-2, c-KIT, and PDGFR.	Breast cancer, Bladder cancer, and thyroid cancer	96
20	Foretinib	VEGFR-2 and -3, c-MET, c-KIT and TIE-2	Ovarian cancer, gastric cancer, and lung cancer	97
21	Vatalanib	VEGFR-1, -2 and 3, EGFR, and FGFR-1	Solid tumor	98
22	Tivozanib	VEGFR-1, -2, and -3	Advanced or metastatic RCC	99

a mMTC: metastatic medullary thyroid cancer; HCC: hepatocellular carcinoma; mCRC: metastatic colorectal cancer; CRC: colorectal cancer; SCLC: small-cell lung cancer; NSCLC: non-small cell lung cancer; CML: chronic myeloid leukemia; ALL: acute lymphoblastic leukemia; mGIST: metastatic gastrointestinal stromal tumor; RCC: renal cell carcinoma; DTC: differentiated thyroid cancer; PHE: pseudomyogenic hemangioendothelioma.

## 4. Small-molecule VEGFR-2 inhibitors

Development of small molecules targeting VEGFR-2 with diverse molecular scaffolds using molecular hybridization and pharmacophore hybridization approaches holds promise for cancer treatment. Utilising different molecular scaffolds and pharmacophoric features, researchers are constantly aiming to discover highly potent, selective, and therapeutically effective lead molecules against various cancers. This strategy allows for the creation of a library of compounds with different chemical structures and properties, offering a wide range of options for optimizing drug candidates for cancer and overcoming resistance mechanisms.

**Fig. 6:**
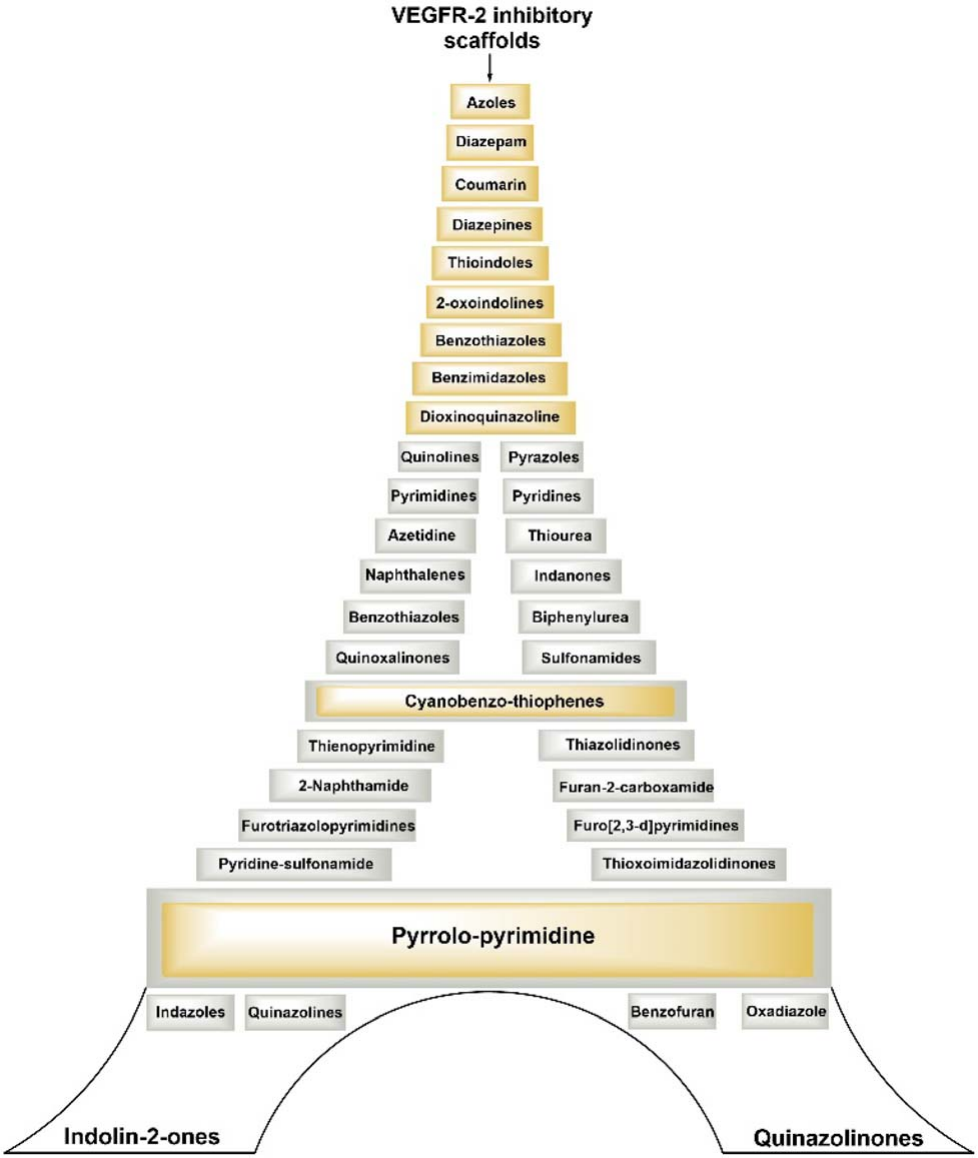
Various scaffolds covered in this review explored for potential VEGFR-2 inhibitory activity.

Fig. 6 illustrates several types of molecular frameworks and heterocyclic components that have been investigated in recent years through the molecular hybridization approach and have demonstrated significant efficacy against VEGFR-2 and different cancer cells. Various cell lines with their codes tested for compounds covered in this review are mentioned in Table 2.

**Table 2:** Various cell lines and their codes tested for compounds covered in this review

Code	Cell lines	Code	Cell lines
MDA-MB-231	Human breast cancer cell line (MDA-MB-468, MDA-MB-435, MCF7, T-47D)	UO-31, CAKI-1, 786-O	Human renal cancer cell lines
DU-145	Human prostate cancer cell line	MRC5	Human normal lung cell line
HCT-116, HT-29	Human colorectal cancer cell line (HCT-8, SW-620, SW-480, H460)	U87MG	Human glioblastoma cell line
HepG2	Hepatocellular cancer cell line	HT-29	Human colon cancer cell lines
PC-3	Human prostate carcinoma	HEK-293	Normal human embryonic kidney cells
K562	Chronic myelocytic leukemia cell lines	MKN-45, SNU-16, MKN-74	Human gastric cancer cell line
HL60	Human acute lymphoblastic leukaemia cell line	A549/ATCC, H1299, H3255, H1975, HCC-78, H460, HCC827	Non-small cell lung cancer cell line
A498, ACHN	Human renal cancer cell line	RPE1	Human retinal pigment epithelial cell line
A375	Human melanoma cancer cell line	A431	Human epidermoid carcinoma cell line
BxPC-3	Human pancreatic cancer cell lines	Hu02	Human dermal fibroblasts cells
HeLa	Human cervical cancer cell line	U251	Human glioblastoma cell line
SNU-5, BGC-823	Human gastric adenocarcinoma cell line	SMMC7721 MHCC97H	Human hepatocellular carcinoma cell line
HEK293, 293T	Human embryonic kidney cell line	HBE	Human bronchial epithelial cells
Huh7	Human hepatoma cell line	HL-60 (TB)	Human leukaemia cell lines
SGC-7901	Human gastric adenocarcinoma	LO2	Human fetal hepatocyte cell line
U251	Brain tumour cell line	HGC-27	Human gastric lymph node cancer cell line
CACO-2	Human colon adenocarcinoma	NCI-H522	Human lung adenocarcinoma
HUVEC	Human umbilical vein endothelial cells	RPMI-8226	Human myeloma cell line
A549	Human lung cancer cell line	VERO	Monkey kidney epithelial line
SNB-75	Human brain tumour cell line	MCF-10F	Non-tumourigenic epithelial cell line
KM12	Human colon cancer cell line	MRC-5	Human Foetal lung fibroblast cells
HOP-92	Human lung large cell carcinoma	M-NFS-60	Bipotential murine hemopoietic cell line
BJ	Human neonatal normal foreskin fibroblasts	Hep3B	Human hepatoma cell line
PANC-1	Human pancreatic epithelial cell line	THLE2	Human normal liver cell line
MCV-7	Merkel cell polyomavirus		

**Fig. 7:**
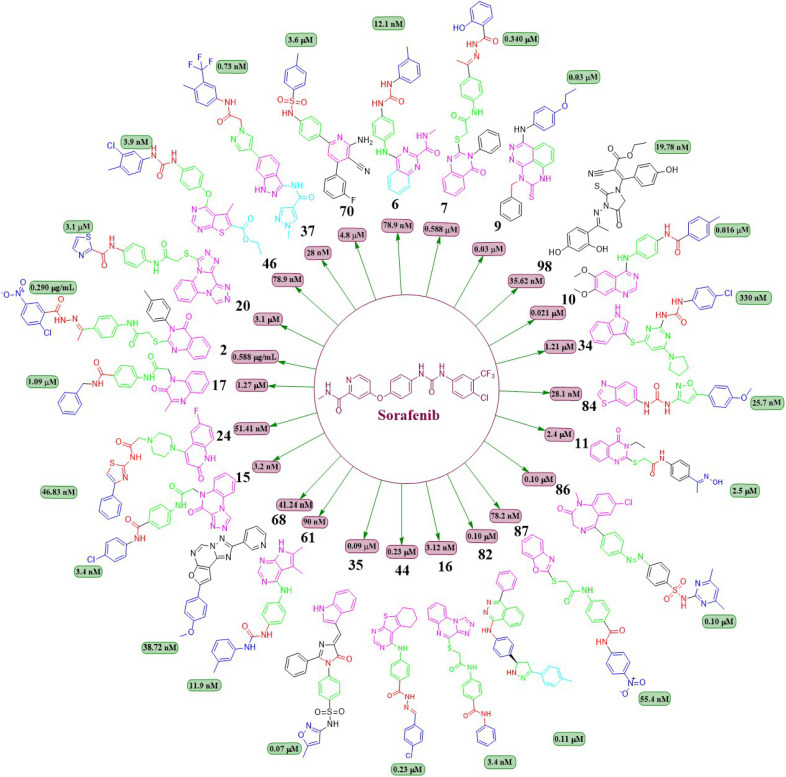
Compounds that displayed comparable inhibitory activity with sorafenib against VEGFR-2*. *Brown-coloured box indicates VEGFR-2 activity corresponds to sorafenib, and green-coloured box indicates activity corresponds to respective compounds using sorafenib as the reference standard in the bioassay. Color code description: 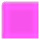 heteroaromatic system; 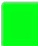 central aromatic ring; 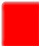 HBD–HBA; 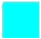 solvent-accessible region; 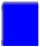 terminal hydrophobic region.

**Fig. 8:**
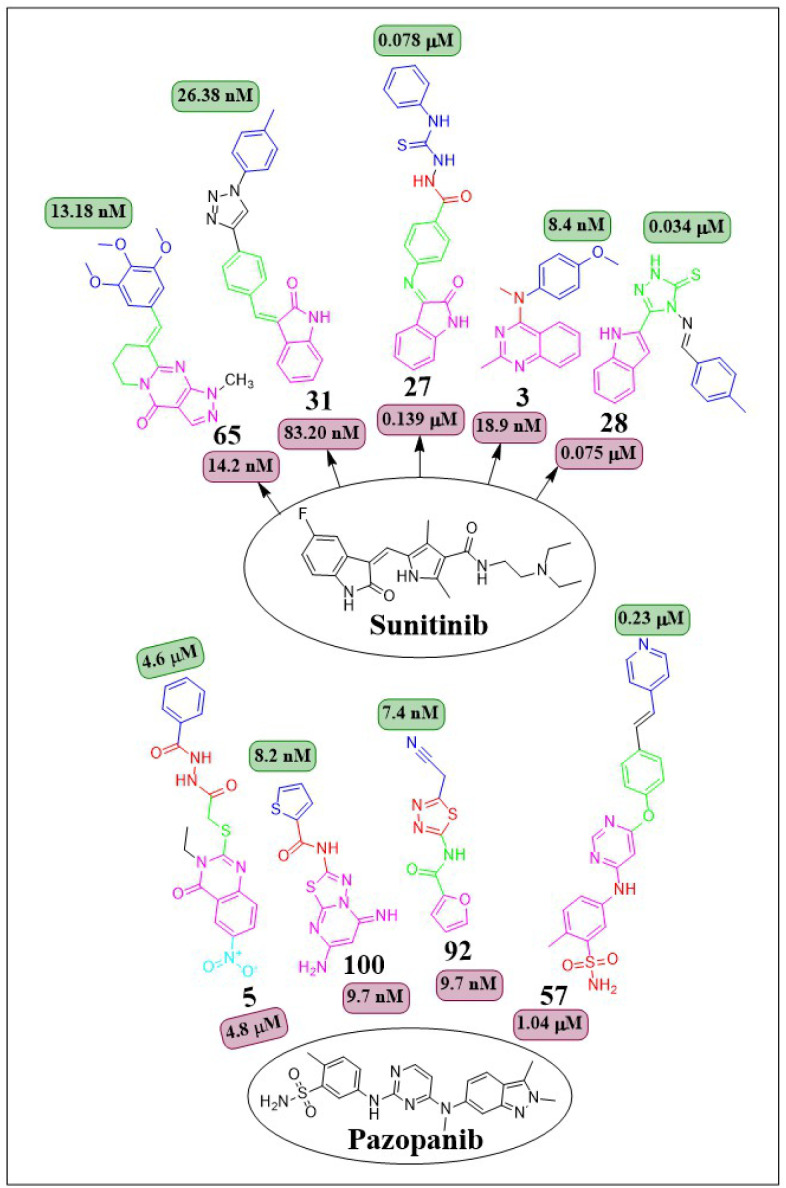
Compounds that displayed comparable inhibitory activity against VEGFR-2 using sunitinib or pazopanib as reference standard*. *Brown-coloured box indicates VEGFR-2 activity corresponds to sunitinib and pazopanib, while green-coloured box indicates activity corresponds to respective compounds using sunitinib and pazopanib as reference standard in the bioassays, respectively.

This review focuses on the design, synthesis, and structure–activity relationship of small compounds that have shown improved anticancer attributes in recent years. This article covers the development of compounds and their subsequent testing against VEGFR-2 to suppress the process of angiogenesis. Fig. 7 and 8 displays the structures of potent compounds against VEGFR-2 from various published reports that outperformed the reference standard, sorafenib, in terms of bioactivity results. Fig. 7 and 8 also displays a comparison of the IC_50_ values of the most potent compounds in the respective series and the IC_50_ values of the reference standard sorafenib obtained under similar bioassay conditions.

### 4.1 Quinazoline analogues

Ibrahim H. Eissa *et al.* (2021) investigated the features of quinazolin-4 (3H)-ones and evaluated them against the HepG-2 cell line of hepatocellular carcinoma. Compound 1 demonstrated the highest potency (IC_50_ = 4.33 ± 0.2 μg mL^−1^) against the VEGFR-2 kinase. It exhibited greater activity than doxorubicin (IC_50_ = 4.50 ± 0.2 μg mL^−1^) and was 78% as powerful as sorafenib (IC_50_ = 3.40 ± 0.25 μg mL^−1^). Compound 1 demonstrated exceptional binding energy of −59.62 kcal mol^−1^ in the active region of VEGFR-2.^101^

In a study conducted by El-Adl and co-workers (2021), some quinazolin-4(3*H*)-ones were modified and tested to determine their ability to inhibit VEGFR-2. The IC_50_ value for the most effective compound was found to be 0.290 ± 0.05 μg mL^−1^, which was greater than the reference standard sorafenib's IC_50_ value (0.588 ± 0.04 μg mL^−1^). Compound 2, which includes a 2-chloro-5-nitrophenyl group, has been identified as the most potent member. The activity is enhanced by incorporating electron-withdrawing groups on the 3rd position of the phenyl ring in the terminal hydrophobic region. The compound exhibited approximately 1.96-, 5.73-, and 4.39-fold higher activity towards MCF-7, HCT-116, and HepG2 cells, respectively, compared to sorafenib. The computed binding free energy of compound 2 at the active site of VEGFR-2 was found to be −58.02 kcal mol^−1^.^102^

In their study, Shruti Choudhary *et al.* (2021) afforded substituted quinazolines and tested their effectiveness against EGFR, Flk-1 (VEGFR-2), and PDGFR-β kinases. They discovered that compounds 3 and 4 exhibited greater potency, with kinase activity levels of 8.4 ± 2.2 nM and 9.3 ± 3.9 nM, respectively, compared to sunitinib, which had an activity of 18.9 ± 2.7 nM at VEGFR-2. Remarkably, it is worth noting that compounds 3 and 4 outperformed in the CAM experiment with IC_50_ values of 2.8 ± 1.1 μM and 3.1 ± 1.3 μM respectively, as compared to the reference standard erlotinib (IC_50_ = 3.1 ± 1.3 μM). The binding affinity of the complex between VEGFR-2 and compound 3 was −10.71 kcal mol^−1^, closer to that of axitinib (−13.25 kcal mol^−1^). The quinazoline structure binds to Phe1047 and forms a cation–pi interaction with the sidechain of Lys868. The 4th-position substituent of aniline (compound 3) forms interactions with Val848, Cys919, Leu1035, and Phe1047. The lack of methyl group on the 2nd position of quinazoline leads to a significant decrease in VEGFR-2 activity.^103^

A group of quinazolin-4(3*H*)-ones was developed and tested against VEGFR-2 by Abdallah E. Abdallah *et al.* (2021). The most effective compound 5, shown IC_50_ of 4.6 ± 0.06 μM, which was more significant than pazopanib's IC_50_ of 4.8 ± 0.07 μM. Also, it showed IC_50_ values of 30.85 ± 2.3 μg mL^−1^ against MCF cell lines, 17.23 ± 1.5 μg mL^−1^ against HepG2, and 26.10 ± 2.2 μg mL^−1^ against PC3. Results from docking investigations showed that compound 5 could bind to the active site of VEGFR-2 with a score of −7.42 kcal mol^−1^, effectively forming three crucial hydrogen bonds with the amino acid residues Glu885, Asp1046, and Cys919 in the appropriate way.^104^

In a recent study conducted by Soha R. Abd El Hadi *et al.* (2020), a series of urea-based quinazoline derivatives were meticulously designed and synthesized. These derivatives were then thoroughly evaluated for their VEGFR-2 inhibition profile. Compound 6 exhibited remarkable potency, with an IC_50_ value of 12.1 nM, surpassing that of sorafenib (IC_50_ = 78.9 nM). The NCI 60 cell line screen program was used to investigate most of the newly synthesized compounds. The docking study of the synthesized compounds showed that compound 6 has binding modes similar to the lead compound at the active site of VEGFR-2. The presence of a terminal phenyl ring is crucial for the activity. Introducing an amide or ester moiety at position 2 enhances the activity by creating an additional hydrogen bond with Cys919. Ester derivatives show higher activity when substituted with a 3-F group compared to the unsubstituted compound. The alignment of target compound 6 with sorafenib as co-crystallized ligand (PDB ID: 4ASD) showed a significant alignment with score of 0.790, suggesting a strong resemblance in their molecular fields.^105^

**Fig. 9:**
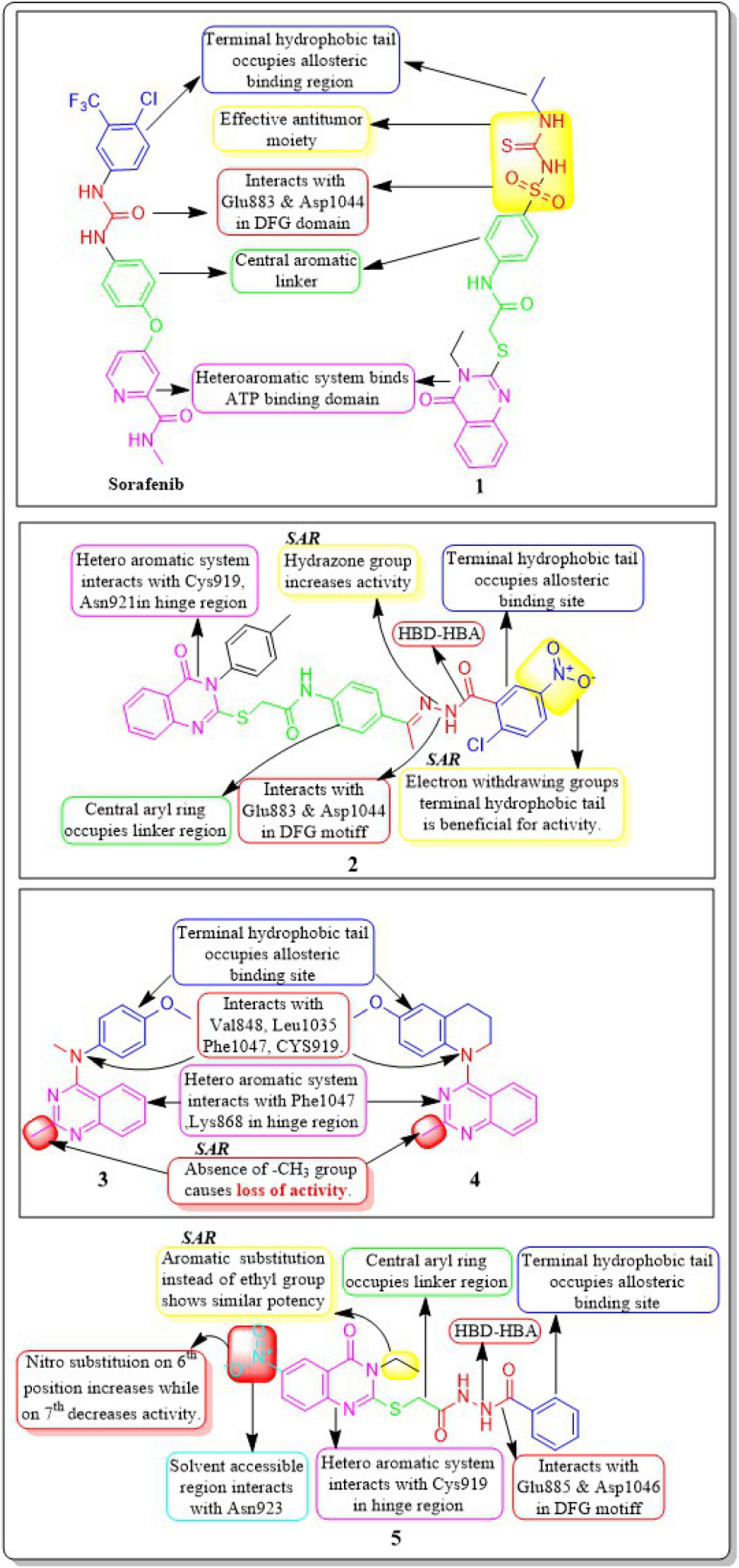
Pharmacophoric features and SAR of compounds 1 to 5.

Ibrahim H. Eissa *et al.* (2020) conducted a study where they developed and tested a new series of quinazolin-4(3*H*)-ones for their potential antiproliferative outcomes. Compound 7 demonstrated a greater inhibitory action on VEGFR-2 (IC_50_ = 0.340 ± 0.04 μM) compared to sorafenib (IC_50_ = 0.588 ± 0.06 μM), which served as the reference drug. Upon comparing the cytotoxic activity of compound 7 against HepG-2, MCF-7, and HCT-116 cell lines with that of doxorubicin and sorafenib, it was discovered to be quite promising. According to docking studies, the novel compounds were found to have a strong inhibitory effect on VEGFR-2. The inhibition was attributed to their ability to make hydrophobic contact with the receptor's hydrophobic pocket and bind to the important residues Glu883 and Asp1044 in the active region. Compound 7 was successfully docked into the active site of the VEGFR-2 kinase enzyme, exhibiting a docking energy score of −56.21 kcal mol^−1^, which outperformed Sorafenib's score of −52.20 kcal mol^−1^. Substituting the hydroxyl group in position 7 for the phenyl group connected to the hydrazone moiety resulted in improved biological activity and binding affinity with VEGFR-2 compared to members that had substituted the chloro group (Fig. 9).^106^

Haoru Fan *et al.* (2019) developed and assessed dioxinoquinazoline derivatives as inhibitors of VEGFR-2. During the anti-tumour animal trials conducted on mice, the tumour exhibited a significant reduction, with a tumour growth inhibition (TGI) rate of 133.0%. After six days of administering a dose of 8, it was observed that it exhibited potent inhibitory action against VEGFR-2 (IC_50_ = 2.4 nM) and displayed remarkable antiproliferative effects on HUVEC cells (IC_50_ = 1.2 nM). Compound 8 exhibited significant action against the HEK293, LO2, and SMMC7721 cell lines, with respective concentrations of 7541 nM, 3855 nM, and 375.5 nM. The strong inhibitory impact can be attributed to the similar bonding sites between compound 8 and lenvatinib in the active site of VEGFR-2.^107^

**Fig. 10:**
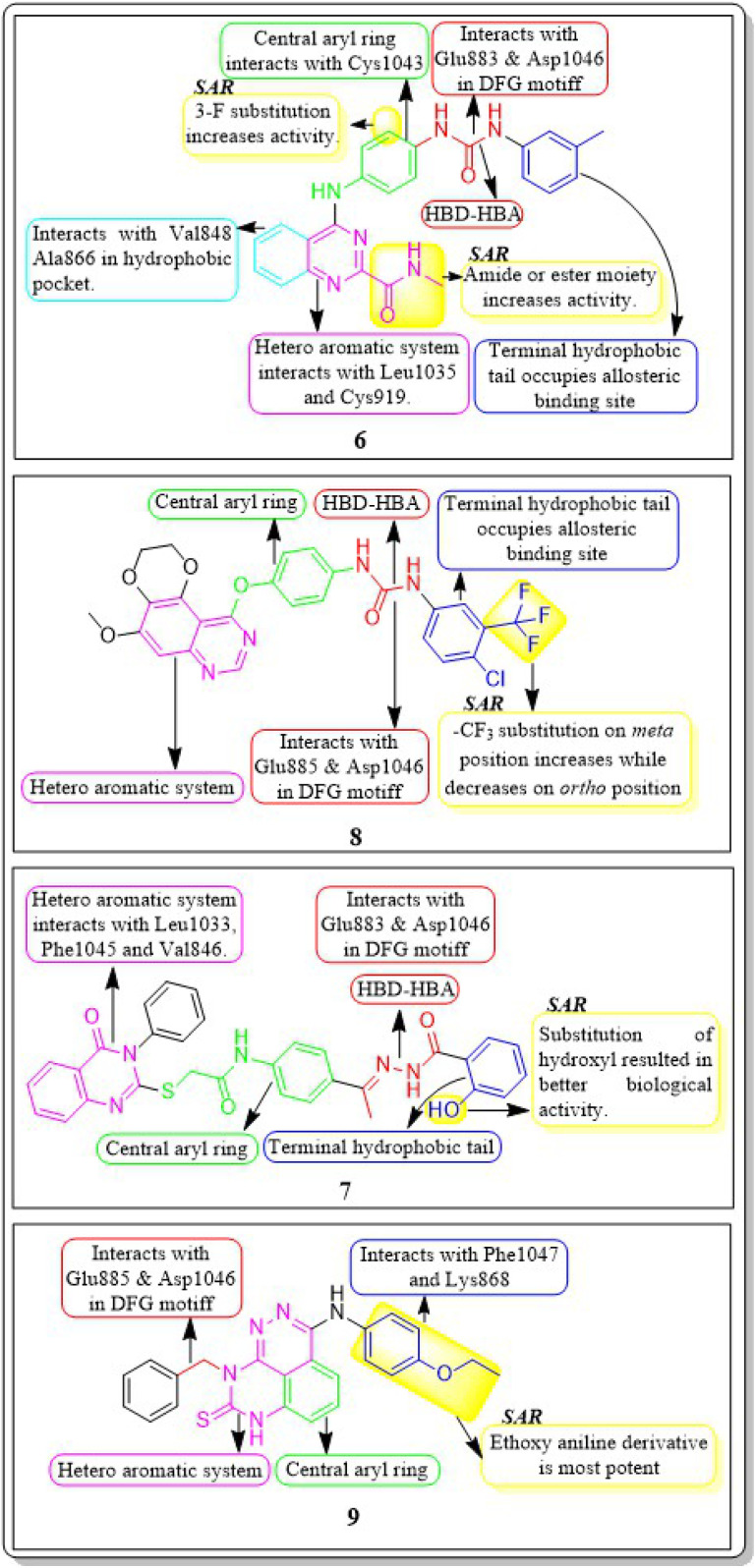
Pharmacophoric features and SAR of compounds 6 to 9.

In a recent study, Marwa El-Gazzar *et al.* (2019) synthesized and bio-evaluated new variations of pyridazino[3,4,5-*de*]quinazoline. Out of all, compound 9 showed greater anticancer activity against the HepG2 cell line and displayed excellent inhibition towards VEGF-2 (IC_50_ = 0.22 μM and 0.03 μM) compared to the reference standard sorafenib (IC_50_ = 1.06 μM and 0.03 μM, respectively). The synthesized compound achieved a docking score of −15.21 kcal mol^−1^ at the active binding site of VEGFR-2 (Fig. 10).^108^

Ru Wang *et al.* (2021) designed, synthesized, and analysed 6,7-dimethoxy-4-anilinoquinazoline analogues containing a diarylamide group. Compound 10 had the highest level of inhibition for VEGFR-2 (IC_50_ value of 0.016 ± 0.002 μM). This is superior to the reference standard, sorafenib, which had an IC_50_ value of 0.021 μM. Compound 10 also exhibited an antiproliferative impact against Hep-G2 and MCF-7, with concentrations of 7.5 ± 0.5 μM and 13 ± 2.8 μM, respectively. The positional influence of the functional group on the terminal phenyl is as follows: *meta* is greater than *ortho*, which is greater than *para*.^109^

A new set of quinazolin-4(3*H*)-one analogues was developed by Hazem A. Mahdy *et al.* (2021). Compound 11 has shown superior potency in the biological evaluation when compared to the other compounds. Compound 11 demonstrated impressive activity towards VEGFR-2 (IC_50_ = 2.5 ± 0.04 μM). The level of activity observed was nearly on par with sorafenib, which exhibited an IC_50_ value of 2.4 ± 0.05 μM. Compound 11 has shown remarkable activity against HepG-2 and HCT-116 cells, exhibiting IC_50_ values of 3.97 ± 0.2 μg mL^−1^ and 4.83 ± 0.2 μg mL^−1^, respectively. By comparison, its activity was 1.13 and 1.08 times greater than that of doxorubicin (IC_50_ = 4.50 ± 0.2 μg mL^−1^ and 5.23 ± 0.2 μg mL^−1^, respectively) and 1.17 and 0.91 times greater than that of sorafenib (IC_50_ = 3.40 ± 0.2 μg mL^−1^ and 5.30 ± 0.2 μg mL^−1^, respectively). Surprisingly, compound 11 exhibited a lower binding free energy (Δ*G*) of −59.90 kcal mol^−1^ compared to the reference drug's −52.20 kcal mol^−1^ when tested against the VEGFR-2 active site.^110^

2-Thioxobenzo[*g*]quinazoline derivatives were synthesized and assessed by Hatem A. Abuelizz *et al.* (2020). Compound 12 was the most effective out of all of them, exhibiting substantial action against VEGFR-2 at an IC_50_ of 44.4 ± 2.6 nM. It was unexpectedly important to note that compound 12 outperformed doxorubicin (IC_50_ = 28.5 ± 1.9 μM and 10.3 ± 0.8 μM, respectively) in terms of activity towards the MCF-7 (IC_50_ = 9.4 ± 0.7 μM) and HepG2 (IC_50_ = 26.0 ± 2.5 μM) cell lines. For 12 and sorafenib, the estimated free energies of binding were −9.669 kcal mol^−1^ and −11.01 kcal mol^−1^, respectively.^111^

**Fig. 11:**
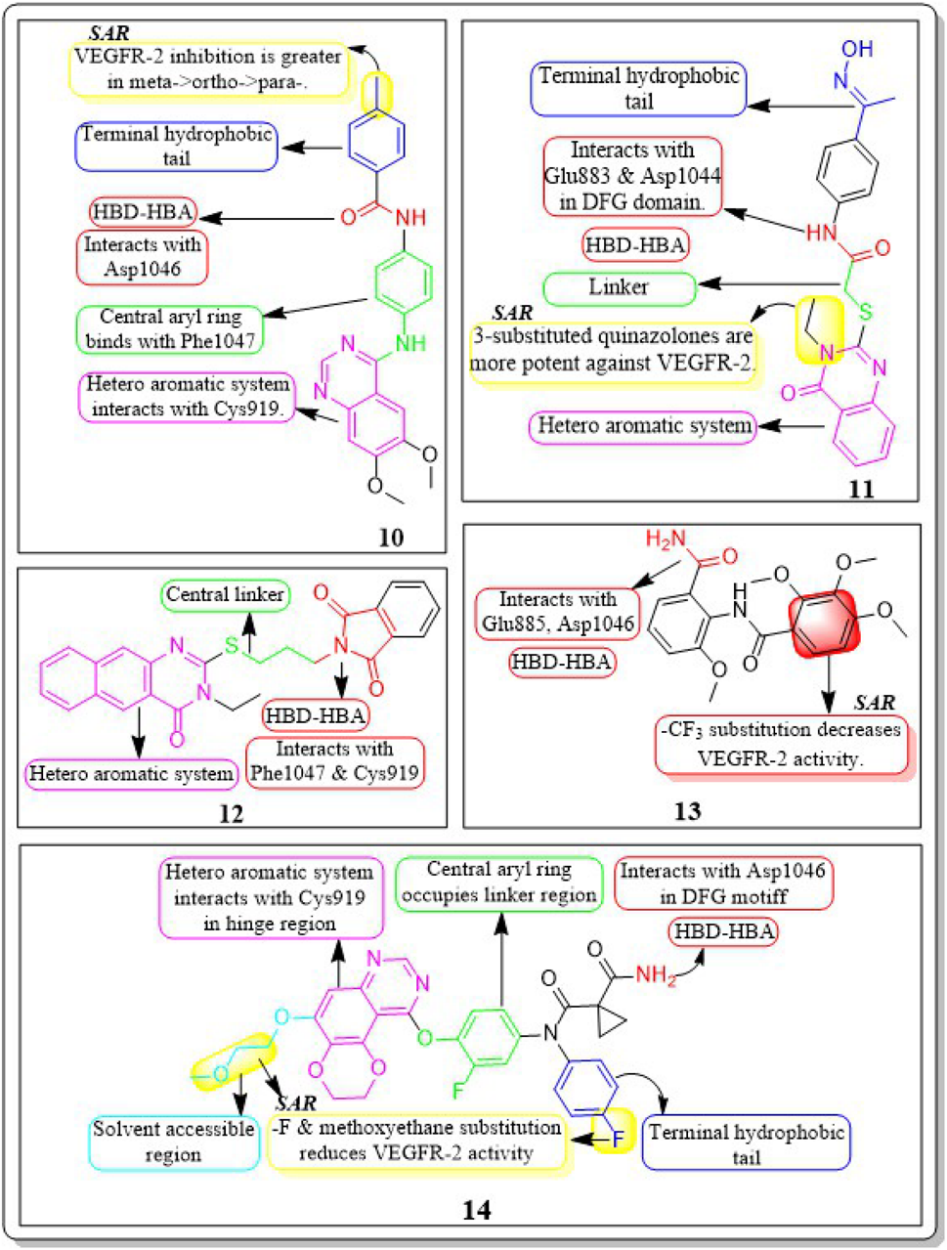
Pharmacophoric features and SAR of compounds 10 to 14.

Abdulmalik S. Altamimi *et al.* (2021) conducted a study where they synthesized and evaluated a novel set of 8-methoxy-2-trimethoxyphenyl-3-substituted quinazoline-4(3)-one compounds. The anticancer properties of compounds were determined by testing them against three different cell lines: MDA, A549, and HeLa. The researchers compared the results of these tests to those of docetaxel, which served as the reference drug. Compound 13 exhibited the highest effectiveness against cancer and was identified as a VEGFR-2 inhibitor with an IC_50_ value of 106 nM, in comparison to docetaxel, which had an IC_50_ value of 56.1 nM. Compound 13 exhibited potent activity towards the HeLa cell line, with an IC_50_ value of 2.8 μM, which was significantly greater than that of docetaxel (IC_50_ = 9.65 μM). Compound 13 exhibited a comparable cytotoxic effect against the MDA cell line (IC_50_ = 0.79 μM) compared to docetaxel (IC_50_ = 3.98 μM). Compound 13 binds to the ATP-binding pocket of VEGFR-2 with a docking energy of −7.3 kcal mol^−1^ (Fig. 10 and 11).^112^

Dengshuai Wei *et al.* (2019) developed a series of [1,4]dioxino[2,3-*f*]quinazoline derivatives. Compound 14 has the highest inhibition for VEGFR-2 (IC_50_ = 4.8 nM). Compound 14 inhibited HEK293 and LO2 cell lines (IC_50_ = 11.9 μM, 12.2 μM). Following additional analysis, compound 14 inhibited MHCC97H and HUVEC cell lines more effectively than cabozantinib (IC_50_ = 25.0 nM, 2.7 nM). Compound 14 showed a TGI (%) of approximately 120.4%. The –F substitution and methoxyethane moiety improve VEGFR-2 activity. Methyl substitution in the heteroaromatic system boosts both the activity and cytotoxicity of the molecule. Compound 14 established an H-bond with the Asp1046 residue at the active site of VEGFR-2.^113^

### 4.2 Quinoxaline derivatives

Nawaf A. Alsaif *et al.* (2021) constructed a novel class of [1,2,4]triazolo[4,3-*a*]quinoxalin-4(5H)-ones and tested their ability to inhibit the growth of two specific cancer cell lines, MCF-7 and HepG2. Compound 15 exhibited a significant inhibitory effect against VEGFR-2 (IC_50_ = 3.4 ± 0.3 nM) compared to sorafenib (3.2 ± 0.1 nM). The Δ*G* (binding free energy) of the synthesised compound 15 against VEGFR-2 was identified to be −21.59 kcal mol^−1^, while the reference sorafenib had a Δ*G* of −21.17 kcal mol^−1^. Upon molecular docking visualisation, it was shown that compound 15 interacts with the binding site of VEGFR in a manner that is analogous to that of sorafenib (Fig. 11).^114^

In their recent study, Alsaif *et al.* (2021) synthesised a new set of [1,2,4]triazolo[4,3-*a*]quinoxaline derivatives and assessed their viability as VEGFR-2 inhibitors. Compound 16 demonstrated exceptional potency, as evidenced by its IC_50_ value of 3.4 nM, which was closer to sorafenib's IC_50_ of 3.12 nM. By substituting a –Me group on the *meta*- or *ortho*-position on the phenyl group, the inhibitory effect was drastically diminished. Compound 16 demonstrated the most pronounced inhibitory effects on cell proliferation in the MCF-7 cell line, as evidenced by its IC_50_ values of 8.2 M, which were considerably lower than those of sorafenib (IC_50_ = 3.51 M).

A double staining experiment was performed utilising annexin V and propidium iodide (PI) to assess whether the inhibition of VEGFR-2 induced apoptosis in HepG2 cells. The experimental procedure involved subjecting HepG2 cells to compound 16 at a 5.4 μM concentration for a period of 24 hours. Compound 16 demonstrated a marginally reduced energy binding of −21.94 kcal mol^−1^ in comparison to sorafenib's energy binding of −22.10 kcal mol^−1^.^115^

In a recent study, Khaled El-Adl *et al.* (2021) conducted a thorough study of a novel set of quinoxaline-2(1*H*)-one derivatives. These compounds were specifically designed to investigate their potential to inhibit cell proliferation in three different cancer cell lines (HCT-116, HepG-2, and MCF-7). Compounds 17 and 18 demonstrated strong inhibition of VEGFR-2 with IC_50_ values of 1.09 μM and 1.19 μM, respectively, which were significant than that of sorafenib (IC_50_ = 1.27 μM). Typically, compounds 17 and 18, with their hydrophobic and electron-withdrawing distal benzyl moiety, exhibited greater activity against the three cancer cell lines compared to compounds with hydrophobic and electron-donating propyl, cyclohexyl, dimethyl, and ethyl moieties. The docking binding free energies of the synthesised compounds 17 and 18 against the VEGFR-2 active site have been estimated to be −19.34 kcal mol^−1^ and −19.65 kcal mol^−1^, respectively.^116^

**Fig. 12:**
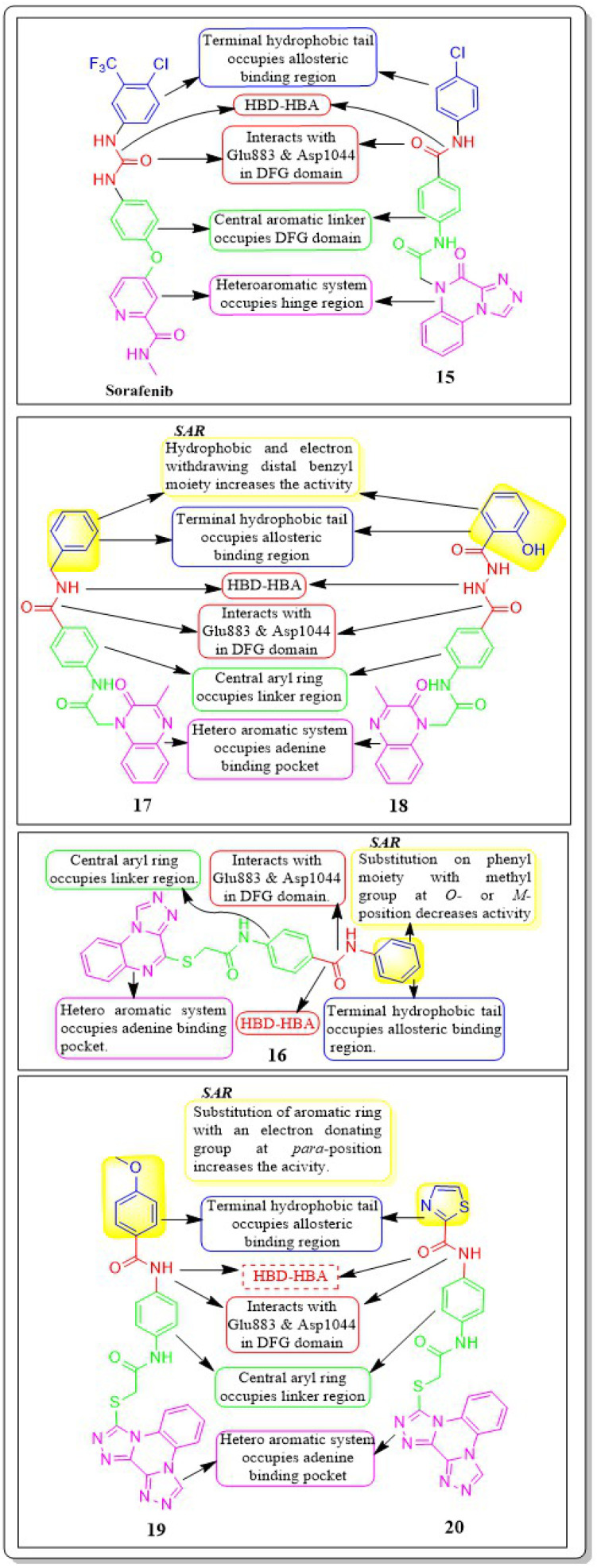
Pharmacophoric features and SAR of compounds 15 to 20.

Mohammed M. Alanazi *et al.* (2021) produced a novel class of bis([1,2,4]triazolo)[4,3-*a*:3′,4′-*c*]quinoxaline analogues and tested them against two human cancer cell lines, HepG-2 and MCF-7. Compounds 19 and 20 showed promising VEGFR-2 inhibitory activity, with IC_50_ values of 3.2 μM and 3.1 μM, respectively, compared to the reference drug sorafenib, which had an IC_50_ value of 3.1 μM. The synthesised compounds 19 and 20 have greater binding free energies (Δ*G*) than the reference candidate sorafenib (−26.50 kcal mol^−1^) against the active site of VEGFR-2 (Fig. 12).^117^

### 4.3 Quinoline analogues

In their study, Xinyu Li *et al.* (2018) developed an assortment of 3-aryl-quinolin compounds specifically designed to interact with VEGFR-2. Out of all the compounds tested, 21 exhibited impressive activity at a value of 86 nM against VEGFR-2. Cell proliferation was effectively inhibited by Compound 21 in HUVEC, MCF-7, and Ishikawa cell lines, with IC_50_ values of 7.4 μM, 1.8 μM, and 1.8 μM, respectively. The inhibitory effects on the growth of MCF-7 breast cancer cells and the potential to prevent angiogenesis in laboratory settings are primarily associated with the presence of 2-methylpiperazine at the side chain terminal.^118^

Yuqin Yao *et al.* (2020) developed and analysed a new group of quinoline-thiourea motiff. Remarkably, compound 22 exhibited superior activity (IC_50_ = 7.0 ± 2.0 nmol L^−1^) compared to the reference standard Nintedanib (IC_50_ = 8.8 ± 3.6 nmol L^−1^) against VEGFR-2.^119^ In addition, compound 22 significantly decreased HUVEC growth, with an IC_50_ value of 71.0 ± 5.0 nmol L^−1^. The quinoline ring and thiourea-containing compound 22 established hydrogen binding with Cys919 (hinge region) and Glu885 on VEGFR-2. This interaction hindered the attachment of ATP to the ATP-binding site of VEGFR-2.^119^

**Fig. 13:**
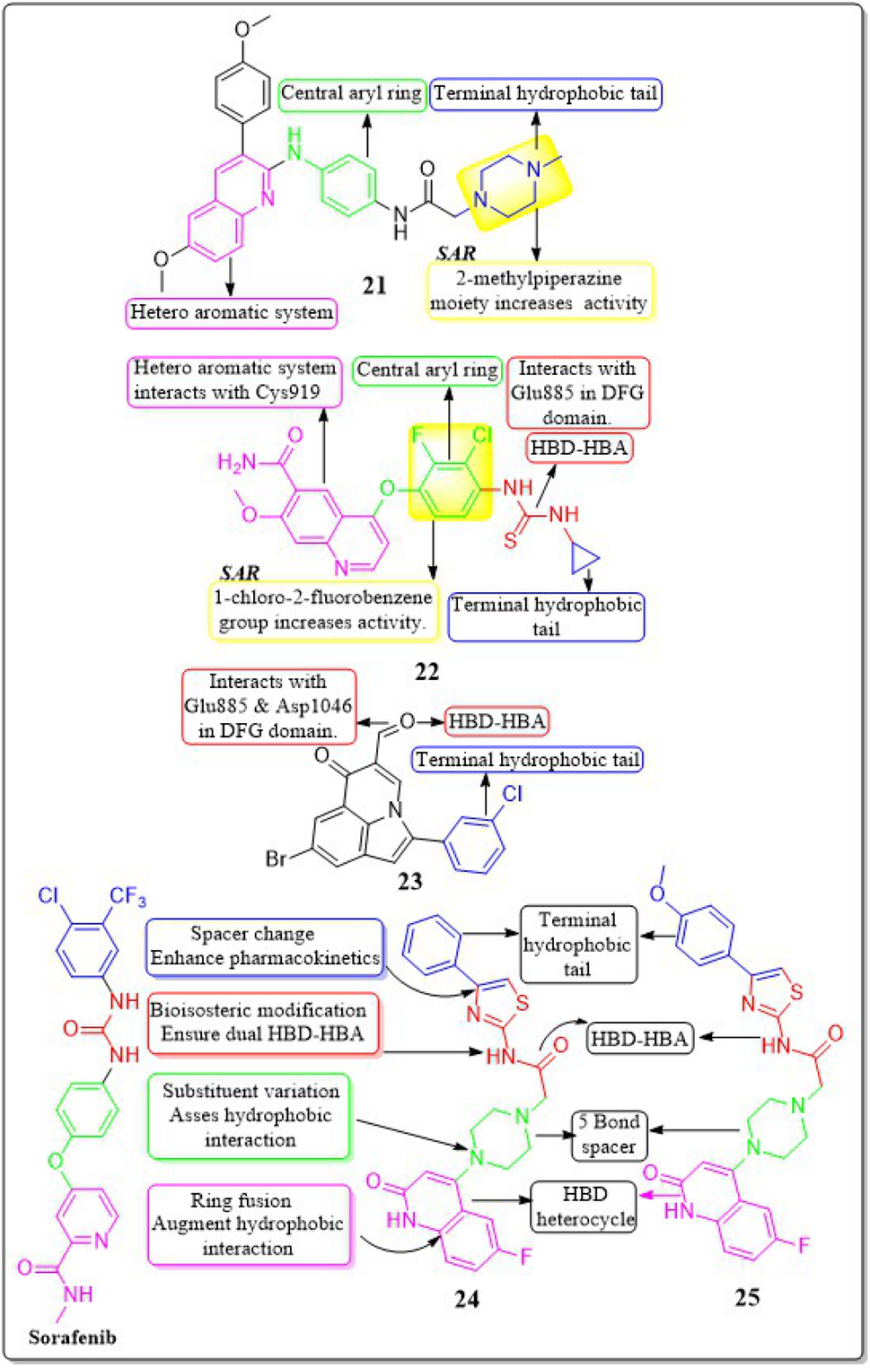
Pharmacophoric features and SAR of compounds 21 to 25.

In a recent study, Malose J. Mphahlele *et al.* (2020) successfully synthesised a series of pyrroloquinoline-5-carbaldehydes. In comparison to the reference standard camptothecin (IC_50_ = 10.37 μM), compound 23 exhibited the most potent inhibitory action (IC_50_ = 0.13 μM) against VEGFR-2 kinase. Compound 23 demonstrated significant efficacy against MCF-7, MDA-MB-231, and HEK293-T cell lines, with IC_50_ values of 11.33 ± 0.53, 9.64 ± 0.01, and >100, respectively. For increased activity, it is believed that the carbaldehyde group of these polycyclic compounds forms hydrogen bonds with the catalytic region of VEGFR-2, acting as a hydrogen acceptor. The free energy of binding for compound 23 has been identified to be −9.45 kcal mol^−1^. The antiproliferative activity of a 4-fluorophenyl group in the 2-position of the heterocyclic ring was found to be lower compared to when the halogen atom was on the *meta* position of the phenyl group (Fig. 13).^120^

### 4.4 Oxoquinolone derivatives

In a recent study, Abdelfattah Hassan *et al.* (2021) developed a novel series of 2-(4-(2-oxo-1,2-dihydroquinolin-4-yl)piperazin-1-yl)-*N*-(4-phenylthiazol-2-yl)acetamides.^121^ These analogues were then tested for their potential anticancer properties. Compounds 24 and 25 exhibited inhibitory activity with IC_50_ values of 46.83 ± 2.4 nM and 51.09 ± 2.6 nM, respectively. These values are comparable to the inhibitory activity of sorafenib, which has an IC_50_ value of 51.41 ± 2.3 nM. Substituting the nitrogen atom of the quinoline nucleus results in a decline in potency, whereas the presence of a fluoro substitution is crucial for maintaining activity. Compounds 24 and 25 exhibited binding interactions at the active site of VEGFR that were comparable to those of the standard drug sorafenib. Compound 24 revealed an interesting interaction with Cys919 in the hinge region of the enzyme. Specifically, quinoline-2(1*H*)-one formed a dual hydrogen bond with Cys919 through its nitrogen and oxygen atoms, with distances of 3.5 Å and 2.52 Å, respectively. Compound 24 formed hydrogen binding with Phe918 (2.87 Å) and Asp1046 (2.99 Å). Both compounds 24 and 25 replaced the central phenyl group of sorafenib with the piperazine moiety, effectively filling the gap between the enzyme's hinge region and gate area. Compound 24's 4-phenylthiazole part fit snugly into the enzyme's hydrophobic allosteric site, which was made up of the side chains of Ile888, Leu892, Val898, Val899, and Cys1024. This site was similar to the one occupied by Sorafenib's 3-trifluoromethyl-4-chlorophenyl part.^121^

### 4.5 Indole hybrids

Taghour *et al.* (2022) linked the thiazolidine-2,4-dione nucleus with 2-oxo-1,2-dihydroquinoline and 2-oxoindoline to generate new hybrid compounds. Out of the synthesised derivatives, compound 26 exhibited the highest potency with IC_50_ values of 116.3 nm against the VEGFR-2 enzyme. Replacing 2-oxo-1,2-dihydroquinolin with 2-oxoindolin linked to the thiazolidine-2,4-dione nucleus enhances the activity. The *in silico* docking binding free energy (Δ*G*) of compound 26 at the active site of the VEGFR-2 enzyme was −27.44 kcal mol^−1^, closer to the reference standard sorafenib's free energy of −26.30 kcal mol^−1^. Experiments utilising molecular dynamics (MD) simulations have shown that 26 exhibits a significant potential and optimal dynamics to fit tightly within the active region of VEGFR-2. The MM-PBSA analysis accurately determined the binding affinity to VEGFR-2 to be −92 kJ mol^−1^.^122^

The series of indoline-2-ones was designed by Mohamed A. Abdelgawad *et al.* (2022). The compound 27 had a VEGFR-2 kinase inhibition of 0.078 ± 0.003 μM, which is 1.78 times more effective than the reference standard sunitinib, which had an inhibitory activity of 0.139 ± 0.007 μM. The presence of the 1-phenylthiourea moiety as a hydrophobic tail in compound 27 increases the activity of VEGFR-2 compared to the 1,3,4-oxadiazole-2(3*H*)-thione moiety found in other derivatives. Compound 27 was accurately positioned at the VEGFR-2 active site, achieving a docking score of −20.1061 kcal mol^−1^.^123^

**Fig. 14:**
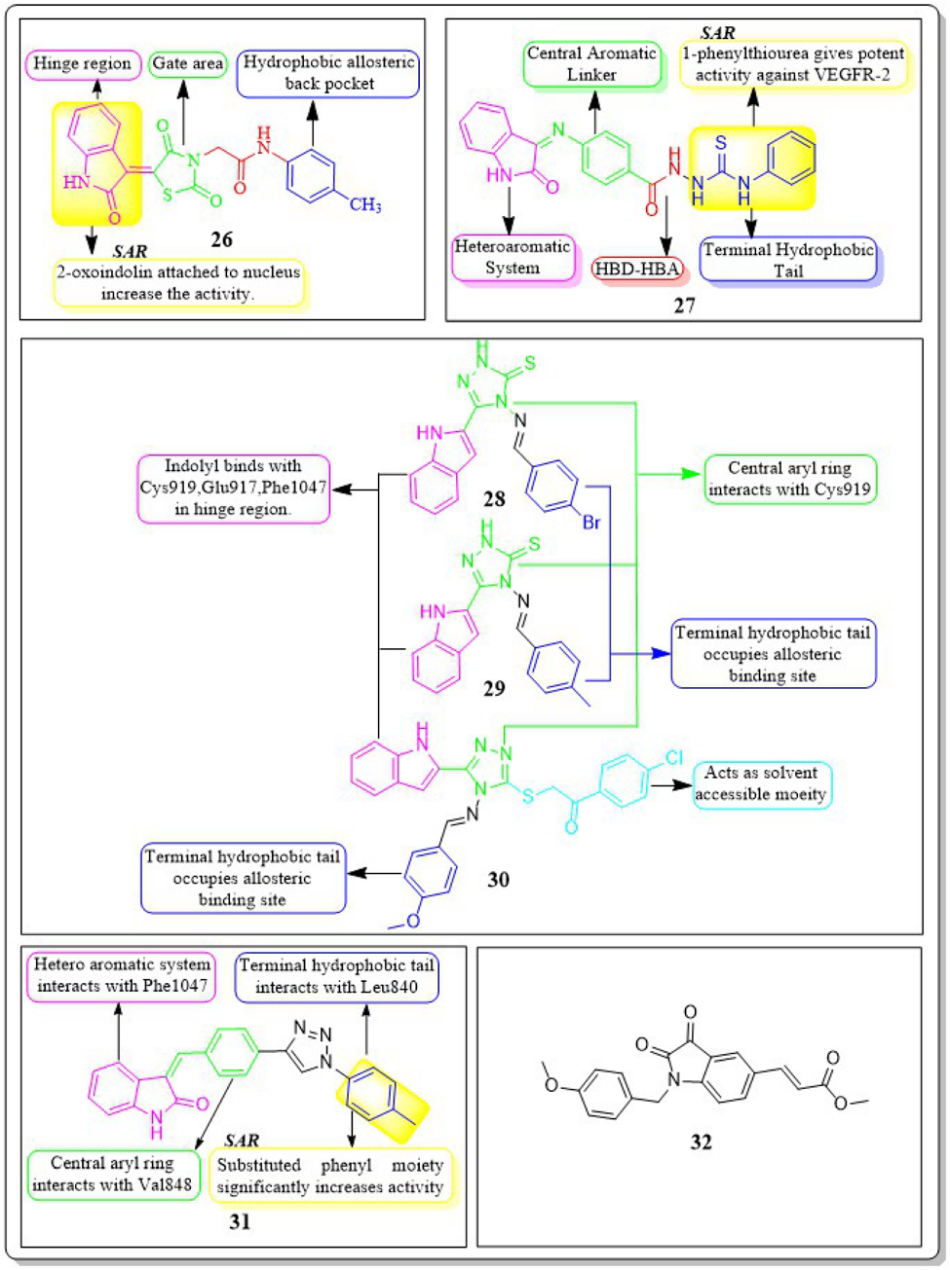
Pharmacophoric features and SAR of compounds 26 to 31.

Sami A. Al-Hussain and co-workers (2020) investigated a variety of indolyl-1,2,4-triazole hybrids for their VEGFR-2 kinase profiles. It was noteworthy that three compounds; 28, 29, and 30 had remarkable action on the VEGFR-2 enzyme (IC_50_ = 0.085 ± 0.002 μM, 0.034 ± 0.001 μM, and 0.071 ± 0.002 μM, respectively), in contrast to sunitinib, which has an IC_50_ of 0.075 ± 0.002 μM. Compound 29 performed better in an *in vitro* study against the CAKI-1 and A498 cell lines than the reference standard sunitinib (IC_50_ = 4.93 ± 0.16 μM, 1.25 ± 0.04 μM, and IC_50_ = 0.89 ± 0.04 μM, 2.2 ± 0.1 μM, respectively). Compounds 29 and 30 had docking energy scores of −7.00 kcal mol^−1^ and −8.39 kcal mol^−1^, respectively, when inserted into the active site of the VEGFR-2 kinase enzyme, comparable to sunitinib (−7.13 kcal mol^−1^). An important hydrogen link was discovered between the carbonyl oxygen of Glu917 and the NH of the indolyl molecule. Furthermore, one H-bond was provided by the Cys919 residue to the nitrogen atom in the 1,2,4-triazole core (Fig. 14).^124^

De-pu Wang *et al.* (2021)^125^ synthesised and bioevaluated a novel class of 1,2,3-triazoles. Among all the derivatives, compound 31 exhibited a lower level of toxicity towards HUVECs and had a greater capacity to inhibit kinase activity compared to sunitinib. In addition, it exerted potent inhibition on MKN-45 and HT-29 cells, with IC_50_ values of 1.92 ± 0.37 μM and 1.61 ± 0.45 μM, respectively.

Compound 31 interferes with the phosphorylation of the VEGFR-2 protein on HUVECs, proven by tube formation assessment, transwell, and western blot tests. The *in vivo* study using the zebrafish model labelled with VEGFR-2 indicated that compound 31 exhibited more anti-angiogenic action compared to sunitinib. Compound 31 demonstrated stability in binding to the active site of VEGFR-2, as indicated by the findings of docking and MD simulations. The inhibitory effect on VEGFR-2 was more pronounced when unsubstituted phenyl groups or phenyl groups substituted with electron-donating groups were introduced, as compared to analogues replaced with electron-withdrawing groups.^125^

Yunsong Chang and co-workers (2020) developed and synthesized a series of 5-(2-carboxyethenyl)isatin derivatives. These compounds were then tested to determine their impact on cell viability. Remarkably, 32 exhibited an outstanding capacity to specifically target and destroy liver hepatocellular carcinoma HepG-2 cells, with an impressive IC_50_ value of 7.13 nM. Compound 32 demonstrated significant efficacy in inhibiting HepG2 cell migration, inducing apoptosis, and arresting the cell cycle at the G2/M phase. In addition, 32 greatly decreased actin organisation and tube formation in HUVECs. Chick chorioallantoic membrane assays were utilized to assess the *in vivo* antiangiogenic effects of compound 32. It was found that VEGF and its downstream signalling pathways, including the PI3K/Akt/mTOR pathway and the mitogen-activated protein kinase pathway (ERK), were accountable for the effects caused by compound 32.^126^

New rigid sorafenib analogues based on the indole ring were developed by Rawan M. Sbenati *et al.* (2020). The most potent compound, 33, successfully inhibited VEGFR-2 (IC_50_ = 95.7 ± 3.2 nM). Surprisingly, it was noteworthy to mention that derivative 33 showed better results (IC_50_ = 8.01 μM, 4.31 μM, and 1.95 μM) than sorafenib (IC_50_ = 8.62 μM, 7.55 μM, and 7.22 μM) against Hep3B, Huh7, and Hep-G2 cell lines, respectively. The *N*-methylpiperazinyl moiety enhances the activity of VEGFR-2 inhibition. Urea moiety binds with essential amino acids (Glu885 and Asp1046) by forming two hydrogen bonds.^127^

In a recent study by Sravani Sana *et al.* (2020), a new scaffold was developed through the integration of pyrimidine and thioindole. The inhibitory potency of the indole–pyrimidine conjugate was tested, showing an IC_50_ value of 330 nM. Compound 34 demonstrated significant inhibition of the MDA-MB-231, HepG, A549, and PC-3 cell lines, with IC_50_ values of 5.85, 7.87, 6.41, and 10.43 μM, respectively. In molecular docking experiments, it was observed that compound 34 effectively formed hydrogen-bonding with the catalytically active residues Asp-1046 and Glu-885 of VEGFR-2. It was discovered that improving the benefits of VEGFR-2 inhibition requires modifications to the benzene ring. The order of reactivity is 4-Cl > 2-CH_3_-4-Cl > H > 4-OMe > 3,5-diOMe. Adding the -chloro group to the *para* position of phenyl urea 34 significantly increased the enzyme inhibition, surpassing the electron-donating and unsubstituted analogue by approximately 8 and 10 times, respectively.^128^

**Fig. 15:**
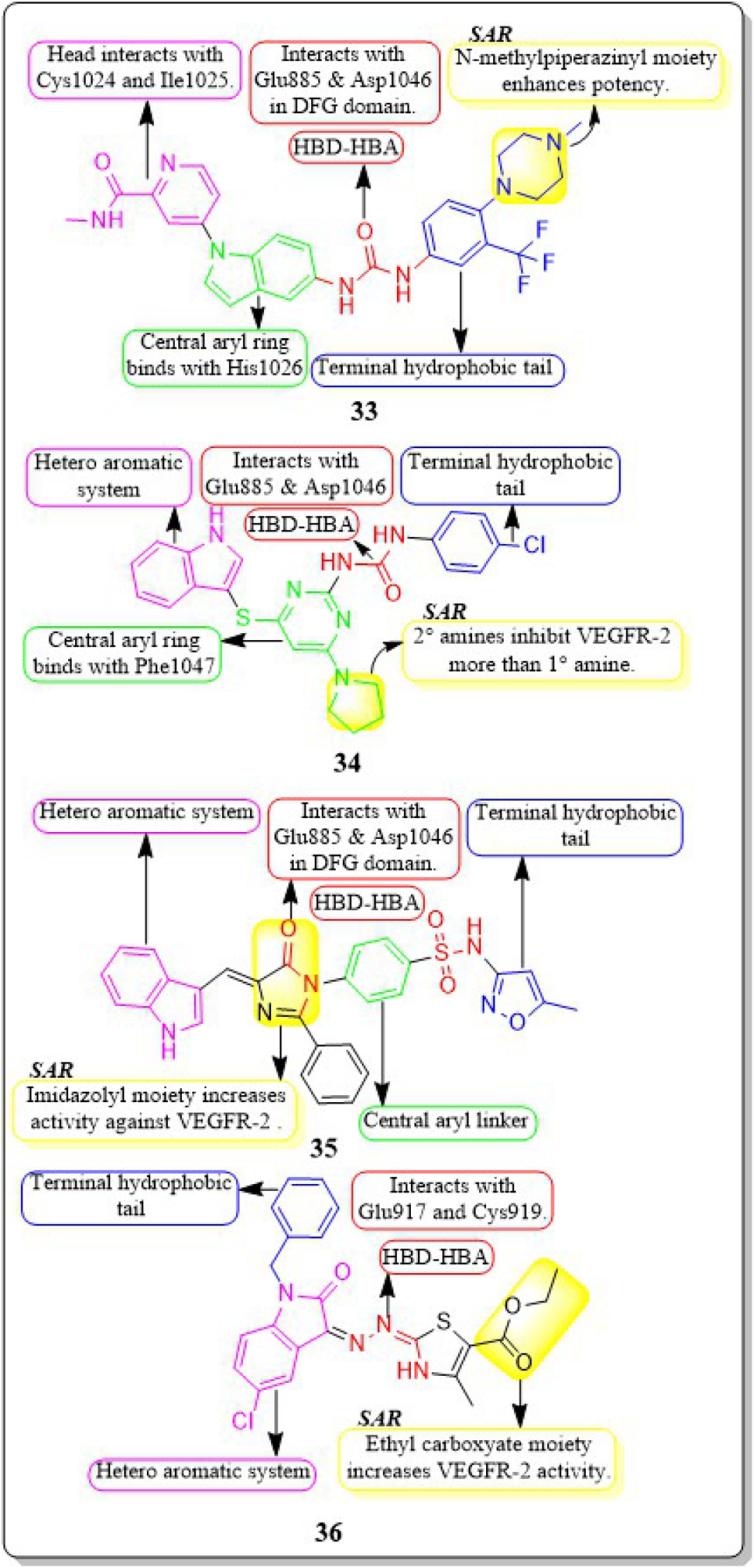
Pharmacophoric features and SAR of compounds 33 to 36.

Hanaa M. Roaiah *et al.* (2018) synthesised and analyzed several new indole derivatives. Compound 35 had superior inhibitory activity against VEGFR-2 (IC_50_ = 0.07 μM) compared to that of sorafenib's IC_50_ of 0.09 μM. Compound 35 exhibited a wide range of anticancer activity on forty-seven cell lines, with GI% values ranging from 31% to 82.5%. The docking score of 35 was found to be −15.08 kcal mol^−1^, which is similar to that of sorafenib (−15.19 kcal mol^−1^) when targeting the active site of VEGFR-2 (Fig. 15).^129^

Huda K. Mahmoud *et al.* (2020) modified and examined sunitinib analogues to assess their ability to inhibit VEGFR-2. The potency of compound 36 in inhibiting the growth of CAKI-1 and A498 cell lines was discovered to be the highest. Compound 36 exhibited substantial inhibition (IC_50_ = 0.092 ± 0.003 μM) against VEGFR-2. Compound 36, with a binding energy score of −6.70 kcal mol^−1^, tightly fits into the ATP-binding site of VEGFR-2. Phenyl substitution significantly decreases activity, while the presence of a terminal ethyl carboxylate moiety enhances it. Replacing the dihydrothiazole ring with diazane and toluyl groups enhances the inhibitory effect on VEGFR-2, but it also leads to an increase in cytotoxicity.^130,131^

A collection of new derivatives of 2-(4-(1*H*-indazol-6-yl)-1*H*-pyrazol-1-yl)acetamide has been developed, synthesised, and tested for their biological properties by Xing-Rong Wang *et al.* (2021).^131^ These compounds, 37, 38, and 39, demonstrated remarkable inhibitory effects on both VEGFR-2, with IC_50_ values of 0.73 nM, 1.4 nM, and 1.6 nM, respectively. An amide moiety on the 3rd position of indazole enhances activity compared to those with imine or amino coupling.

**Fig. 16:**
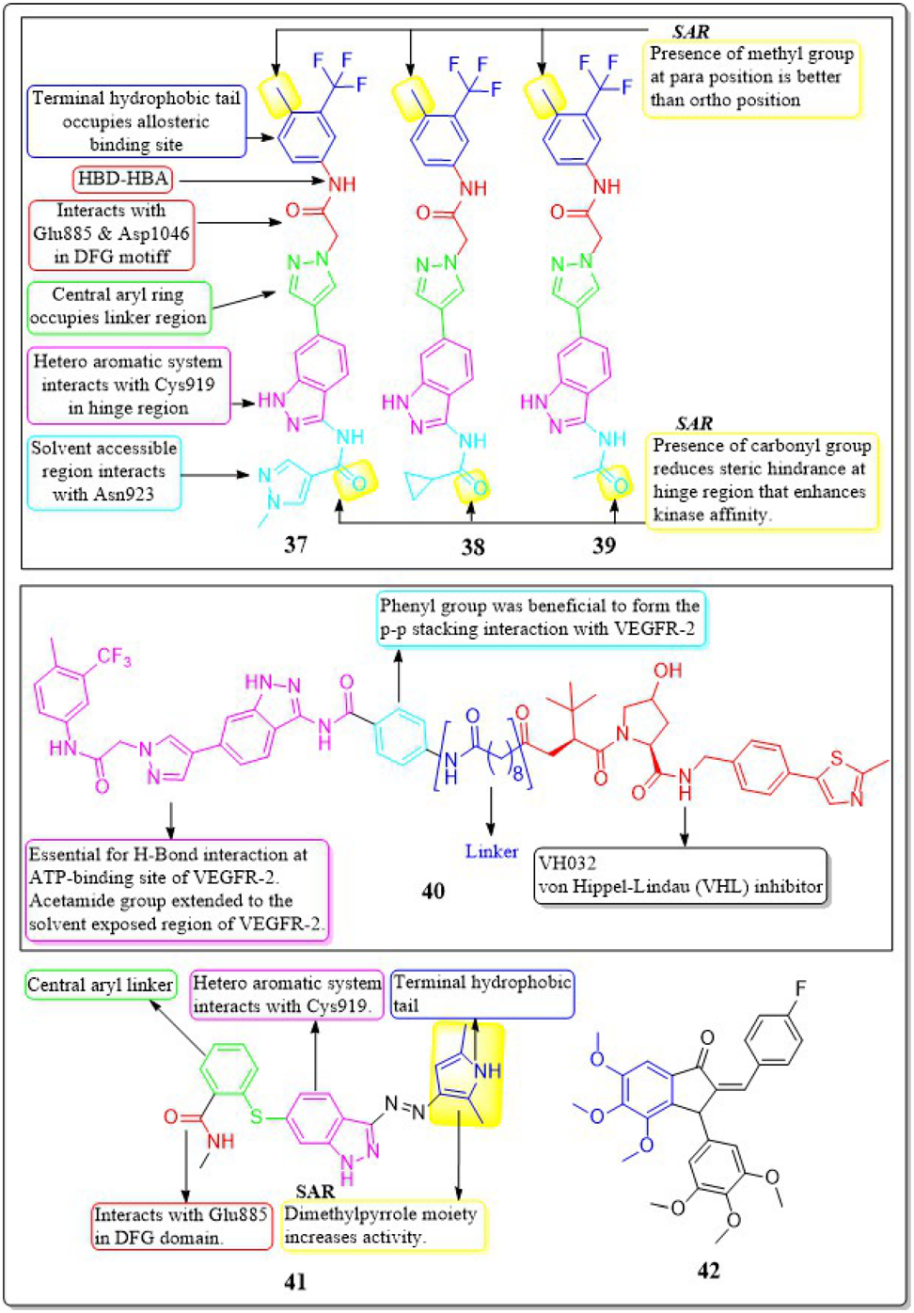
Pharmacophoric features and SAR of compounds 37 to 42.

Having a –Me group at the *para* position is more favourable than at the *ortho* position. The presence of –C=O decreases the steric hindrance in the binding region and increases the affinity of the kinase. Compounds containing an amide moiety at the 3rd position of indazole exhibit higher activity compared to those with imine or amino coupling. The compounds 37, 38, and 39 showed satisfactory anti-proliferation activity against HGC-27 tumour cells, with IC_50_ values of 0.021 ± 0.01, 0.63 ± 0.24 μM, and 0.36 ± 0.11 μM, respectively (Fig. 16).^131^

In their study, Xing-Rong Wang *et al.* (2022) investigated a group of new VEGFR-2-PROTAC degraders to improve the effectiveness of protein degradation and its anti-tumour properties. The development and production of VEGFR-2-PROTAC degraders were guided by the Lys residue zone located on the surface of the VEGFR-2 receptor. Out of all the degraders that were developed, compound 40 showed the highest level of degradation activity against the VEGFR-2 protein in HCG-27 cells when tested in a controlled environment (DC_50_: 0.084 ± 0.04 μM, *D*%: 73.7%). This led to a decrease in the amount of time it takes for the VEGFR-2 protein to be produced without affecting the expression of the mRNA of VEGFR-2 in HGC-27 cells. The 8-carbon alkanedioic acid side chain serves as a vital connector, offering sufficient flexibility to incorporate the terminal ester bond into the confined groove on the exposed part of the solvent. The substitution of the ester bond with VHL-L likely induced the ubiquitination of Lys residues, resulting in the destruction of VEGFR-2.^132^

Na Wei *et al.* (2018) synthesized axitinib analogues. With an activity of 88 nM, compound 41 outperformed the other variants. Compound 41 exhibited notable anti-proliferative effects against HUVEC cells *in vitro*, with an IC_50_ value of 99.29 ± 0.78 μM. Molecular modelling revealed that compound 41 maintained the activity *via* forming a hydrogen bonding between the amino of the pyrrole moiety and the carbonyl oxygen of the Lys920 backbone. Substituting a 4-methoxy-2,6-diaminophenyl group for the terminal dimethylpyrrole group results in a dramatic decrease in activity.^133^ The angiogenic activity of a series of fluorinated benzylidene indanones was designed, synthesised, and evaluated by Ankita Srivastava *et al.* (2020). VEGF was inhibited by 15–22% in a concentration-dependent pattern by compound 42. 17.2% VEGF inhibition was observed in MCF-7 cells, whereas 19–33% inhibition was induced by doxorubicin at different concentrations. Doxorubicin inhibited 33% of VEGF at its IC_50_.^134^

### 4.6 Thienopyrimidine derivatives

Yara El-Dash *et al.* (2021) introduced a set of fresh hybrid compounds that combine hexahydrobenzo [4,5]thieno[2,3-*d*]pyrimidine with aminothiazole scaffolds. Compound 43 exhibited remarkable potency against VEGFR-2 with IC_50_ value of 62.48 ± 3.7 nM. Based on the *in vitro* antiproliferative assay, it was found that compound 43 showed the highest potency against SNB-75, SF-295, and CAKI-1 cell lines when compared to sorafenib. When the chlorine atom in compound 43 is replaced with a bromine atom at the *para* position, there is a decrease in activity. Based on the molecular docking study, it was found that compound 43 exhibited a binding pattern that aligned with its VEGFR-2 inhibitory activity. The binding free energy of compound 43 was determined to be −9.0323 kcal mol^−1^, while the reference standard drug sorafenib had a binding free energy of −10.2499 kcal mol^−1^.^135^

In a recent study conducted by Souad A. El-Metwally *et al.* (2021), researchers explored derivatives of thieno[2,3-*d*]pyrimidines that share structural similarities with VEGFR-2 inhibitors. Compound 44 demonstrated exceptional potency, with an IC_50_ value of 0.23 ± 0.03 μM, which is comparable to the reference compound, sorafenib (IC_50_ = 0.23 ± 0.04 μM). This compound exhibits remarkable activity against HepG2, HCT-116, and the VEGFR-2 kinase enzyme, making it highly potent. On the other hand, when the electron-withdrawing group (*p*-Cl) in 44 is replaced with an electron-donating one (*p*-OCH3), it unfortunately results in a loss of potency. The docking binding free energies of synthesised compound 44 have been found to be −26.27 kcal mol^−1^ against the active site of VEGFR-2.^136^

**Fig. 17:**
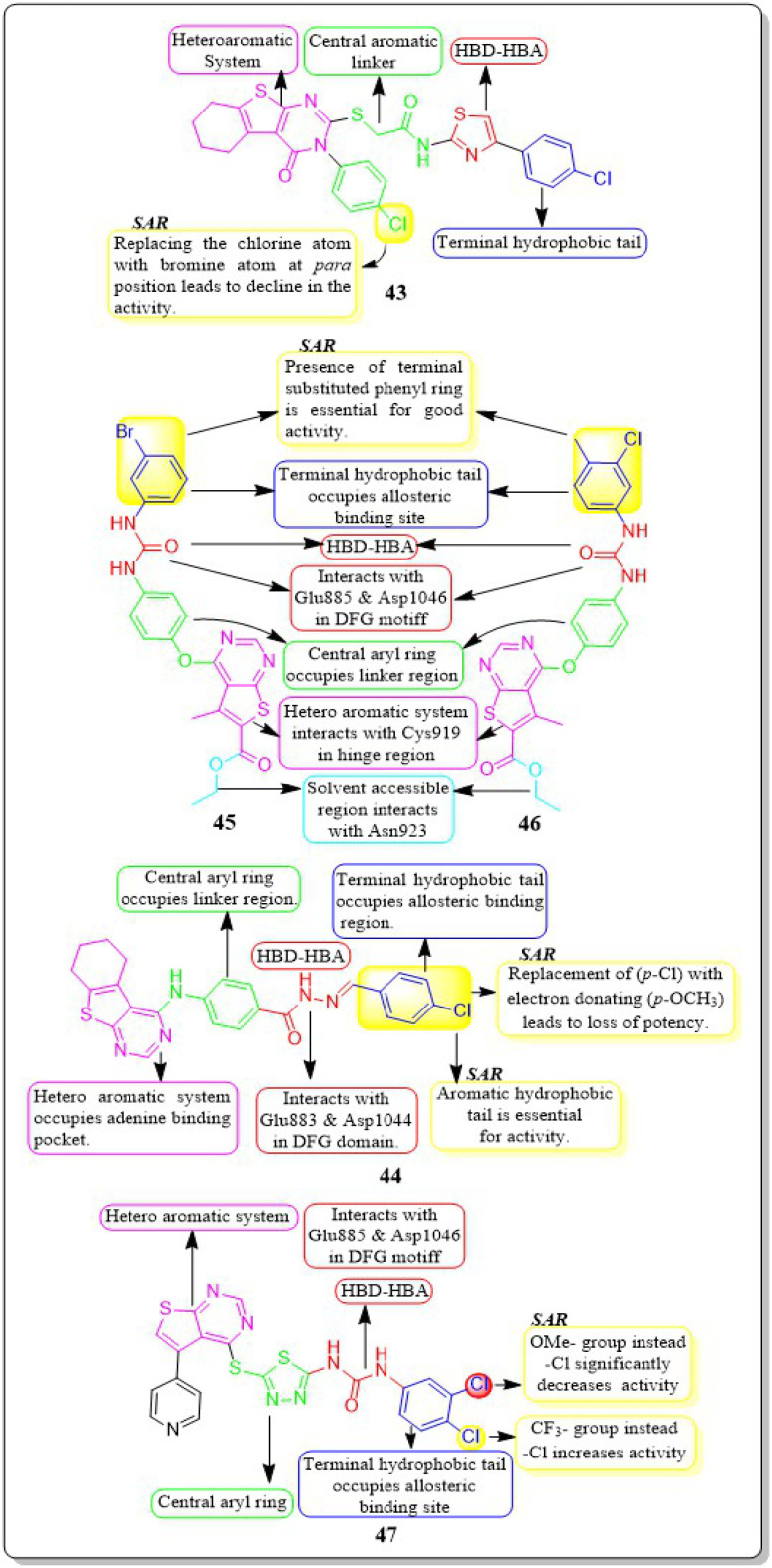
Pharmacophoric features and SAR of compounds 43 to 47.

Eman Z. Elrazaz and co-workers (2021) conducted a study where they synthesised and tested a range of 4-substituted thieno[2,3-*d*]pyrimidine derivatives. Compounds 45 and 46 displayed remarkable inhibitory activity, with IC_50_ values of 5 and 3.9 nM, respectively. A terminally substituted phenyl ring is crucial for achieving optimal activity. The ether linkage demonstrated a more pronounced inhibitory effect in comparison to the amine linker. During the molecular dynamic simulation, the average binding energy for compounds 45 and 46 was estimated to be −375.65 ± 2.31 kJ mol^−1^ and −381.08 ± 1.4 kJ mol^−1^, respectively (Fig. 17).^137^

Compounds based on thienopyrimidine that possess diaryl urea functionality were developed and synthesised by Aram Faraji *et al.* (2021).^138^ Compound 47 showed stronger anti-proliferative action against the PC3 cell line than sorafenib, according to the MTT assay.

Compound 47, with an IC_50_ value of 3.6 μM, triggered apoptosis in PC3 cells when compared to sorafenib as a standard drug. Results showed that 47 inhibited VEGFR-2 phosphorylation when tested by western blotting. VEGFR-2 phosphorylation was disrupted in a concentration-dependent manner during western blotting on the PC3 cell line, resulting in a noticeable decrease in band size at a dose of 5.4 μM. The proliferation of HUVEC cells is inhibited by compound 47, which has an IC_50_ value of 7.8 μM. It should be mentioned that compound 47 had a cytotoxic impact against the normal fibroblast Hu02 cell line that was similar to sorafenib's (IC_50_ = 40.0 ± 0.5 μM). The binding free energy values for 47 and sorafinib have been identified to be −8.49 and −8.57 kcal mol^−1^, respectively. Aram Faraji *et al.*^138^ also developed and synthesised diaryl-urea-functionalized thienopyrimidines. Out of all the compounds with an IC_50_ value of 3.6 μM, 48 showed significant activity against the PC3 prostate cancer cell line.

Compound 48 exhibited a roughly fourfold inhibition of PC3 cell proliferation in comparison to sorafenib. The CAM assay demonstrated a significant 48% inhibition of blood vessel development. The western blot analysis for compound 48 demonstrated the suppression of VEGFR-2 phosphorylation. In addition, compound 48 had a similar cytotoxic impact against the normal fibroblast Hu02 cell line (IC_50_ = 34.3 ± 0.3 μM) as sorafenib (IC_50_ = 40.0 ± 0.5 μM). The computed binding free energies for 48 and sorafenib were −8.49 kcal mol^−1^ and −8.57 kcal mol^−1^, respectively.^138^

Amna Ghith *et al.* (2018) synthesised and assessed a number of new thieno [2,3-*d*] pyrimidine derivatives. With an IC_50_ of 2.27 μM, the most powerful derivative, 49, showed noteworthy efficacy against VEGFR-2. These findings were also explained by molecular docking experiments, which showed that the urea-based derivatives were able to build an important network of contacts with the residues Cys919, Glu885, and Asp1046. For maximum activity, a terminal substituted phenyl ring is required.^139^

Rasoul Motahari *et al.* (2022) have successfully designed and synthesised new variations of tetrahydropyridothienopyrimidine-based compounds. Compound 50 exhibited significant action against MCF-7, PC-3, SW480, HEPG-2, HUVEC, MRC5, and MCF7 cell lines, with IC_50_ values of 2.67 ± 0.21 μM, 11.35 ± 0.09 μM, 6.84 ± 0.05 μM, 7.20 ± 0.03 μM, 2.09 ± 0.08 μM, 38.10 ± 0.81 μM, and 2.67 ± 0.21 μM, respectively. Compound 50 demonstrates a little less potent inhibitory effect on developing CAM in comparison to the positive control.^140^

### 4.7 Pyrimidine motifs

Asmaa M. Sayed *et al.* (2021) designed a series of sulfonamides equipped with hydrazone linked to dimethyl and/or diethyl malonates. Compounds 51–53 were discovered to be the most effective derivatives, with IC_50_ values of 0.14 ± 0.02 μM, 0.15 ± 0.02 μM, and 0.15 ± 0.02 μM, respectively, showing the greatest inhibition of VEGFR-2. Compounds 51–53 were discovered to have higher anticancer activity than the other compounds due to the presence of their heteroaromatic pyrimidine, isoxazole, and pyrazolidine moieties, as well as their diazene linkers. The strongest VEGFR-2 inhibitory activity was found in compound 51, which has a six-membered heteroaromatic pyrimidine ring and pyrazolidine tail, as contrasted with compounds 52 and 53, which only had one of the two aforementioned rings. Pyrimidine 51, a heteroaromatic ring, showed more VEGFR-2 inhibitory action than izoxazole 52. Compounds 51–53 have yielded binding free energy (Δ*G* in Kcal mol^−1^) of −119.58, −119.12, and −119.05, respectively. Compounds 51–53 exhibited the highest potency among all the derivatives tested against the three cancer cell lines, HepG2, HCT116, and MCF-7, with IC_50_ values of (6.43 ± 0.5, 9.66 ± 0.8, 10.57 ± 0.9 μM), (8.65 ± 0.7, 7.49 ± 0.6, 14.29 ± 1.3 μM), and (8.97 ± 0.7, 10.13 ± 0.9, 13.82 ± 1.1 μM), respectively.^141^

In their study, Ghada H. Al-Ansary *et al.* (2021) developed a novel set of biphenylurea/thiourea derivatives conjugated with heteroarylsulfonamide motifs. The researchers then examined the vitality of HUVEC, conducted a migration test, and performed western blot analysis using sorafenib as a reference standard. The synthesised compounds showed more efficacy than sorafenib in all three assays. Compound 54 exhibited superior antiproliferative activity against HUVECs, with an IC_50_ value of 10.54 μM, in comparison to sorafenib (IC_50_ = 17.74 μM). The investigated compounds exhibited a notable suppression of HUVEC cell migration mediated by VEGF, with the most significant effect reported at a dose of 10 μM. Compound 54 has shown a higher potency, inhibiting up to 86% of cell migration compared to the reference drug sorafenib, which only inhibited 75.56%. The compounds were evaluated *in vitro* for their cytotoxic effects on MCF-7, HepG2, CaCo-2, and HCT-116 cancer cell lines, as well as normal RPE1 cells. The results indicated that the compounds have dual properties, acting as both antiangiogenic and cytotoxic agents.^142^

**Fig. 18:**
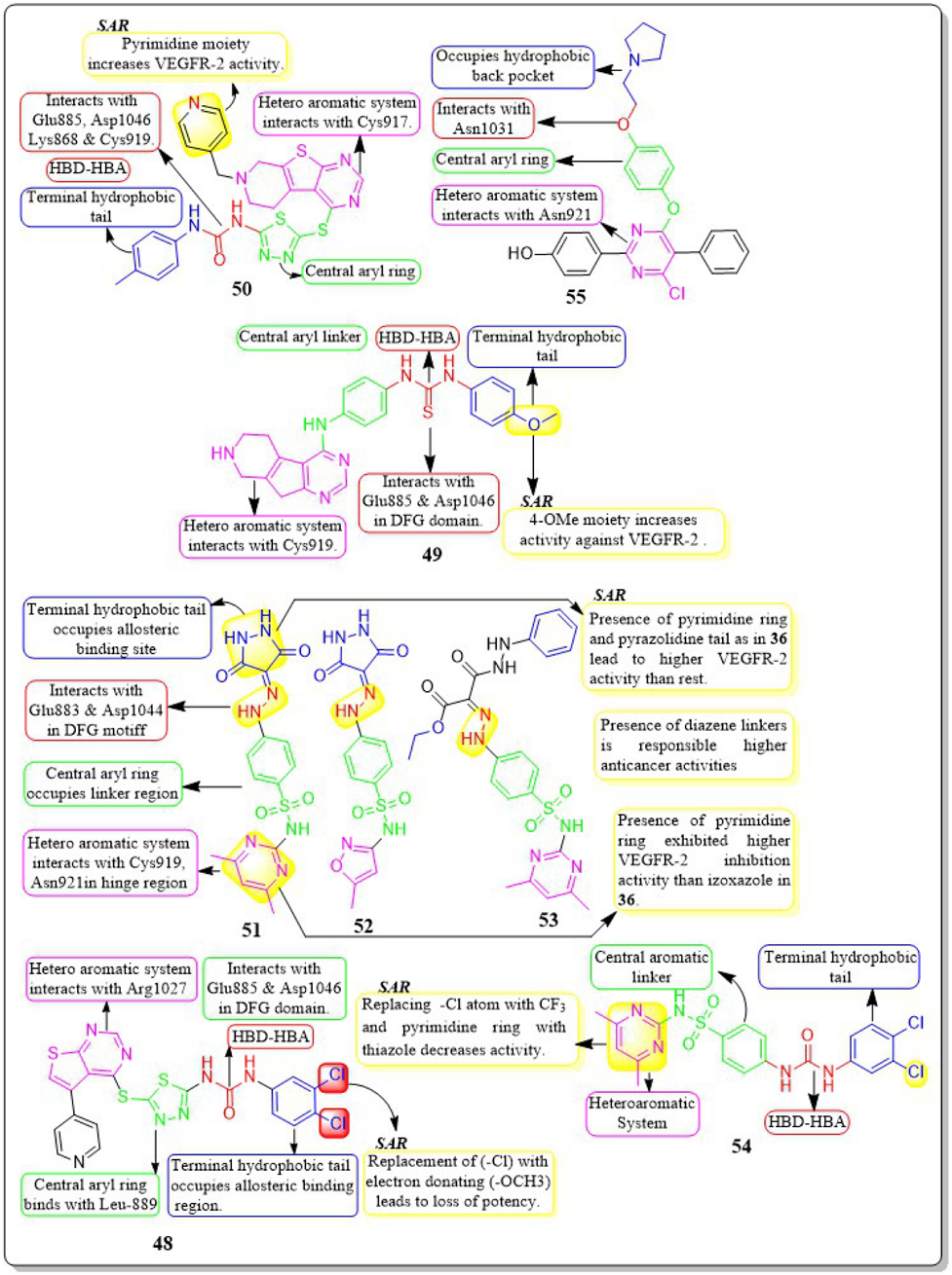
Pharmacophoric features and SAR of compounds 48 to 55.

Guoshun Luo *et al.* (2018) developed and synthesised a variety of 2,4-disubstituted pyrimidines. Compound 55 had the highest potency against the VEGFR-2 enzyme (IC_50_ = 0.085 μM). 55 effectively inhibited the Raf-1/MAPK/ERK pathway, causing apoptosis and suppressing migration in MCF-7 and Ishikawa cells (IC_50_ = 0.81 μM and 5.93 μM, respectively). Furthermore, 55 substantially decreased blood vessel formation in CAM while also inhibiting VEGFR-2 protein expression (Fig. 18).^143^

Mahitab K. Sobhy *et al.* (2019) designed and synthesised a group of 6,7-dihydro-5*H*-cyclopenta[*d*]pyrimidines, which were then evaluated for anticancer activity. Compound 56 demonstrated VEGFR-2 inhibition with an IC_50_ value of 0.85 μM. Compound 56 exhibited a significant pharmacophore mapping fit value of 9.21 and a greater docking score of −29.29 kcal mol^−1^. The effectiveness diminishes when the third position of the terminal phenyl ring contains a methyl group instead of a trifluoromethyl group.^144^

Wuji Sun *et al.* (2018) synthesized a new series of derivatives based on pyrimidine. Inhibitory activity against VEGFR-2 was observed to be greater in compound 57 (IC_50_ = 0.23 M) than in the reference standard pazopanib (IC_50_ = 1.04 M). It is worth noting that compound 57 exhibited remarkable cellular potencies against the A549 and HepG2 cell lines (IC_50_ = 13.17 μM and 11.94 μM, respectively), in contrast to Pazopanib (IC_50_ = 21.18 μM and 36.66 μM). In contrast to Pazopanib (Δ*G* = −10.28 kcal mol^−1^), Compound 57 (Δ*G* = −10.37 kcal mol^−1^) demonstrated notably superior binding capacities. These results corroborated the compound's exceptional inhibitory potency against the A549 and HepG2 cell lines.^145^

A new group of substituted 4-amino-2-thiopyrimidines was developed, synthesised, and assessed by Heba T. Abdel-Mohsen *et al.* (2019). Compound 58 demonstrated impressive inhibitory activity against VEGFR-2 (IC_50_ = 0.17 μM) and BRAF (IC_50_ = 0.15 μM). Compound 58 showed significant inhibition of MCF7 and T-47D cell lines, with IC_50_ values of 13.02 μM and 2.18 μM, respectively. The compound 58 exhibited a VEGFR-2 inhibition of 46.00 4.11 ng mL^−1^ against the MCF7 cell line, resulting in an impressive 84 percent inhibition. Through molecular docking, the interaction pattern of the co-crystalized ligand in VEGFR-2 binding sites was accurately replicated, yielding energy scores of −15.19 kcal mol^−1^. Furthermore, the docking poses accurately replicated the crucial interactions within the binding site regions of VEGFR-2 (Glu885, Cys919, and Asp1046).^146^

**Fig. 19:**
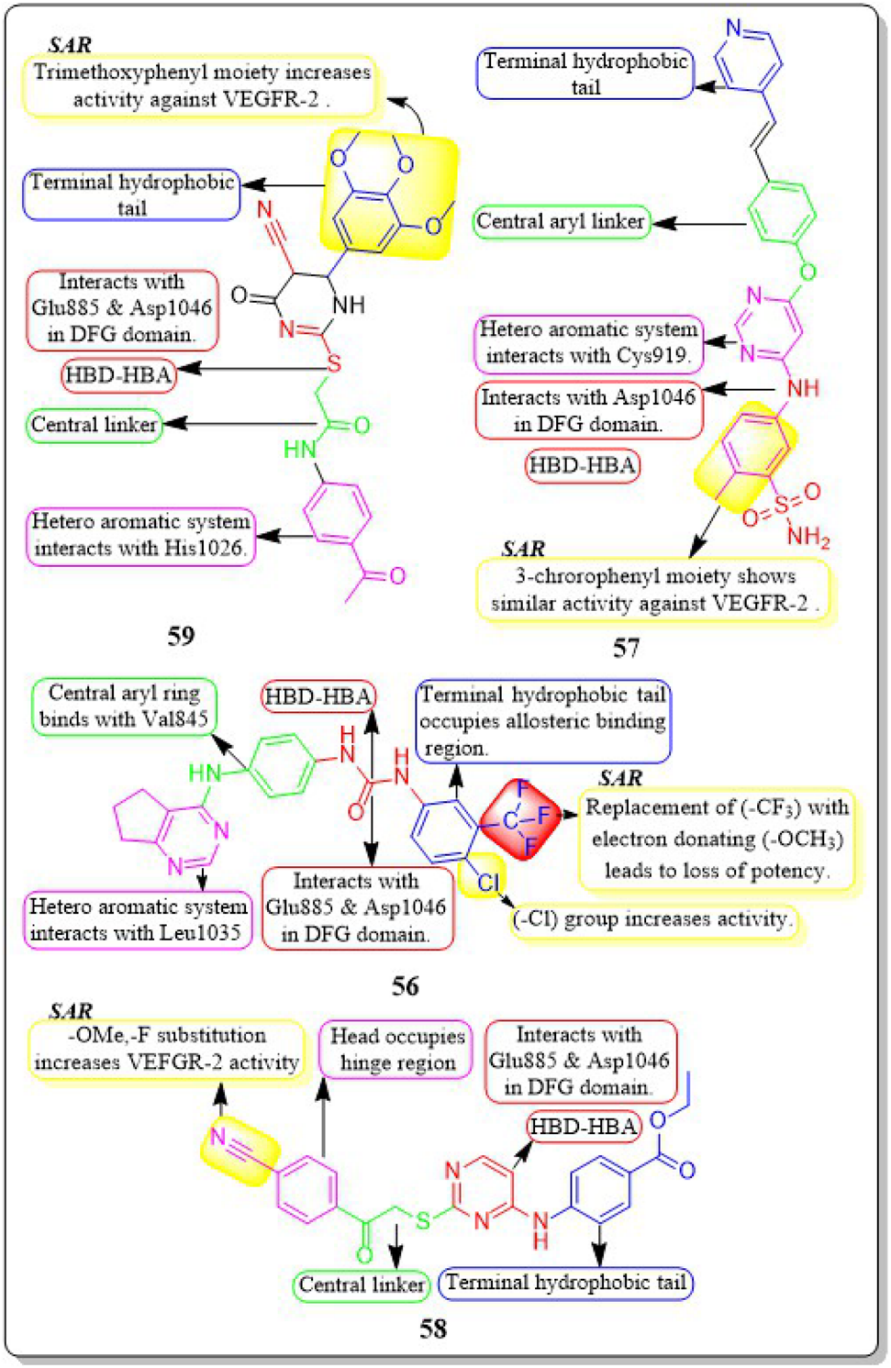
Pharmacophoric features and SAR of compounds 56 to 59.

A group of researchers led by Adel A. Marzouk *et al.* (2020) synthesized and bio-evaluated novel 1,6-dihydropyrimidin-2-thiol derivatives. Compound 59 exhibited remarkable potency, displaying an IC_50_ value of 198.7 nM against VEGFR-2. The docking study revealed that the new compounds fit well into the active site of VEGFR-2, with binding free energies ranging from −9.80 to −11.25 kcal mol^−1^. This is slightly lower than the binding free energy of sorafenib, which was −12.12 kcal mol^−1^. Through *in vitro* five-dose tests, it was observed that the GI_50_ values ranged from 19 to 100 μM, indicating its potency. Additionally, the selectivity ratios at the GI_50_ level ranged between 0.75 and 1.71, further highlighting its effectiveness (Fig. 19).^147^

### 4.8 Pyrrolo[2,3-*d*] pyrimidine analogues

Mai Adel *et al.* (2022)^148^ afforded a new set of compounds by connecting pyrrolo[2,3-*d*]pyrimidine to flurorinated diarylureas, which were then tested for their VEGFR-2 activity. The compound 60 exhibited the highest level of action, with a potency of 52.4 nM, compared to the IC_50_ of 78.9 nM for the standard drug sorafenib. The substitution of a trifluoromethyl group on the *meta* position of the benzene ring enhances the activity, whereas the presence of a halogen and/or methoxy moiety on the benzene ring decreases the activity against VEGFR-2.

The docking investigation demonstrated that the synthesised compounds effectively occupied the binding site of VEGFR2, with docking scores ranging from −8.76 to −10.28 kcal mol^−1^. This is comparable to the binding free energy of sorafenib, which is −10.12 kcal mol^−1^. In addition, Mai Adel *et al.* (2022) synthesized compounds based on pyrrolo[2,3-*d*]pyrimidine as inhibitors of VEGFR-2. The pyrrolo[2,3-*d*]pyrimidine derivatives (61 and 62), which have an m-toluyl urea tail connected through a NH or ether linker, exhibited the most potent nanomolar inhibition against VEGFR-2. Specifically, compounds 61 and 62 displayed an IC_50_ value of 11.9 nM and 13.7 nM, respectively, superior to that of sorafenib (IC_50_ = 90 nM). Compounds 61 and 62 exhibited antiproliferative action against HUVEC cells, with IC_50_ values of 0.31 ± 0.01 μM and 3.74 ± 0.18 μM, respectively.^148^

A series of pyrazolo[3,4-*d*] pyrimidines was discovered and evaluated by Dan-Xia Ying *et al.* (2022). It is interesting to note that compound 63 showed higher activity against the HepG2 and T47D cell lines (IC_50_ = 5.90 ± 0.06 μM and 5.57 ± 1.55 μM, respectively) compared to sorafenib (IC_50_ = 9.05 ± 0.54 μM and 7.41 ± 3.08 μM). Docking studies showed that 63 exhibited the ability to form two hydrogen bonds with the Cys919 and Asp1046 residues at the active sites of VEGFR-2, mirroring the behaviour of sorafenib. Replacing the terminal chlorophenyl moiety with a toluyl moiety results in a notable decrease in VEGFR-2 activity, whereas having the chlorophenyl moiety enhances the activity.^149^

**Fig. 20:**
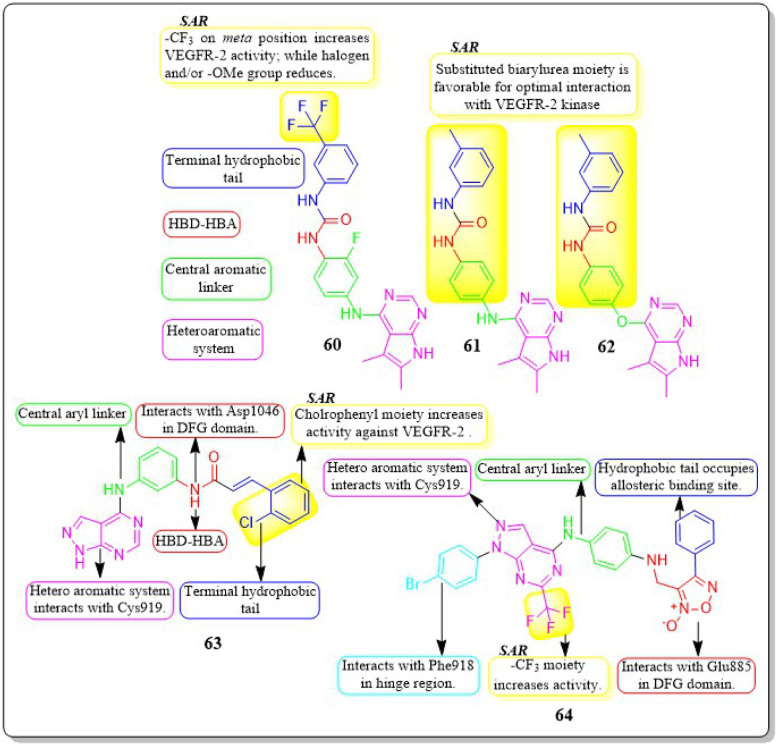
Pharmacophoric features and SAR of compounds 60 to 64.

In their recent study, Mater H. Mahnashi *et al.* (2022) introduced a set of 1,2,5-oxadiazole-2-oxides. Compound 64 displayed remarkable activity, with an IC_50_ value of 0.092 μM, demonstrating VEGFR-2-inhibitory potential similar to that of sorafenib (IC_50_ = 0.049 μM). In addition, compound 64 exhibited superior anticancer activity when compared to the reference drug, sorafenib. It displayed IC_50_ values of 13 μM, 11.5 μM, and 11.6 μM against the MDA-MB-231, HepG-2, and A2780CP cell lines, respectively. The free energy of binding for 64 was found to be −11.3747 kcal mol^−1^ (Fig. 20).^150^

### 4.9 pyrazolo[3,4-*d*] pyrimidine hybrids

Zukela Ruzi and co-workers (2022) discovered a group of new pyrazolo[3,4-*d*] pyrimidine analogues. It is worth noting that compound 65 exhibited stronger inhibition of VEGFR-2 (IC_50_ = 13.18 ± 0.17 nM) compared to the reference standard sunitinib (IC_50_ = 14.2 ± 0.02 nM). Compound 65 showed IC_50_ values of 0.03 ± 0.01 μM, 0.04 ± 0.03 μM, 0.19 ± 0.01 μM, 0.09 ± 0.01 μM, and 1.61 ± 0.02 μM against HT-29, HCT-116, HGC-27, HeLa, and MDA-MB-231 cell lines, respectively. Additionally, it effectively inhibited the migration, adhesion, and tube formation activities of HUVEC cells. Compound 65 exhibits affinity for the VEGFR-2 protein, specifically targeting Val-848, Phe-1047, Leu-1035, Cys-919, and Phe-918 amino acid residues as its primary binding sites. The binding energy for 65 was lower than −8.30 kcal mol^−1^.^151^

The researchers Yuanyuan Wang *et al.* (2018) developed, synthesised, and conducted biological evaluations on a set of 1*H*-pyrazolo[3,4-*d*]pyrimidine derivatives. Compound 66 exhibited significant inhibitory effects on the growth of BRAFV600E-expressing A375 (IC_50_ = 1.74 μM) and H-29 (IC_50_ = 6.92 μM) cells, as well as VEGFR-2-expressing HUVEC (IC_50_ = 5.89 μM). Compound 66 exhibited potent inhibition against BRAFV600E (IC_50_ = 0.171 μM) and VEGFR-2 (IC_50_ = 0.779 μM) and showed notable anti-proliferative effects on three cell lines (HUVEC, A375, and HT-29). Replacing a –CH_3_ group on the first position of the pyrazolopyrimidine ring is found to be more efficacious than using ethyl and isopropyl. Replacing the benzene ring at the *para* position is significantly more efficient. The binding-free energies were determined through calculations utilising the MM-PBSA and MMGBSA programmes. It was discovered that 66 demonstrated reduced effectiveness as a ligand in comparison to sorafenib.^152^

Qiumeng Zhang *et al.* (2018) developed and synthesised pyrazolo[4,3-*b*]pyrimido[4,5-*e*]^1,4^ diazepines. Out of these, compound 67 had the highest level of effectiveness as a VEGFR-2 inhibitor, with a potency of 8.3 ± 4.7 nM. The morpholine derivative of compound 67 exhibited notable inhibitory effects on VEGFR-2, Aurora A, and Aurora B, with IC_50_ values of 21.6 nM, 46.2 nM, and 37.6 nM, respectively. The anti-proliferative efficacy of a morpholine derivative was assessed against various human gastric cancer cell lines, including SNU-5, MKN-45, MKN-74, SGC-7901, and BGC-823.^153^

Menna M.A. *et al.* (2021) synthesised a number of fluro[3,2-*e*][1,2,4]triazolo[1,5-*c*]pyrimidines and furo[2,3-*d*]pyrimidines and tested them for their ability to inhibit VEGFR-2 in a laboratory setting. These compounds showed effective inhibition in the nanomolar range, with some demonstrating enhanced ligand efficiencies. Compound 68 exhibited significant activity with an IC_50_ value of 38.72 ± 1.7 nM, while compound 69 showed substantial activity with an IC_50_ value of 41.40 ± 1.8 nM.

**Fig. 21:**
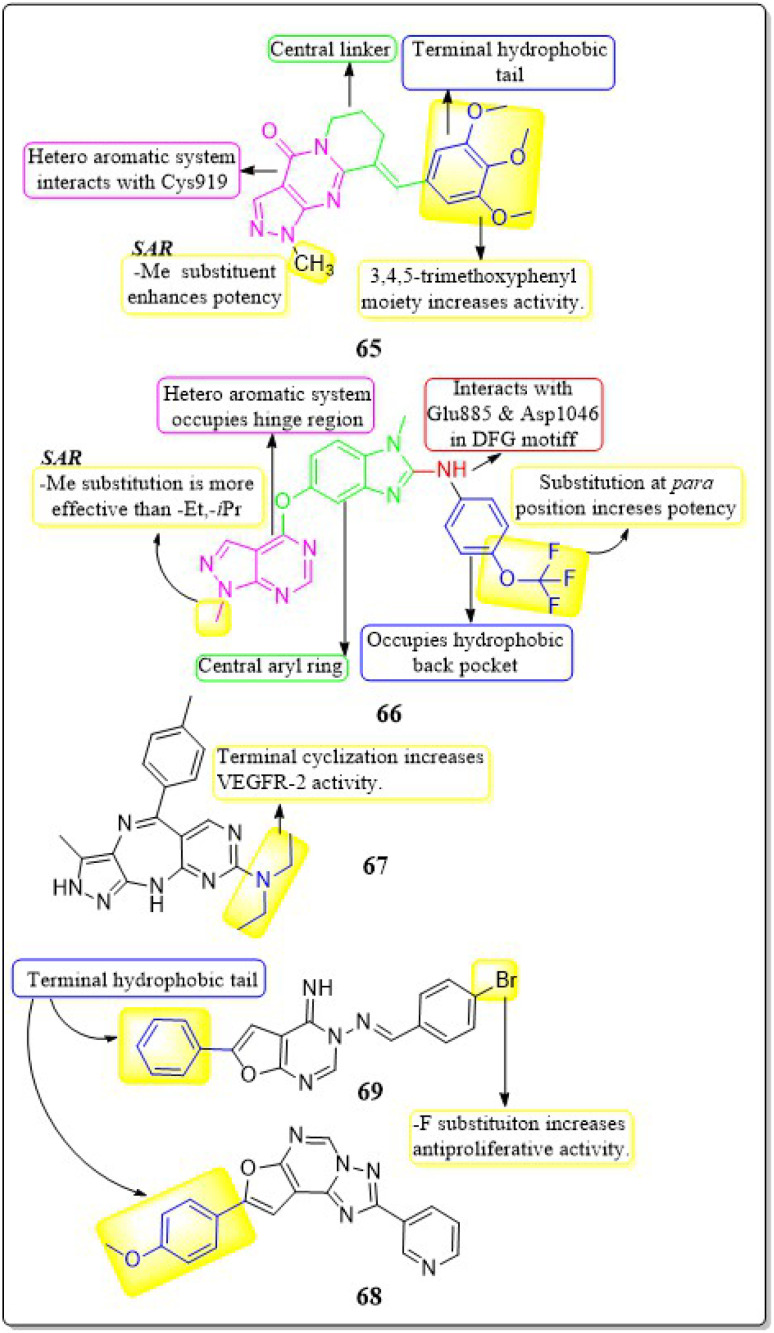
Pharmacophoric features and SAR of compounds 65 to 69.

These values were compared to sorafenib, which had an IC_50_ value of 41.24 ± 1.9 nM. Compound 68 exhibited superior antiproliferative activity in the HUVEC cell assay, with an IC_50_ value of 17.37 ± 1.03 μM, compared to sorafenib, that had an IC_50_ value of 20.64 ± 1.22 μM. The docking investigation demonstrated that the compounds 68 and 69 exhibited a binding free energy of −8.00 kcal mol^−1^ and −7.58 kcal mol^−1^, respectively, at the active site of VEGFR-2 (Fig. 21).^154^

### 4.10 Pyridine derivatives

Ahmed *et al.* (2021) synthesised a new anticancer pyridine-sulfonamide scaffold. Compound 70 was found to be an effective VEGFR-2 inhibitor (IC_50_ = 3.6 μM) compared to sorafenib (IC_50_ = 4.8 μM). Annexin V-FITC/PI experiments and DNA flow cytometry showed that hybrid 70 disrupted the renal UO-31 cell cycle and induced apoptosis. Compound 70, with a docking score of −27.09 kcal mol^−1^, strongly binds to the VEGFR-2 active site. The pyridine moiety interacted hydrophobically with Leu1033, Ala864, and Cys917. The terminal phenyl group made two hydrophobic connections with Cys1022 and Ile886. With Glu883 and Asp1044, the sulfonamide group created two hydrogen bonds. The phenyl (spacer) group interacted hydrophobically with Cys1043, Val914, Val89, Lys886, and Phe1045. The *meta*-fluro is active against all cell lines.^155^

A unique series of thiourea-azetidine hybrids was developed by Deepa R. Parmar *et al.* (2021) and evaluated against a range of human cancer cell lines. With EC_50_ values of 0.03, 0.25, 0.6, and 0.03 μM, respectively, compound 71 was shown to be the most effective member against 786-O, PC3, U251, and A431 cancer cell lines. It also demonstrated greater potency than doxorubicin in PC3, A431, and 786-O cell lines. The inhibitory action significantly increased upon the insertion of a methoxy group. 71's binding free energy (Δ*G* in kcal mol^−1^) has been estimated to be −21.12 kcal mol^−1^.^156^

In a recent study, Eslam B. Elkaeed *et al.* (2022) successfully synthesised and evaluated a series of nicotinamide-based derivatives. This compound, 72, showed impressive inhibitory potential against VEGFR-2 in laboratory tests, with a potency of 51 nM. It also displayed promising cytotoxicity against MCF-7 and HCT-116 cancer cell lines, with IC_50_ values of 8.25 μM and 6.48 μM, respectively. These results indicate a high level of selectivity, with selectivity indexes of 12.89 and 16.41 for the two cell lines. Through DFT studies, the binding mode of compound 72 with VEGFR-2 was confirmed. The MM-GBSA analysis further supported the proper binding, revealing a total binding energy of −38.36 kcal mol^−1^.^157^

Amal Abdel Haleem *et al.* (2020)^158^ performed the synthesis of molecules based on the 3-cyano-6-naphthylpyridine scaffold. These derivatives were designed to specifically block VEGFR-2. Compound 73 exhibited the highest potency compared to all other synthesised derivatives.

**Fig. 22:**
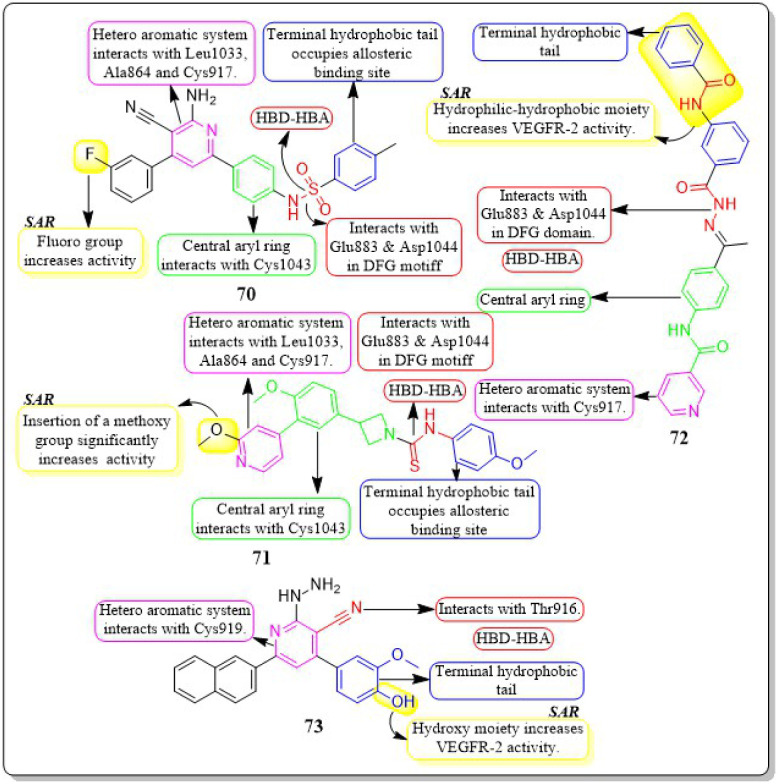
Pharmacophoric features and SAR of compounds 70 to 73.

Delphinidin, a drug with well-established inhibitory effects on VEGFR-2, served as a reference drug. Compound 73 outperformed the reference standard, which is worth mentioning. Compound 73 effectively blocked the activity of VEGFR-2 in laboratory tests and computer simulations, with an IC_50_ value of 0.19 ± 0.01 nM and a binding energy score of −9.9868 kcal mol^−1^, while delphinidin showed VEGFR-2 inhibition at an IC_50_ value of 5.09 ± 0.42 nM with a docking score of −8.2655 kcal mol^−1^. Compound 73 exhibited superior inhibition of PC3, DU145, MCF-7, and MDA-MB435 cell lines compared to the reference standard Doxorubicin. The IC_50_ values for compound 73 were 55 ± 3.1 nM, 8.5 ± 0.43 nM, 0.5 ± 0.001 nM, and 96 ± 1.6 nM, respectively, while the IC_50_ values for doxorubicin were 59 ± 2.1 nM, 1.8 ± 0.01 nM, 11 ± 0.37 nM, and 69 ± 1.37 nM, respectively (Fig. 22).^158^

### 4.11 Benzimidazole analogues

Islam H. Ali *et al.* (2023) studied 2-arylbenzimidazole-thiopyrimidine and -thioquinazolin-4(3*H*)-one conjugates. The benzimidazole-quinazolinone compounds have shown significant anticancer activity (GI_50_ = 1.3−4.2 μM) against tested cancer cell lines. In a VEGFR-2 kinase test, compound 74 had the highest potency at 6.14 μM. Any substitution on the 2-arylbenzimidazole nucleus diminishes activity, but the *p*-tolyl substitution on the third position, 4-oxo-3,4-dihydroquinazolin boosts it. Compounds 74 had a docking score of −14.82 kcal mol^−1^ at the VEGFR-2 binding site, compared to the native ligand sorafenib, which had a binding value of −15.19 kcal mol^−1^, reflecting their experimental inhibitory efficacy.^159^

An investigation conducted by Ayman Abo Elmaaty *et al.* (2023)^160^ showed the repurposing of thirteen FDA-approved benzimidazole anthelmintic medicines as VEGFR-2 antagonists. Based on the investigation involving molecular docking and molecular dynamic simulations against VEGFR-2, three benzimidazoles (fenbendazole 75, mebendazole 76, and albendazole 77) were identified as possible VEGFR-2 antagonists. Furthermore, these drugs demonstrated increased efficacy in inhibiting the growth of MCF7, A549, and HUH7.

In addition, to boost the solubility of mebendazole in water, it was synthesised as mixed micelles (MMs). These MMs exhibited improved drug release and demonstrated more promising cytotoxicity results in a cell-based VEGFR-2 assay compared to the unrefined mebendazole. Compounds 75–77 exhibited favourable interactions with VEGFR-2 in both docking and MD modelling tests. An ELISA was used to quantify VEGFR-2 in treated HUH7 cells; the results showed that all tested drugs significantly decreased the concentration of VEGFR-2. The greatest inhibition of VEGFR-2 was observed in MBZ-loaded MMs, with a concentration of 860.8 ± 312 pg mL^−1^. This was even better than the reference drug, sorafenib, which had a concentration of 1073 ± 41.1 pg mL^−1^.^160^

A group of researchers, led by Amany S. Mostafa *et al.* (2018), developed and evaluated a novel set of 2-phenylbenzimidazoles. Compound 78 exhibited the highest level of VEGFR-2 inhibitory activity (IC_50_ = 6.7 ± 1.3 nM) against the MCF7 cell line, surpassing the reference standard Sorafenib (IC_50_ = 7.6 ± 2.8 nM). Compound 78 demonstrated considerable inhibition of the MCF-10F, BJ, and MRC-5 cell lines, with IC_50_ values of 33.1 ± 1.8 μM, 40.6 ± 2.5 μM, and 17.3 ± 0.4 μM, respectively. This inhibition was comparable to that of the reference standard doxorubicin, which had IC_50_ values of 22.6 ± 2.7 μM, 17.2 ± 0.7 μM, and 15.2 ± 1.1 μM for the same cell lines, respectively. The presence of a nitro group on the fourth position of the terminal aryl ring enhances the activity of VEGFR-2.^161^

**Fig. 23:**
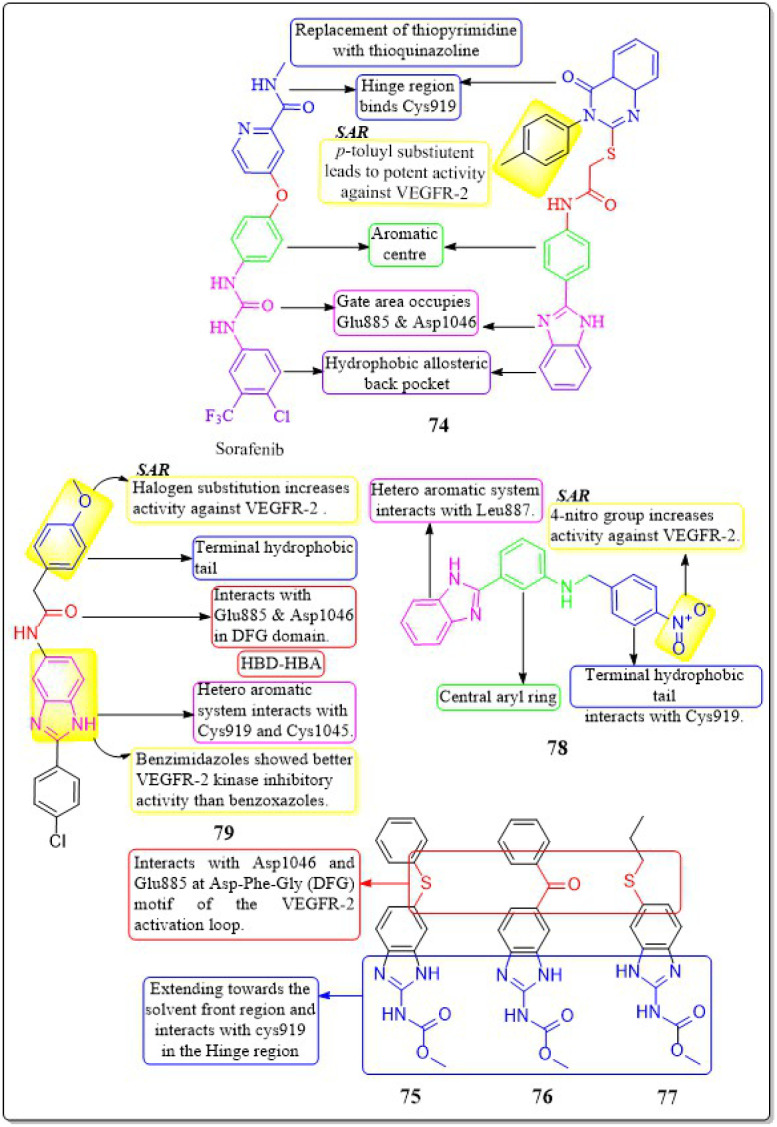
Pharmacophoric features and SAR of compounds 74 to 79.

Xu Yuan *et al.* (2019) developed, synthesised, and tested a new family of benzimidazole compounds. Compound 79 exhibited the highest levels of inhibition towards VEGFR-2 kinase, HUVEC, and HepG2 cells, with IC_50_ values of 51.4 nM, 1.47 μM, and 2.57 μM, respectively. Compound 79 has shown significantly higher anti-angiogenic effects compared to sorafenib. The SAR investigations demonstrated that the addition of halogen atoms to the end of the phenyl group enhances the activity of VEGFR-2 (Fig. 23).^162^

### 4.12 Naphthalene derivatives

Em Cahn *et al.* (2022) synthesised a range of 2-naphthamide derivatives and assessed their antibacterial, antifungal, and anticancer properties in laboratory settings. Compound 80 demonstrated high inhibitory activity for VEGFR-2 in laboratory tests, with an IC_50_ value of 0.384 μM, while sorafenib had an IC_50_ value of 0.069 μM. The inclusion of *N*-(4-chlorobenzyl), 4-hydroxy, and 5,7-dimethoxy groups in the 2-naphthamide structure is preferable for increasing the effectiveness of the compound in fighting bacterial infections and tumours. In the docking simulation, compound 80 exhibited a binding affinity of −9.8 kcal mol^−1^ with VEGFR-2, which is greater than paclitaxel's affinity of −8.2 kcal mol^−1^ with VEGFR-2.^163^

M. Ihsan Han *et al.* (2021) came up with a new set of (*S*)-naproxen hydrazide-hydrazones that had strong inhibitory effects on VEGFR-2. Out of all, compound 81 was discovered to have the most effectiveness in inhibiting the growth of two types of human breast cancer cells (MDA-MB-231 and MCF-7). It also showed good selectivity, with IC_50_ values of 22.42 μM and 59.81 μM for each cell line, respectively. The incorporation of a trifluoromethoxy group at the second position enhances the activity but significantly diminishes it when added at the third position. The synthesised compound 81 showed a free energy (Δ*G*) of −9.77 kcal mol^−1^ when interacting with the active site of VEGFR-2. Compound 81 had potent antineoplastic effects and significantly reduced tumour size in mice with the Ehrlich acid tumour model, at both low (60 mg kg^−1^) and high (120 mg kg^−1^) doses.^164^

### 4.13 Pthalazine motifs

Fathalla Khedr *et al.* (2021) developed and synthesised derivatives of 4-phenylphthalazin-1-amine. Compound 82 was discovered to be the most powerful compound for inhibiting VEGFR-2, with an IC_50_ value of 0.11 ± 0.02 μM. This IC_50_ value is nearly equal to the value of sorafenib, which is 0.10 ± 0.02 μM. Compound 82 effectively suppressed the growth of three cancer cell lines, HepG2, HCT116, and MCF-7, with IC_50_ values of 11.23 ± 1.1 μM, 10.12 ± 1.0 μM, and 13.92 ± 1.2 μM, respectively. These results were superior to those of sorafenib, which had IC_50_ values of 9.18 ± 0.6 μM, 5.47 ± 0.3 μM, and 7.26 ± 0.3 μM, respectively. The estimated binding mode of compound 82 closely resembles that of sorafenib, which exhibited an affinity value of −101.98 kcal mol^−1^ and generated four hydrogen bonds. The binding energy of compound 82 is around −100.55 kcal mol^−1^.^165^

### 4.14 Benzothiophene analogues

**Fig. 24:**
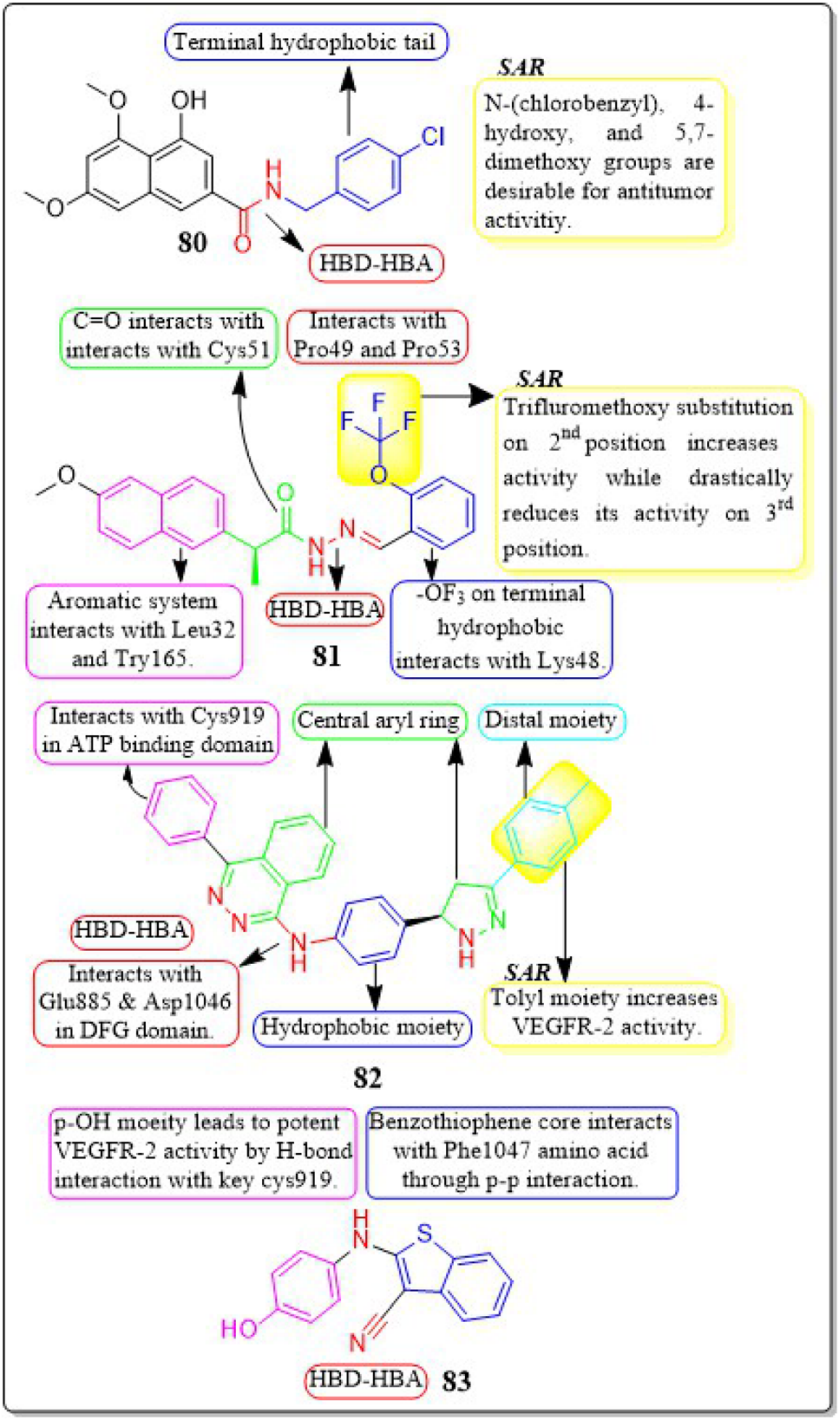
Pharmacophoric features and SAR of compounds 80 to 83.

Himalaya Singh *et al.* (2022) conducted experiments to assess the ability of cyanobenzo-thiophenes to suppress neovascularization in both *ex vivo* and *in vivo* angiogenic assays. Out of the compounds that were evaluated, derivative 83, which has a 4-hydroxyanilino substitution, demonstrated a high level of activity in inhibiting the formation of tubules and angiogenesis in HUVECs. This effect was observed at a concentration of 10 μM, ensuing in a complete halt of the process. The competitive binding experiment of compound 83 demonstrated its ability to prevent the phosphorylation of VEGFR2 induced by VEGF, resulting in the suppression of tubulogenesis. Furthermore, compound 83 effectively decreased the load in the xenograft model by suppressing the Akt/Src kinase activity and inducing reorganisation of the cytoskeleton in HUVECs (Fig. 24).^166^

### 4.15 Benzothiazole derivatives

Sahar M. Abou-Seri *et al.* (2021) introduced a new family of hydrazones based on isoxazole. Compound 84 showed great inhibition for VEGFR-2, with an IC_50_ value of 25.7 nM. This is slightly more effective than sorafenib, which had an IC_50_ value of 28.1 nM. The synthesised compounds were evaluated for their growth-inhibitory action against HepG2 cells. Compound 84 has shown superior efficacy with an IC_50_ value of 0.84 μM, surpassing the reference drug sorafenib with an IC_50_ value of 3.99 μM. The binding free energy for compound 84 at the VEGFR-2 binding site has been estimated to be −8.17 kcal mol^−1^. Compound 84's urea linker engages in H-bonding interactions with the crucial amino acids Glu-885 and Asp1046.^167^

Velma Ganga Reddy *et al.* (2019)^168^ performed the derivatization of pyrazolo-benzothiazole hybrids. Out of the compounds that were evaluated, compound 85 demonstrated noteworthy suppression of VEGFR-2 with an IC_50_ value of 97 nM. Compound 85 exhibits high potency against all examined cancer cell lines, with an IC_50_ in the range 3.17 μM to 6.77 μM. It performs even better than the reference medication axitinib, which has an IC_50_ in the range of 4.88 μM to 21.7 μM.

Hybrid molecule 85 exhibited the highest level of activity compared to the other compounds in the series. It displayed IC_50_ values of 3.17 μM (PC-3), 3.32 μM (HT-29), 3.87 μM (A549), and 6.77 μM (U87MG). Furthermore, it demonstrated greater activity than axitinib, which is a drug already used in clinical practice. Furthermore, compound 85 exhibited a selectivity towards cancer cells that was 9 to 15 times greater than that of axitinib. This clearly demonstrates the compound's high level of selectivity towards cancer cells. Compound 85 exhibited potent anti-angiogenic properties by effectively suppressing the development of intersegmental vessels in transgenic zebrafish.^168^

### 4.16 Benzodiazepines motifs

A new series of diazepam compounds with sulfonamide groups was synthesised and tested for their potential as anticancer activity by Nashwa M. Saleh *et al.* (2020). Compound 86 stood out as the most powerful derivative in its ability to inhibit VEGFR-2 at a concentration of (IC_50_ = 0.10 ± 0.01 μM), which is just as effective as sorafenib (IC_50_ = 0.10 ± 0.02 μM). It's worth noting that compound 86 demonstrated significant potency for the HepG2, HCT116, and MCF-7 cancer cell lines (IC_50_ = 8.98 ± 0.1, 7.77 ± 0.1, and 6.99 ± 0.1 μM, respectively). Compound 86 showed greater activity compared to sorafenib against HepG2 and MCF-7 cancer cell lines, with IC_50_ values of 9.18 ± 0.6 μM, 5.47 ± 0.3 μM, and 7.26 ± 0.3 μM, respectively. However, its activity against the HCT116 cell line was lower. Compound 86 was successfully docked at the ATP-binding site of the VEGFR-2 kinase enzyme, yielding a docking energy score of −116.78 kcal mol^−1^. The presence of the 4,6-dimethylpyrimidine moiety enhances its efficacy against VEGFR-2.^169^

### 4.17 Benzoxazole derivatives

**Fig. 25:**
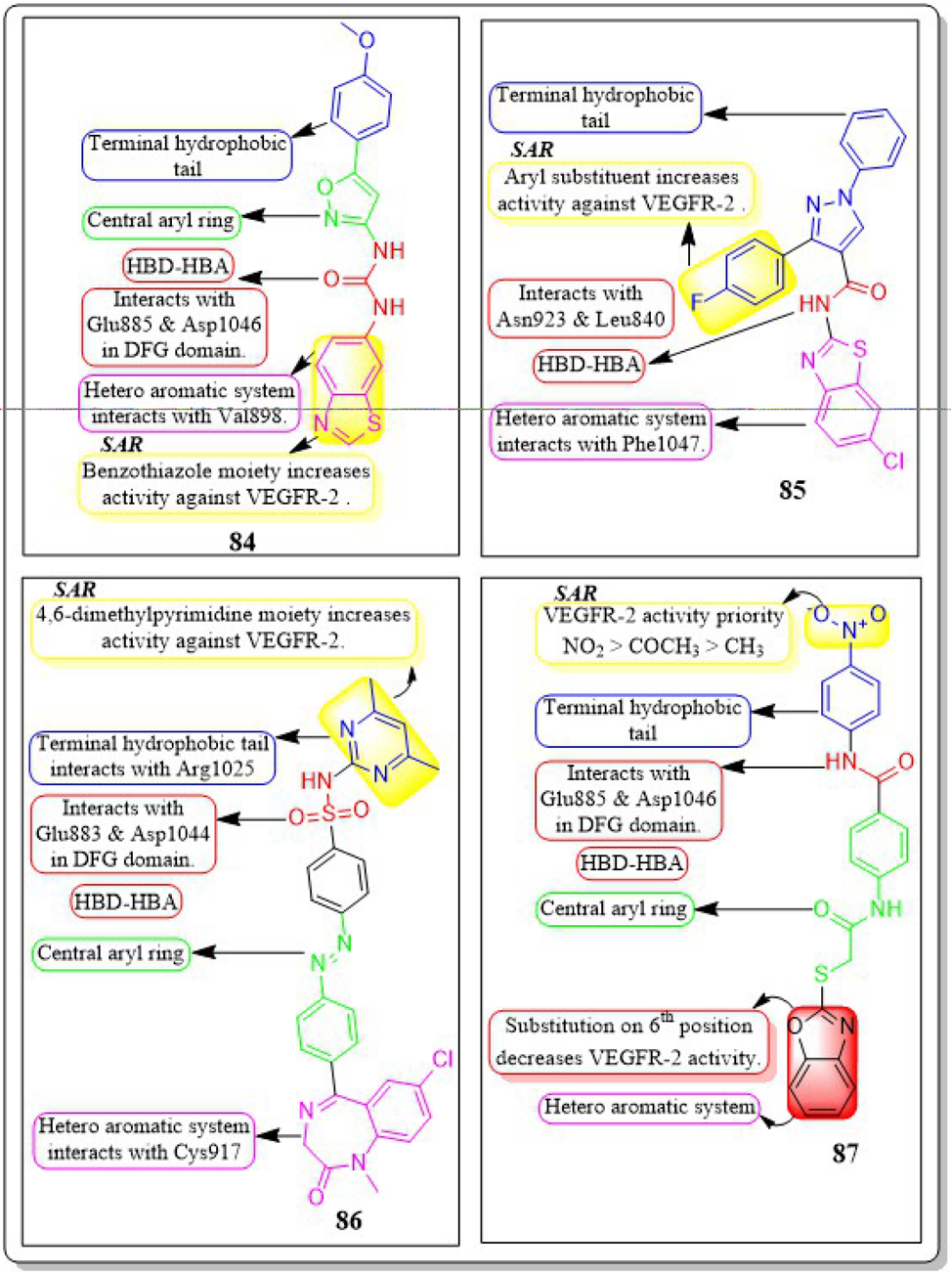
Pharmacophoric features and SAR of compounds 84 to 87.

Alaa Elwan and co-workers (2022) developed and synthesised benzoxazole-based compounds with the purpose of evaluating their effectiveness in inhibiting VEGFR-2 kinase and their potential as anticancer agents. Compound 87 had superior performance compared to sorafenib in both VEGFR-2 inhibition and anti-proliferative experiments, making it the most promising contender. The IC_50_ values for MCF-7, HCT116, HepG2 cell lines, and VEGFR-2 kinase were 3.43, 2.79, 2.43, and 0.0554 μM, respectively. However, the IC_50_ values of sorafenib were 4.21, 5.30, 3.40, and 0.0782 μM, respectively. Compound 87 demonstrated substantial suppression of TNF-a (90.54%) and IL-6 (92.19%) in comparison to dexamethasone (93.15%). The synthesised compound 87 exhibited a docking binding free energy of −7.65 kcal mol^−1^ against the VEGFR-2 active site (Fig. 25).^170^

### 4.18 Benzofuran analogues

A novel series of benzofuran derivatives was designed, synthesised, and evaluated by Omar A. El-Khouly *et al.* (2022). Compound 88 demonstrated potent inhibition of VEGFR-2, with an IC_50_ value of 68 nM. Compound 88 exhibited superior activity compared to other compounds against HePG2, MCF-7, HeLa, and PC3 cell lines, with IC_50_ values of 9.73 ± 0.7 μM, 11.58 ± 0.9 μM, 7.94 ± 0.5 μM, and 17.49 ± 1.3 μM respectively. It was discovered that the compound 88's docking score at the active binding site of VEGFR-2 was −7.0 kcal mol^−1^.^171^

### 4.19 Benzoquinone derivatives

Hayamitsu Adachi *et al.* (2021) developed vegfrecine a-pnalogues and assessed their effectiveness against VEGFR-2. Compound 89 was found to be a more powerful inhibitor than that of vegfrecine against the VEGFR-2 tyrosine kinase. Compound 89 demonstrated greater specificity for the VEGFR-2 kinase compared to VEGFR-1. The addition of halo- and alkoxy-substituents at the 5-position of the phenyl ring led to strong inhibition of the VEGFR-2 tyrosine kinases.^172^

### 4.20 Coumarin hybrids

In their study, Tahia K. Mohamed *et al.* (2021) successfully developed and synthesized novel derivatives of thiazolopyrazolyl coumarin. Compound 90 exhibited remarkable potency against VEGFR-2, with an IC_50_ value of 34 nM. Compound 90 exhibited remarkable cytotoxic activity for MCF-7, with an IC_50_ value of 5.41 μM, outperforming the reference drug doxorubicin (IC_50_ = 6.73 μM). The presence of the 4-chlorophenyldiazenyl moiety leads to increased VEGFR-2 activity. The binding pattern of compound 90 was enhanced by the hydrophobic interactions between the three moieties (*p*-chlorophenyl, phenyl, and dimethylaminophenyl) and the hydrophobic residues. This was explained by compound 90's superior docking score (−11.10 kcal mol^−1^) at the active site of VEGFR-2.^173^

**Fig. 26:**
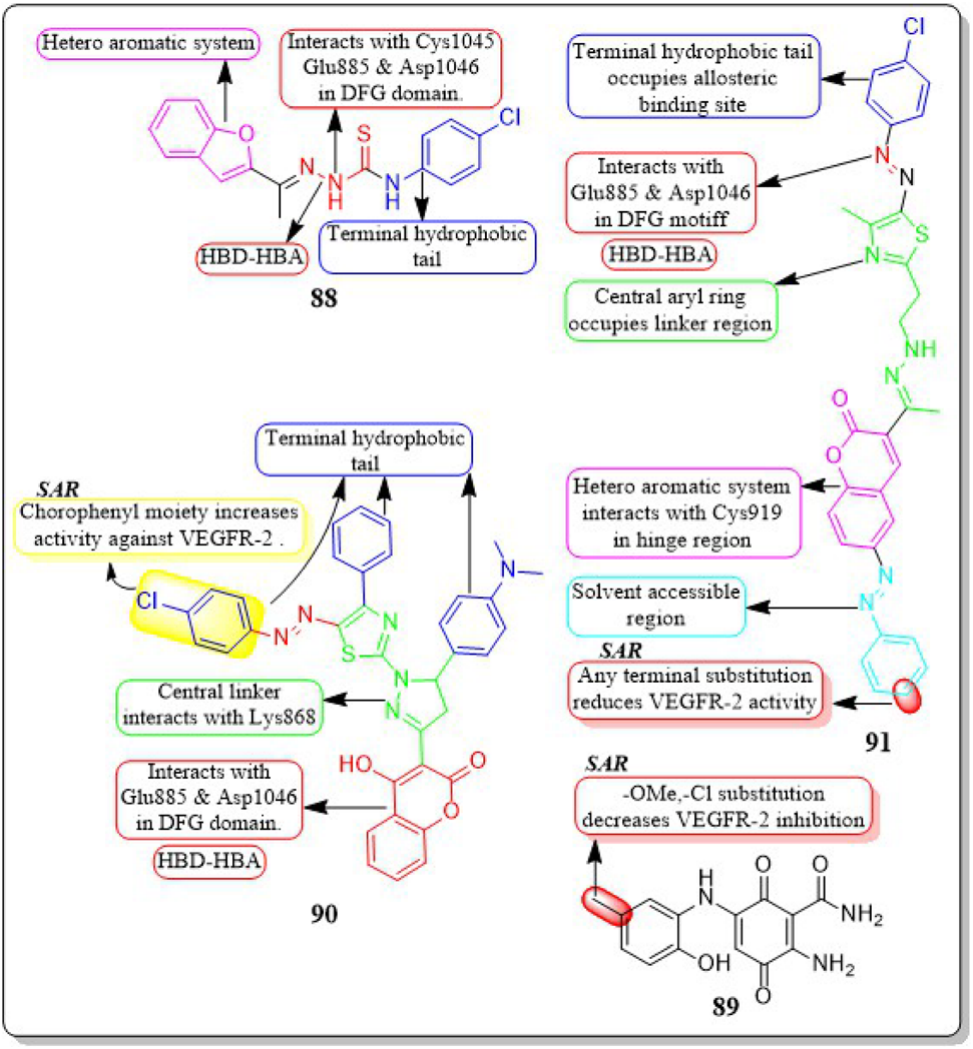
Pharmacophoric features and SAR of compounds 88 to 91.

A set of 3-thiazolyl-coumarins was modified and assessed by Tariq Z. Abolibda *et al.* (2023). Among all the compounds tested, 91 showed significant potential for inhibiting cancer growth in MCF-7 cells, with an IC_50_ value of 11.2 ± 0.80 μM. The molecular docking studies of the resulting derivatives were evaluated against VEGFR-2 and exhibited activities similar to sorafenib's, with compound 91 displaying the best binding score (−9.900 kcal mol^−1^). Substituting the solvent-accessible terminal phenyl ring leads to a decrease in VEGFR-2 activity (Fig. 26).^174^

### 4.21 Miscellaneous

#### 4.21.1 Furan analogues

Mohamed H. Hekal and his co-workers (2021) developed a collection of new compounds, *N*-(1,3,4-thiadiazol-2-yl)furan-2-carboxamide derivatives. Compounds 92 and 93 exhibited the most advantageous orientation to VEGFR-2 based on the docking experiments. Additionally, they were the most effective inhibitors of the receptor, with IC_50_ values of 7.4 ± 0.8 nM and 7.6 ± 0.4 nM, respectively. The antiproliferative properties were assessed towards three human epithelial cell lines: breast (MCF-7), colon (HCT-116), and prostate (PC-3) using the MTT assay technique employing doxorubcin as a reference standard. The free energy of binding for compounds 92 and 93 at the VEGFR-2 binding site has been estimated to be −55.90 kcal mol^−1^ and −67.65 kcal mol^−1^, respectively. The presence of the carbonyl group in the carboxamide moiety is considered essential for the binding process and plays a role in enhancing the affinity of compound 92.^175^

#### 4.21.2 Pyrazole derivatives

Fa-Qian Shen *et al.* (2019) synthesised a group of benzoyl amide compounds that contain a nitrogen heterocyclic ring. Compound 94 showed greater inhibition towards VEGFR-2, HeLa, A549, MCF-7, and HepG-2, with IC_50_ values of 0.34 ± 0.02 μM, 4.57 ± 0.30 μM, 15.57 ± 1.10 μM, 1.08 ± 0.06 μM, and 2.44 ± 0.15 μM, respectively. Substituting the chloro group with hydrogen on the phenyl ring that is connected to sulphonamide yielded a substantial decline in activity. The substitution of a toluyl group with a methyl group on the imidazole ring results in a comparable decrease in efficacy.^176^

#### 4.21.3 Thiazolidinedione hybrids

In a recent study, Neha Upadhyay and co-workers (2021) developed a new set of diarylpyrazoline-thiazolidinediones and conducted both *in vitro* and *in vivo* assays to evaluate their biological properties. The results demonstrated that compound 95 showed great inhibition, with an IC_50_ value of 5 μM against VEGFR-2. When an electron-donating group is placed to the *ortho*-position of the pyrazole ring, it enhances the activity. The anti-angiogenic potential of 95 was clearly demonstrated through various assays, including HUVEC proliferation, migration, and tube formation. The *in vivo* assay showed a significant potency of 95 in reducing neovascularization in the developing CAM. Compound 95 exhibited superior scores on the active site of VEGFR-2 compared to its steric counterparts.^177^

**Fig. 27:**
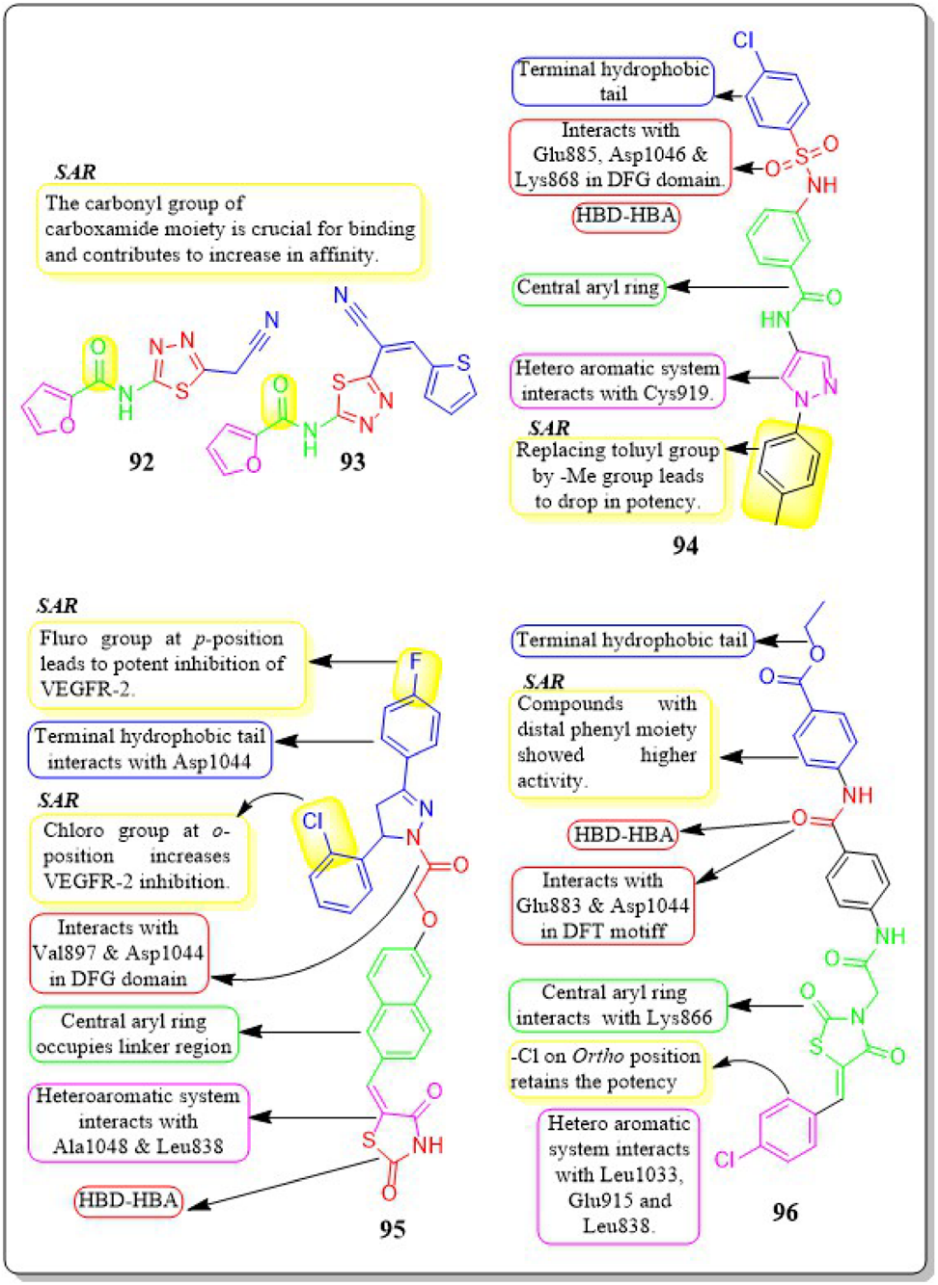
Pharmacophoric features and SAR of compounds 92 to 96.

In a new study, Khaled El-Adl *et al.* (2020) developed a set of thiazolidine-2,4-diones and tested their effectiveness on HepG2, HCT-116, and MCF-7 cells. Compound 96 exhibits similar activities to sorafenib against HepG2 cells, with IC_50_ values of 9.18 ± 0.6, 5.47 ± 0.3, and 7.26 ± 0.3 μM, respectively. However, it shows lower inhibition for HCT-116 cells and slightly higher inhibition for MCF-7. Compound 96 showed strong inhibition of VEGFR-2, with IC_50_ values of 0.17 ± 0.02 μ. Compounds containing the 2,4-dichlorobenzylidene moiety demonstrated greater VEGFR-2 inhibition activities compared to those containing the 4-chlorobenzylidene moiety. The distal phenyl group, which had ethyl ester substitutions in either 2,4-dichlorobenzylidene or 4-chlorobenzylidene derivatives, showed the most potent activities with an IC_50_ value of 0.17 ± 0.02 μM. Compounds containing a distal phenyl group, like 96, exhibited greater activity compared to those with a distal aliphatic group. The binding modes of compounds 96 closely resemble those of sorafenib, with affinity values of −101.17 kcal mol^−1^ and −101.14 kcal mol^−1^, forming 5 and 6 H-bonds, respectively (Fig. 27).^178^

#### 4.21.4 2-Thioxoimidazolidin-4-ones derivatives

A novel series of 2-thioxoimidazolidin-4-ones was conceived and developed by Ahmed A.E. Mourad *et al.* (2021). Compounds 97 and 98 demonstrated higher inhibitory activity against VEGFR-2 (IC_50_ = 25.14 ± 1.9 nM and 19.78 ± 1.3 nM, respectively) compared to sorafenib (IC_50_ = 35.62 ± 2.2 nM). Compounds 97 and 98 exhibited superior inhibition compared to sorafenib and erlotinib towards the MCF-7, HepG2, and A549 cell lines. The inhibitory activity of compounds 97 (1.63 ± 0.03 μM, 2.44 ± 0.13 μM, and 1.27 ± 0.04 μM, respectively) and 98 (2.26 ± 0.12 μM, 5.18 ± 0.23 μM, 3.14 ± 0.15 μM, respectively) was found to be greater than that of sorafenib and erlotinib.^179^

#### 4.21.5 1,2,4-Triazole analogues

Mohammed K. Abdelhameida *et al.* (2020) developed a set of new azole compounds that were synthesized and tested for their effectiveness against tumours. Compound 99 exhibited greater activity than the other compounds in terms of VEGFR-2 expression, β-TUB polymerization, and inhibition of the HepG2 cell line (IC_50_ = 19.82 ± 1.72 nM, 88.74 ± 9.27 μM, and 0.24 ± 0.06 μM, correspondingly). Compound 99 exhibited two hydrogen bond interactions with the amino acid Lys868, as well as a hydrophobic interaction.^180^

#### 4.21.6 1,3,4-Thiadiazole motifs

A study conducted by Saad R. Atta-Allah *et al.* (2021) involved the synthesis and bio-evaluation of 1,3,4-thiadiazols. Compound 100 exhibited remarkable inhibitory activity against VEGFR-2, with an IC_50_ value of 8.2 nM, surpassing the potency of pazopanib (IC_50_ = 9.7 nM). The cytotoxic activity of compound 100 against HepG-2, MCF-7, HCT-116, and PC-3 cancer cell lines showed promising results (4.22 ± 0.94 μM, 8.45 ± 0.75 μM, 33.14 ± 6.52 μM, 7.76 ± 0.6 μM) when compared to the reference standard pazopanib. The ligand's binding mode displayed an energy of −9.363 kcal mol^−1^. Derivative 100 exhibited three hydrogen bond interactions with Glu 885 and Asp 1046, along with a hydrophobic interaction with Lys 868.^181^

#### 4.21.7 *N*-Acylhydrazone derivatives

**Fig. 28:**
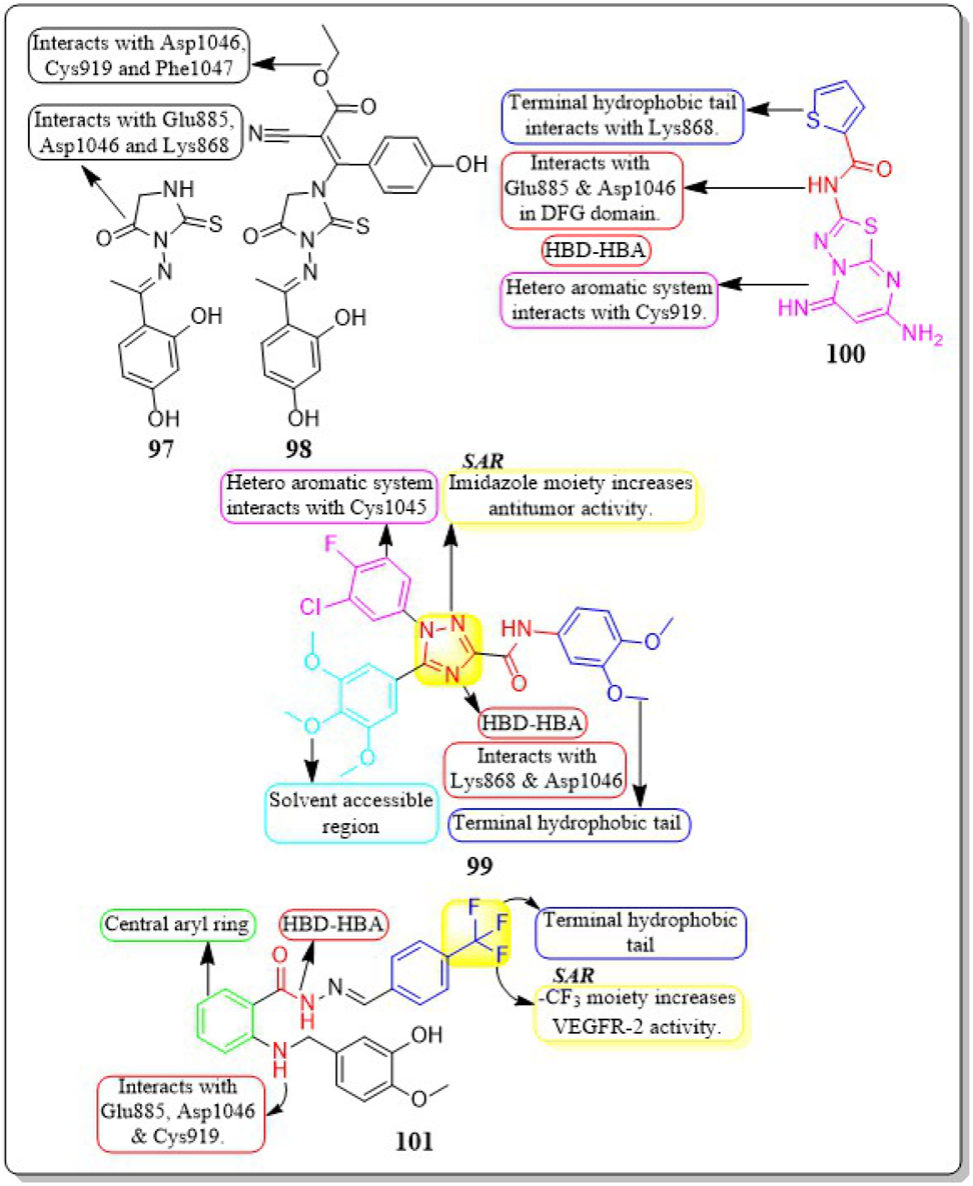
Pharmacophoric features and SAR of compounds 97 to 101.

In their study, Fernanda P. Pauli *et al.* (2020) examined a modified N-acylhydrazone structure and evaluated its effectiveness in inhibiting VEGFR-2 activity. The derivative 101, which has a trifluoromethyl substituent on the *para* position of the phenyl group, successfully suppressed neovascularization caused by VEGF in the CAM experiment. The tube generation experiment conducted on HUVECs demonstrated a beneficial impact of compound 101 on the production of new blood vessels (neovascularization) (Fig. 28).^182^

## 5. Recent patents

### 5.1 WO2023040996A1

**Fig. 29:**
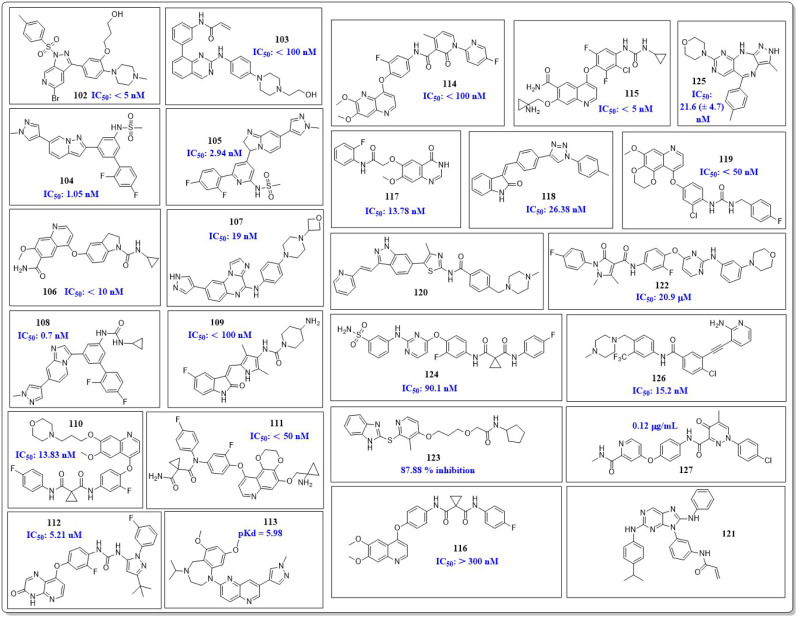
Compounds 102 to 127 with their VEGFR-2 activity from recent patents.

This patent explores an azaindazole macrocyclic compound about its effect of inhibiting the activity of a plurality of protein kinases, including HPK1, FLT3, and KDR (VEGFR-2). Compound 102 displayed inhibitory activity obtained using the four-coefficient nonlinear fitting formula with an IC_50_ around ≤5 nM against VEGFR-2 along with HPK1 and FLT3. Compound 102 also surpassed the MV-4-11 cell viability test with an EC_50_ of 3.8 nM, calculated by GraphPad Prism 5.0 software (Fig. 29).^183^

### 5.2 US20220354864A1

This patent contains essential information regarding substituted quinazolines for inhibiting kinase activity against EGFR, EGFR mutants, FGFR1 and 2, BTK, KDR (VEGFR-2), and KAK3. Compound 103 showed VEGFR-2 inhibition at an IC_50_ lower than 100 nM. Compound 103 significantly inhibited cancer cell lines: A549, A431, H1299, HCC827, H3255, and H1975.^184^

### 5.3 US20220315581A1

The current patent provides an explanation for fused ring compounds as FGFR and VEGFR dual inhibitors. Compound 104 exhibited excellent inhibitory activity of IC_50_ of 1.05 nM, 2.86 nM, and 2.66 nM against VEGFR-2, FGFR-1, and FGFR-2, respectively. IC_50_ data was obtained by parametric curve fitting (GraphPad Software). The compound 104 displayed a significant inhibitory effect on cell proliferation in the SNU-16 cell activity test with an IC_50_ of 14.5 nM. Upon a pharmacokinetic study, it was found that compound 104 can quickly reach a peak and exhibit a high oral absorption bioavailability of 73.2% after oral administration.^185^

### 5.4 US20220267324A1

This innovation discusses pyridine derivatives as FGFR and VEGFR dual inhibitors. The compound 105 demonstrated strong VEGFR-2 and FGFR-2 inhibition with IC_50_ = 2.94 nM and 3.22 nM, respectively. The pharmacokinetic activity revealed that the oral absorption bioavailability of compound 105 is 59.3%. The compound 105 shows significant anti-tumour activity of 69% against the SNU-16 cell line.^186^

### 5.5 US20210188806A1

This patent encompasses the process of preparing and the medical applications of the indoline-1-formamide substance. Compound 106 demonstrated potent inhibition of VEGFR-1, VEGFR-2, and VEGFR-3, with IC_50_ values below 10 nM and from 10 nM to 100 nM. The IC_50_ value of the substance is determined using the XLfit software (ID Business Solutions Ltd., UK) based on 8 concentration points.^187^

### 5.6 KR20220130747A

The patent informs in detail about 1*H*-pyrazole derivatives and their uses as Syk and VEGFR-2 dual target inhibitors. Compound 107 inhibited VEGFR-2 and Syk with IC_50_ = 19 nM and 17 nM, respectively. The compound has a high ocular blood ratio and is suitable for ocular administration. Compound 107 showed statistically significant drug efficacy in the scopolamine-induced mouse dry eye model.^188^

### 5.7 CN113490667A

This patent contains information about imidazopyridine derivatives as dual FGFR and VEGFR inhibitors. Compound 108 inhibited VEGFR-2 with IC_50_ = 0.7 nM. The compound 108 displayed more excellent SNU-16 cell activity (3–5-fold) than the control with IC_50_ = 10 nM. The compound of the present invention shows excellent tumour treatment effects at a lower dose in preclinical animal models.^189^

### 5.8 JP2021535931A

The pyrrole-substituted analogues were synthesized and tested for their potential for inhibitory activity against KDR (VEGFR-2), FLT3, and its mutants. Compound 109 is FLT3, FLT3-ITD, showed strong inhibitory activity against all FLT3D835Y, PDGFRβ, c-Kit, RET, KDR, showed certain inhibitory activity against AXL, *etc.*, IC_50_ >100 nM, which was a selective FLT3 inhibitor.^190^

### 5.9 WO2018059022A1

The patent has explored the biological activities of multiple signal transduction kinases, such as C-MET and KDR. Compound 110 inhibits VEGFR-2 and C-MET (IC_50_ = 1.83 nM and 4.4 nM, respectively). The compound 110 successfully inhibited HCC78, U87MG, HUVEC, and MKN-45 cell lines (IC_50_ = 0.157 μM, 0.436 μM, 0.292 μM, and 1.015 μM). Compound 110 similarly inhibited SK-OV-3, HCT-116, and A549 cell lines with IC_50_ = 4.326 μM, 0.786 μM, and 1.881 μM, respectively.^191^

### 5.10 EP3750893B1

The patent reveals information about the dioxazoline compound and its potential for inhibiting VEGFR-2 and C-MET. The compound 111 inhibits the C-MET, VEGFR-2, and MHCC97H cell lines with an IC_50_ <50 nM.^192^

### 5.11 US20220135544A1

This invention pertains generally to the capability of pyridopyrazinone derivatives and their potential for inhibitory activity against multiple tyrosine kinases such as KDR, MET, CRAF, EGFR, PDGFR-α, PDGFR-β, FGFR-1, and Src. Compound 112 inhibited all those kinases involved in resistance to BRAF inhibitors. The VEGFR-2 inhibition of compound 112 was moderate, with IC_50_ = 0.12 μM.^193^

### 5.12 US011542247B2

This patent demonstrates that compounds of 1,4-benzodiazepines have the capability to inhibit FGFR domains and VEGFR-2. Compound 113 exhibited inhibitory effects on VEGFR-2 and FGFR-1, FGFR-2, FGFR-3, and FGFR-4 in an enzyme binding experiment conducted using KINOMEscan®. The compound 113 demonstrated pKd values of 5.98 and 6.85, 6.03, 6.53, and 6.72 for each respective target.^194^

### 5.13 US20230151003A1

This patent has explored the potential of naphthyridine derivatives against C-MET, Mer, KDR, and Axl. The compound 114 successfully inhibited all these kinases with an IC_50_ < 100 nM.^195^

### 5.14 US20230124784A1

This patent deals with the potential of quinoline derivatives against different kinases such as VEGFR-1, VEGFR-2, VEGFR-3, FGFR-1, and RET. Compound 115 inhibited VEGFR-1/2/3 and RET with IC_50_ <5 nM. Along with VEGFR kinases, compound 115 also inhibited FGFR-2 with an IC_50_ <50 nM. The cell line studies revealed that compound 115 inhibited the following cell lines BXPC3 (IC_50_ <5 μM), A549 (<2.5 μM), Caki-1 (<2.5 μM), Hep3B2.1–7 (<2.5 μM), SUN16 (<5 μM), HeLa (<5 μM), k562 (<5 μM), PC-3 (5–10 μM), and hERG (>30 μM) cells.^196^

### 5.15 WO2020154610A

Compound 116 is one of a series of substituted quinoline compounds that the patent describes as kinase inhibitors that target many kinases, including Axl, Mer, c-Met, and VEGFR-2. The IC_50_ values for the compounds in this disclosure fell within the following ranges: Axl's IC_50_ is less than 10 nM, Mer's is between 10 and 100 nM, c-Met's is between 100 and 300 nM, and KDR's (VEGFR-2) is greater than 300 nM. The outcomes of a test to determine the metabolic stability of liver microsome tissues from humans, mice, rats, and dogs were also provided.^197^

### 5.16 CN110903253A

Compound 117, derived from chalcones, has been developed as a multi-target receptor tyrosine kinase inhibitor in this invention. Compound 117 displayed greater inhibitory activity against EGFR, VEGFR-2, and FGFR1 kinases, with IC_50_ values of 8.35 nM, 13.78 nM, and 18.42 nM, respectively. Compound 117 demonstrated inhibitory effects on cell lines including MCF-7, A549, and K562, with IC_50_ values of 9.27 μM, 9.89 μM, and 7.61 μM, respectively.^198^

### 5.17 CN111153889A

This invention presents a range of 2-indolone-triazole compounds that have demonstrated potential as antitumour agents. Compound 118 demonstrated significant inhibition of human VEGFR-2 kinase and H460 cell lines, with an IC_50_ value of 26.38 nM compared to 223.0 nM for the reference drug sunitinib. It also effectively suppressed the proliferation of the human lung cancer H460 cell line, with an IC_50_ value of 1.02 μM in contrast to 3.65 μM for sunitinib.^199^

### 5.18 WO2020042972A1

This invention details the synthesis of dioxane and quinazoline or quinoline compounds that are connected to a urea-substituted aromatic ring, serving as inhibitors for VEGFR-2 and CSF1R. Compound 119 demonstrated potent inhibitory activity against VEGFR-2 kinase and M-NFS-60 cell proliferation, with IC_50_ values below 50 nM and 100 nM, respectively.^200^

### 5.19 CN111138426A

This patent describes a group of indazole compounds that act as inhibitors of kinases. These compounds can be used to prevent or treat disorders that are dependent on kinase activity, such as gastrointestinal tumours. Compound 120 had the highest level of effectiveness among the synthesized compounds in suppressing the activity of c-KIT, FLT3, PDGFR-α, PDGFR-β, and/or VEGFR2 kinases.^201^

### 5.20 CN109384788A

Purine derivatives manufacture and use as anticancer agents were revealed in this invention. The synthesised compounds showed potent inhibitory actions that target VEGFR2 and EGFR. Compound 121 among the examined substances reduced the development of the HCC827 and H1975 cell lines with IC_50_ values under 100 nM.^202^

### 5.21 CN107286140A

This patent reveals information about cabozantinib derivatives and their activity against multiple kinases. Compound 122 inhibited C-MET, EGFR, and VEGFR-2 with IC_50_ = 7.44 nM, 0.81 nM, and 20.9 μM, respectively as compared to that if cabozantinib's IC_50_ of 60.9 nM, 74.8 μM, and 3.74 nM, respectively. Upon cell line investigation, it was observed that the compound without terminal fluoro substitution exhibited better activity against HCT-116, Caki-1, HepG2, PC-3, PANC-1, and MRC-5 cell lines with IC_50_ = 1.37 μM, 0.53 μM, 1.34 μM, 1.33 μM, 1.00 μM, and 281.7 μM.^203^

### 5.22 CN110003176A

This patent includes details about benzimidazole derivatives and their inhibitory potential against different kinases, such as BRaf, VEGFR-2, PDGFR-β, and TOPK. The compound 123 inhibited BRaf, VEGFR-2, and PDGFR-β with significant inhibition of 90.96%, 87.88%, and 88.67%, respectively. Further cell line studies against A549, HCT116, and PC-3 cell lines disclosed that the most potent compound displayed better inhibitory activity (IC_50_ = 1.14 μM, 1.67 μM, and 2.64 μM) than reference standard, sorafenib (IC_50_ = 2.12 μM, 2.25 μM, 3.60 μM).^204^

### 5.23 CN110386901A

This patent discloses information about pyrimidine-sulphonilamide hybrids and their ability to inhibit multiple kinases. Compound 124 successfully inhibited c-Met, VEGFR-2, and EGFR with IC_50_ = 4.32 nM, 6.91 nM, and 58.6 nM, respectively as compared to reference standard cabozantinib (IC_50_ = 61.2 nM, 74.5 nM, and 3575 nM). The compound 124 also inhibited HCT-116, Caki-1, HepG2, PC-3, PANC-1, and hERG cell lines with IC_50_ = 2.09 μM, 0.86 μM, 6.98 μM, 0.86 μM, 7.61, μM and >30 μM, respectively, better than reference standard Cabozantinib (4.32 μM, 6.26 μM, 11.39 μM, 7.54 μM, 5.50 μM, 28.2 μM respectively).^205^

### 5.24 CN109020980A

Pyrazolo-pyrimido-diazepine derivatives were explored as Aurora and VEGFR-2 kinase inhibitors in this patent. The compound 125 inhibited aurora-A/B and KDR with IC_50_ = 46.2 ± 2.2 nM, 37.6 ± 13.3 nM, and 21.6 ± 4.7 nM. Cell line studies revealed that compound 125 inhibited MKN-45, MKN-74, SGC-7901, BGC-823 cell lines with IC_50_ = 1255.42 ± 558.98 nM, 3137.86 ± 408.39 nM, 10 μM, and 10 μM, respectively.^206^

### 5.25 CN108456163A

This patent discovers about piperazine derivates and their potential for inhibitory action against FGFR1, RET, and KDR. Compound 126 displayed inhibitory activity against FGFR1, RET, and KDR with IC_50_ = 45.1 nM, 40.1 nM, and 15.2 nM, respectively.^207^

### 5.26 CN109096250A

The invention investigated the inhibitory potential of 4-phenoxypyridine-pyridazinone derivatives on VEGFR-2. The compound 127 had a VEGFR-2 inhibitory activity (IC_50_) of 0.12 μg mL^−1^, making it the most powerful derivative. The compound 127 showed stronger inhibitory effects on the BGC-823, MKN-45, H460, HT-29, and A549 cell lines compared to the reference standard sorafenib. The IC_50_ values (in μg mL^−1^) for compound 127 were 0.82, 1.29, 2.49, 3.54, and 0.37, respectively, while the IC_50_ values (in μg mL^−1^) for sorafenib were 1.51, 1.43, 1.73, 2.01, and 1.25, respectively.^208^

## 6. Essentials for developing VEGFR-2 inhibitor

The VEGFR-2 kinase has been extensively investigated as a cancer target over the past decade, with a wide variety of distinct structural motifs. Although many compounds have been synthesized to block VEGFR-2 at micromolar concentration, there is still potential for the development of more powerful molecules. While gathering data for this review, we noticed a consistent trend in the molecules. Heterocyclic nuclei that contain nitrogen as a heteroatom are commonly observed to have a higher prevalence in inhibiting VEGFR-2. The Asp-Phe-Gly (DFG) motif plays an important role in the regulation of VEGFR-2 and most of kinase activity. The solvent accessibility of the molecule is enhanced by further substitution on the heterocyclic nucleus utilizing methyl/ethyl ester, nitro, and 1-(ethoxymethyl)cyclopropan-1-amine group. The core aromatic ring connected to a heterocyclic aromatic ring enhances the Pi–Pi interaction of the molecule. The pharmacophore typically consists of an amide linkage, which acts as a hydrogen-bond acceptor and donor group. Its bio-isosteres are typically connected adjacent to the core aromatic ring. The terminal hydrophobic tail of the molecule often consists of an aromatic ring with various replacements, such as halogens, ether, nitro groups, and so on.

Quinazolines have consistently outperformed the reference standard sorafenib in inhibiting VEGFR-2 during the experiment. The quinoxalines, indole, thiazolidinediones, and benzothiazoles analogues are heterocyclic aromatic systems that exhibited superior inhibition of the VEGFR-2 enzyme compared to the reference drugs. The compounds that showed considerable activity against VEGFR-2 included a terminal hydrophobic tail portion consisting of methoxyphenyl, trimethoxyphenyl, trifluromethyl halogen/alkyl/nitro modified aromatic moieties. The core aryl linker, which connects heteroaromatic systems and HBD–HBA, consists of an aromatic ring (ideally benzene) and an amide bond, with an optimal spacing of three to five atoms. Molecules bearing the amide and cyclopropane-1,1-dicarboxamide linkage, acting as hydrogen bond donor–hydrogen bond acceptor, have been found to be more effective VEGFR-2 inhibitors compared to those with sulphonamide or thioamide linkages. Although most effective compounds follow a general pattern, there are notable exceptions, such as fused molecules (PROTAC degraders), that show strong VEGFR-2 inhibitory action.

## 7 Conclusions

While the fight against cancer is ongoing, the inhibition of angiogenesis has become a viable approach for cancer treatment with the identification of new genes, transcription factors, signalling pathways, and mechanisms linked to it. VEGF and VEGFRs are the key component in controlling angiogenesis. As cancer progresses, VEGF has been found to be widely distributed and overexpressed. When VEGF binds to the protein kinase VEGFR-2, it triggers the production of blood capillaries and mediates the signalling pathway. Blocking VEGFR-2 signalling is therefore seen to be one of the most promising ways to prevent tumour-induced angiogenesis. Although many drugs targeting VEGFR-2 has been approved by USFDA for the treatment various cancers; they evolved with their own set of side effects and drug resistance over the course of treatment and hence there is still an urgent need of more effective anticancer molecules.

This review focuses on the structure of VEGFR-2, physiological role, and involvement of VEGF/VEGFR-2 system in the onset and progress of cancer. The design and structure–activity relationship of small-molecule VEGFR-2 inhibitors published through papers and patents in last five years is discussed that have shown improved anticancer attributes in recent years. The efforts taken by researchers to enhance the potencies of the molecules towards inhibiting VEGFR-2 in comparison to reference standard drugs such as sorafenib, sunitinib, or pazopanib is focused. The structural framework features requisite for developing new drug candidate are well explored in the present paper. The Asp-Phe-Gly (DFG) motif plays an important role in the regulation of VEGFR-2 and most of kinase activity. Heteroaromatic system is required for the interaction with crucial amino acid residues such as Cys919, Asp1046, Asn921, and/or Glu885 within the ATP-binding domain of VEGFR-2. The pharmacophoric element in majority ligands typically consists of an amide linkage, which acts as an HBD–HBA region interacts with Glu885 and Asp1046 in DFG domain of target receptor. Central aryl ring is necessary for occupying linker region and variety of bio-isostere could enhance the VEGFR-2 inhibition. Solvent-accessible region of the ligand molecule interacts with Asn923. Terminal hydrophobic tail consisting of an aromatic ring with variable substitutions occupies allosteric binding site. Five bond spacers between heteroaromatic region and HBD–HBA region in the molecule has always provided better result towards VEGFR-2 inhibition mirroring the behaviours of sorafenib or reference standard. Although most effective compounds follow a general pattern, there are notable exceptions, such as fused molecules (PROTAC degraders), that show strong VEGFR-2 inhibitory action.

This paper will help prospective synthetic and medicinal chemists to explore VEGFR-2 inhibitors utilising a molecular or pharmacophore hybridization approach. Combining different molecular fragments and pharmacophoric features, researchers can enhance the potency, selectivity, and therapeutic efficacy of novel hybrid molecules against VEGFR-2 targeting various cancers. This strategy will allow the generations of a library of compounds with diverse chemical structures and properties, offering a wide range of options for optimizing anticancer or antiangiogenic drug candidates and overcoming resistance mechanisms.

## Abbreviations

VEGFR: Vascular Endothelial Growth Factor Receptor
KDR: Kinase Insert Domain Receptor
FLK-1: Foetal-Liver Kinase 1
HBD–HBA: Hydrogen Bond Donor–Hydrogen Bond Acceptor
MM-PBSA: Molecular Mechanics Poisson–Boltzmann Surface Area
MMGBSA: Molecular Mechanics Generalized-Born Surface Area
MD: Molecular Dynamic
DFG: d-Asp-Phe-Gly Motif
HUVEC: Human Umbilical Vein Endothelial Cells
bFGF/FGF2: Basic Fibroblast Growth Factor
CAM: Chorioallantoic Membrane
VHL: Von Hippel–Lindau
PROTAC: Proteolysis Targeting Chimera
PLC/PKC pathway: Phospholipase C/Protein Kinase C Pathway
Syk: Splenic Tyrosine Kinase
TIE2: Tyrosine Kinase with Immunoglobulin and Epidermal Growth Factor Homology Domains 2
USFDA: United States Food and Drug Administration
TK: Tyrosine Kinase
eNOS: Endothelial Nitric Oxide Synthase
PI3K: Phosphoinositide 3-Kinase
MAPK/ERK: Mitogen Activated Protein Kinase/Extracellular-Signal-Regulated Kinase
NCI: National Cancer Institute
TGI: Tumour Growth Inhibition
ELISA: Enzyme-Linked Immunosorbent Assay
SAR: Structure–Activity Relationship
p38MAPK: p38 Mitogen-Activated Protein Kinases
PI3K/PBK-Akt: Phosphoinositide-3-Kinase/Protein Kinase B-Akt
PLCγ: Phospholipase C Gamma
FLK: Focal Adhesion Kinase
PlGF: Placental Growth Factor
Src: Sarcoma Oncoprotein
Shc: Src Homology 2
Akt: Serine-Threonine Protein Kinase
IL-8: Interleukin 8
IL-6: Interleukin 6
Ras: Rat sarcoma (guanosine-nucleotide-binding protein)
Raf: Rapidly Accelerated Fibrosarcom
MEK1: MAPK/ERK Kinase
Erk1: Extracellular Signal-Regulated Kinase 1
PKB: Protein Kinase B
vWF: von Willebrand factor
BAD: BCL2 Associated agonist of Cell Death
DNA: Deoxyribonucleic Acid
V-FITC/PI: V-fluorescein Isothiocyanate (FITC)/Propidium Iodide (PI)
VHL-L: von Hippel–Lindau-like protein
mTOR: Mammalian Target of Rapamycin
β-TUB: β-Tubulin
TNF-α: Tumour Necrosis Factor Alpha
HPK1: Hematopoietic Progenitor Kinase
hERG: human Ether-a-go-go-Related Gene
FLT3: FMS-like Tyrosine Kinase 3
FLT3-ITD: FMS-like Tyrosine Kinase 3-Internal Tandem Duplication
FGFR-1: Fibroblast Growth Factor Receptor 1
C-MET: mesenchymalepithelial transition factor
EGFR: Epidermal growth factor receptor
PDGFR-α: Platelet-Derived Growth Factor Receptor Type alpha
RET: Rearranged During Transfection
Syk: Spleen tyrosine kinase
AXL: Anexelekto

## Data availability

No primary research results, software or code have been included and no new data were generated or analysed as part of this review.

## Author contributions

Prashant J. Chaudhari: funding acquisition, conceptualizing, data curation, supervision, formal analysis, validation, writing – original draft, writing – review, and editing. Aditya R. Nemade: data curation, formal analysis, writing – original draft Atul A. Shirkhedkar: validation, formal analysis, writing – review, and editing.

## Conflicts of interest

Authors declare no conflict of interest.
